# Climate-driven changes in zoonotic risk of arenaviral hemorrhagic fevers in South America

**DOI:** 10.1038/s44298-026-00189-2

**Published:** 2026-04-15

**Authors:** Pranav S. Kulkarni, Nuri Y. Flores-Pérez, Andie H. Jian, Brian H. Bird, Christine K. Johnson, Marcela Uhart, Pranav S. Pandit

**Affiliations:** 1 Department of Population Health and Reproduction, Weill School of Veterinary Medicine, University of California, Davis, CA USA; 2 One Health Institute, Weill School of Veterinary Medicine, University of California, Davis, CA USA; 3 Karen C. Drayer Wildlife Center, Weill School of Veterinary Medicine, University of California, Davis, CA USA

**Keywords:** Climate sciences, Diseases, Ecology, Ecology, Environmental sciences

## Abstract

Climate change is expected to significantly alter the ecological dynamics of zoonotic diseases, yet its long-term impact on rodent-borne hemorrhagic fevers in South America remains poorly understood. Here, we developed a robust predictive modeling framework that integrates species distribution models with a mechanistic force-of-infection approach to evaluate the effects of climate change on zoonotic risk of New World Arenaviruses. Using climate projections under Shared Socioeconomic Pathways SSP2-4.5 (moderate) and SSP5-8.5 (severe), our models predict a substantial increase in spillover risk across endemic and non-endemic regions over the next two decades. Projected increases in spillover risk for Guanarito, Machupo, and Junin viruses are primarily driven by climate-induced shifts in temperature seasonality, reduced precipitation, and expanding anthropogenic land use, particularly cropland and urban areas within reservoir habitats. Our projections identify transboundary arenaviral hotspots, underscoring the urgent need for coordinated climate-adaptive public health policies, including cross-border surveillance efforts, land-use planning, and resilient rural health systems.

## Introduction

Long-term changes in climate and extreme weather events are known to influence the spread of rodent-borne diseases^1–3^. Attributed to direct and indirect effects of climate change, over 3000 mammalian species, including rodents, are projected to shift their distribution by 2070^4^, potentially amplifying the transmission risk of rodent-borne diseases. Shifts in the distribution of rodent reservoirs of these zoonotic diseases are driven by changes in food availability, migration of rodent populations, climate suitability, and human population dynamics. Prior research shows that bioclimatic variables such as temperature, precipitation, and changes in land use have been instrumental in modeling the changing risk of high-consequence rodent-borne diseases such as Lassa fever, Argentine Hemorrhagic fever^5–8^ and Hantavirus^9^. Specifically, arenaviral spillover is strongly influenced by human-rodent contact and the distribution of rodent reservoirs^10,11^. Sarute and Ross estimated an increase in human–rodent interactions influenced by anthropogenic drivers and climate change^12^. It is estimated that the distribution of *Calomys musculinus*, the rodent reservoir of Junin virus (JUNV), which causes Argentine Hemorrhagic Fever (AHF), will undergo substantial changes in the future as a response to climate change^1,6^.

South American New World Arenaviruses (NWAs) include Guanarito virus (GTOV) in Venezuela and Colombia, Machupo virus (MACV) in Bolivia and Paraguay, and JUNV in Argentina. These viruses have caused multiple human outbreaks, with case fatality ranging from 5 to 30%^13^. Despite their inclusion on priority pathogen lists by global health agencies^10,14,15^, aspects such as disease dynamics, changes in reservoir distributions and factors associated with distribution changes have not been studied extensively^14^. In contrast, some of the Old World Arenaviruses (OWA), such as Lassa fever virus in Africa have been extensively studied in terms of disease dynamics and spillover risk^8,16,17^. The distribution of *Mastomys natalensis*, the known reservoir of Lassa fever, has been shown to be sensitive to factors such as temperature range, precipitation seasonality, co-presence of humans and rodent populations, and land-use patterns^8,17^. An extensive meta-analysis of literature on Lassa fever showed increased transmission risk in peri-urban settings that was associated with movement of rodents, humans, and climate change^18^. On the other hand, the impact of climate change on NWA spillover from rodents to humans remains poorly understood.

These climatic shifts, combined with anthropogenic land-use changes, could profoundly affect rodent populations and elevate the risk of arenaviral spillover^19^. In particular, increased wildfire frequency and intensity (driven by hot-dry conditions and land-use disruptions) create a post-fire environment that favors rapid repopulation by known rodent reservoirs of NWAs, a pattern linked to increased risk of human cases. Our previous study on risk of JUNV indicated that factors associated with changes in temperature, rainfall, and land-use could alter the habitat suitability of the rodent reservoir^6^. Rodent habitat suitability combined with human population density can shift the spatial and temporal patterns of human risk from NWAs^20^. Given the climate change paradigm, changing patterns of land-use and other anthropogenic activities, the spillover risk of NWAs needs to be quantified based on changes in rodent-human interactions and force-of-infection.

We hypothesized that the spillover risk of rodent-borne NWAs is closely linked to the species distribution of their rodent reservoirs, and that projected climatic changes in South America may facilitate their spread to previously non-endemic regions. We further expect that climate-driven changes in the distribution of the rodent reservoirs will be predictive of changes in NWA spillover risk to humans. In this study, we estimated a proxy for the spillover risk by quantifying the FOI, defined as the probability of successful transmission to humans, based on human-rodent interactions under different climate change scenarios. FOI was mechanistically modeled using species distribution patterns and estimated population densities of six known rodent reservoir: *Zygodontomys brevicauda* (GTOV), *Sigmodon alstoni* (GTOV), *Calomys callosus* (MACV), *Calomys musculinus* (JUNV), *Calomys laucha* (JUNV) and *Oligoryzomys flavescens* (JUNV). This integrated approach combines species distribution models (SDMs) with mechanistic FOI modeling under three climate scenarios: (i) the current climate, and two future climate scenarios based on SSPs^21^ (ii) SSP2-4.5 (moderate climate change scenario) and (iii) SSP5-8.5 (extreme climate change scenario) for the period 2041–2060.

## Results

### Hotspots for spillover risk of New World Arenaviruses will increase in the future

**Fig. 1:**
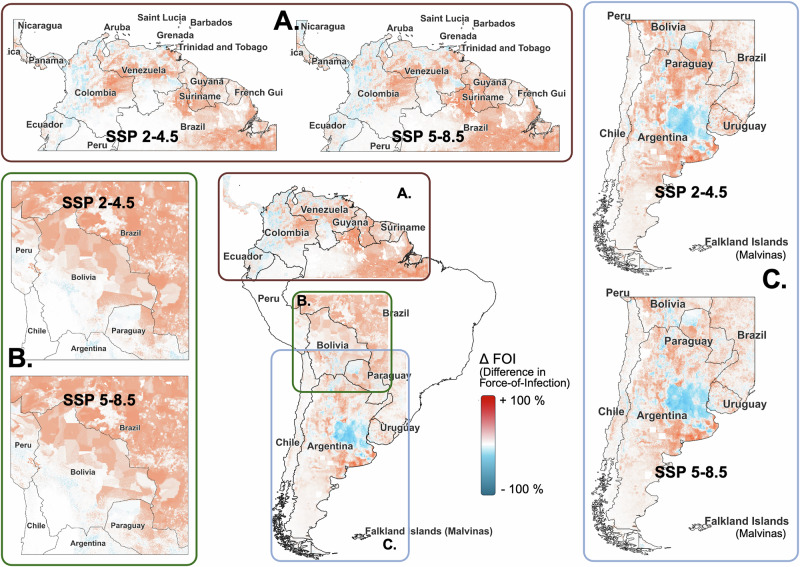
Changes in zoonotic risk of New World Arenaviruses (NWAs) represented by the difference in force-of-infection (FOI) estimates between the current climate and two projected climate change scenarios: SSP 2–4.5 (moderate climate change scenario) and SSP 5–8.5 (extreme climate change scenario). Central panel shows an overview of the three modeled viruses (an average change between current climate and the two SSP scenarios; only for illustration). Each subpanel shows the range of difference: blue = lower FOI and red = higher FOI). **A** Map of the difference between FOI estimates in future scenarios and the current climate for Guanarito virus (GTOV), which causes Venezuelan Hemorrhagic Fever (VHF), **B** Map of the difference in FOI estimates for Machupo virus (MACV), which causes Bolivian Hemorrhagic Fever (BHF), and **C** Map of the difference in FOI estimates for Junin virus (JUNV), which causes Argentine Hemorrhagic Fever (AHF).

**Fig. 2:**
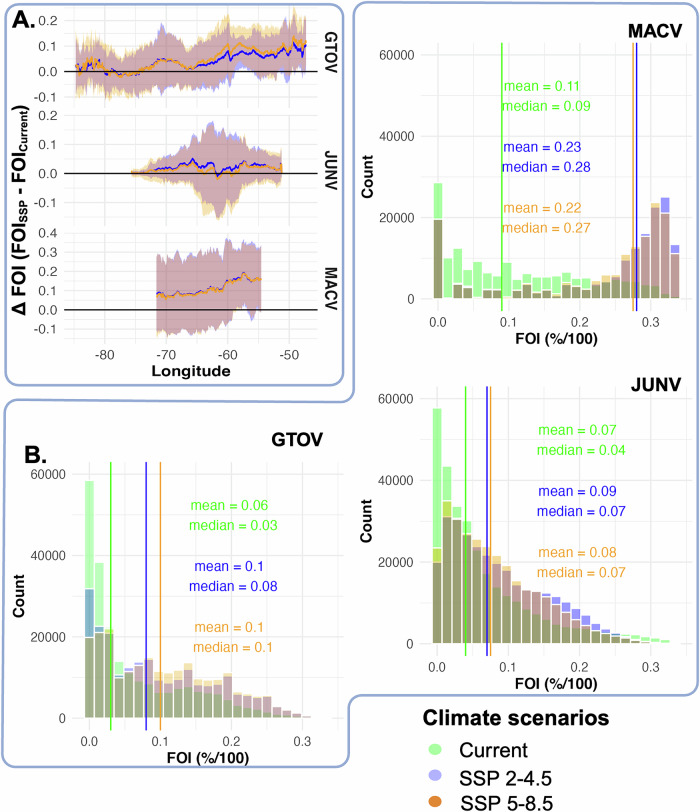
Descriptive statistics of the FOI estimates and their differences for all three climate scenarios (1 current; 2 Shared Socio-economic Pathways - SSPs). **A** Difference in FOI estimates across geographical longitude (west to east; x-axis) between current and SSP scenarios (y-axis) with confidence intervals (±1.96*SE) for the three NWAs. **B** Histograms of the FOI estimates for current and the two SSP scenarios for GTOV, MACV and JUNV (the vertical lines represent the median FOI estimates. Y-axis represents the count of pixels on the raster with resolution ≈ 0.042°).

Projected spatial risk profiles under both future climate change scenarios (SSP2-4.5 and SSP5-8.5) indicated more widespread and elevated spillover risk, represented by force-of-infection (FOI) as a proxy metric, henceforth) for all three NWAs compared to projections for current conditions (Fig. 1). Elevated FOI was also predicted in some regions where these NWAs are already endemic (Fig. 1). Overall, the two climate scenarios produced similar FOI patterns, with SSP5‑8.5 generally yielding smaller deviations from the current climate than SSP2﻿-4.5 (Figs. 1, 2).

In both climate scenarios, GTOV elevated FOI areas expanded beyond current endemic zones in Venezuela, with higher projected FOI in non‑endemic areas of Guyana, Suriname and Brazil (Fig. 1A). In contrast, endemic regions along Venezuela’s northern coast showed reduced FOI, while the interior habitats of GTOV reservoirs exhibited little change under either scenario (Fig. 1A). Some coastal endemic areas showed unchanged GTOV FOI under SSP2‑4.5 and slightly lower FOI under SSP5‑8.5 (Supplementary Fig. 1.4). For MACV, FOI was higher overall under both scenarios, with declines in endemic zones along the Bolivian Andes foothills and increased FOI in non‑endemic grasslands of Bolivia and Paraguay (Fig. 1B). JUNV showed reduced FOI in Argentina’s Pampas grasslands and higher FOI in the surrounding non‑endemic zones under both scenarios, with the magnitude of JUNV FOI change being greater under SSP5‑8.5 than under SSP2‑4.5 (Fig. 1C). Some endemic areas in Buenos Aires province showed little change, whereas FOI increased around the capital region, and non‑endemic regions along the borders of Argentina, Paraguay and Bolivia showed higher FOI in both scenarios, overlapping spatially with MACV high‑FOI zones (Fig. 1B, C).

For all three NWAs, average FOI increased under both SSP2‑4.5 and SSP5‑8.5 compared with the current climate (Fig. 2A, B). For GTOV and MACV, the increase in FOI was higher in eastern tropical portions of reservoir ranges than in the drier western regions separated by the Andes mountain range and other hilly areas (Fig. 2A). For MACV, this reflected a shift in FOI from the Andean foothills toward interior grasslands, yielding larger average increases in eastern Bolivia than in the higher‑altitude western regions (Fig. 2A). GTOV FOI increased by 5% and 8% (median values) under SSP2‑4.5 and SSP5‑8.5, respectively. For MACV, median FOI increased by 19% and 18% under SSP2‑4.5 and SSP5‑8.5, respectively, whereas JUNV FOI increased by 3% under both scenarios (Fig. 2B). Potential FOI hotspots (areas within 90th percentile of estimated FOI) for NWA spillover identified under current conditions persisted under both SSP scenarios (Supplementary Fig. 1.3).

### Changes in the NWA force-of-infection (FOI) were found to be associated with climate and land-use changes in the reservoir habitats

**Fig. 3:**
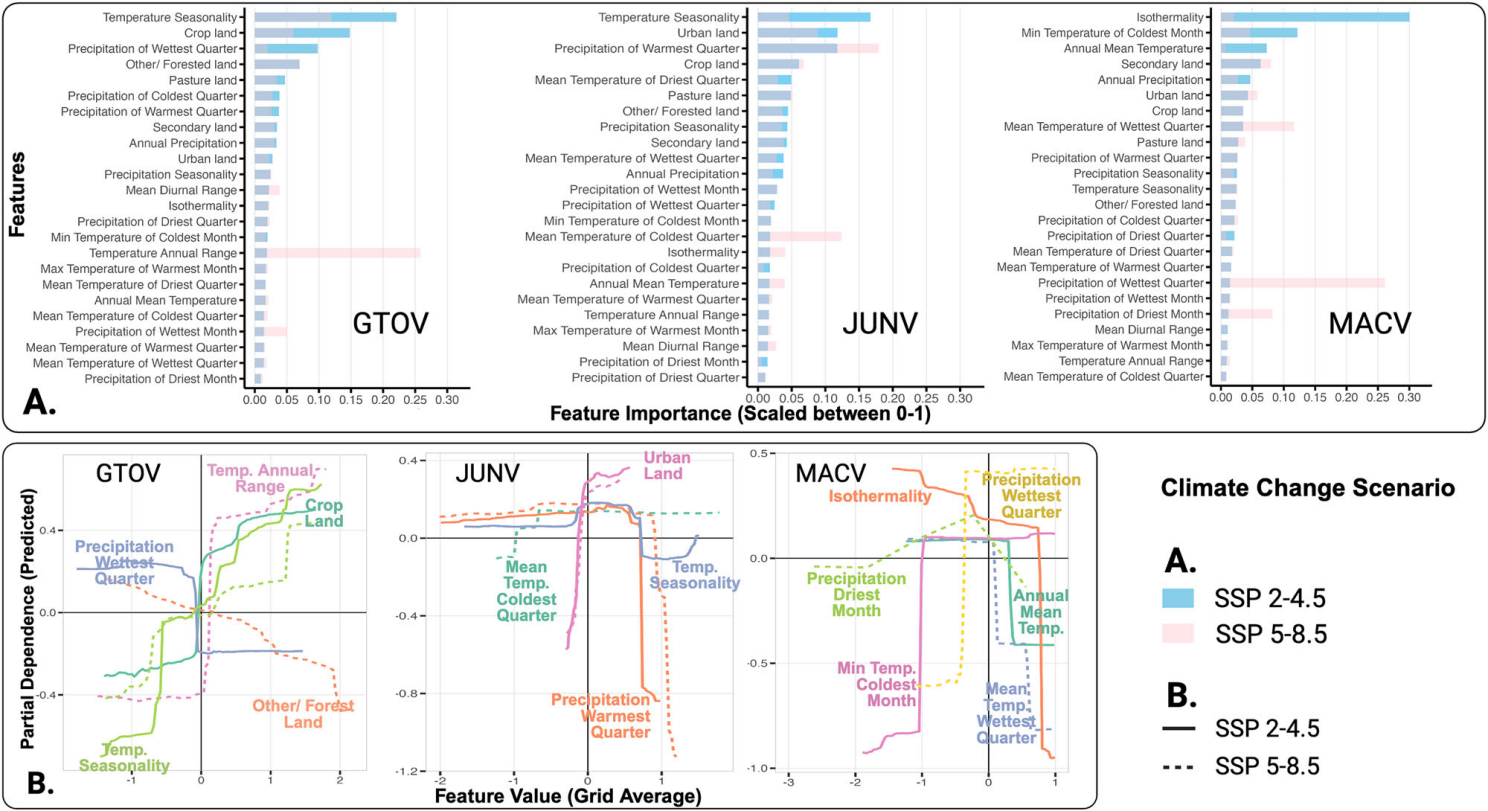
Association of changes in environmental features with changes in Force-of-Infection (FOI). **A** Importance of the different environmental features used for predicting the change in spillover risk represented by the estimated FOI **B** Partial dependence plots for the top three most important features of the three New World Arenaviruses (NWAs), GTOV, JUNV, MACV, for the moderate climate change (SSP2-4.5) and the extreme climate change (SSP5-8.5) scenarios compared to the current climate.

For all three NWAs, the predicted increase in FOI under both future climate change scenarios corresponded to higher temperature-related features (annual range, seasonality), reduction in precipitation-related features (precipitation in warmest quarter or in wettest quarter), expansion of urban and crop land, and contraction in forest land (Fig. 3A; Supplementary Table 3.1).

For GTOV, changes in FOI from the current to the future scenarios were found to be associated with features related to temperature, such as annual range and seasonality (Fig. 3A). For the top three features in the SSP 2-4.5 scenario model, the estimated increase in FOI corresponded with an increase in temperature seasonality and the presence of crop land. In contrast, the estimated decrease in FOI corresponded with an increase in precipitation of the wettest quarter of the year (Fig. 3B). For the SSP 5-8.5 scenario, the predicted change in FOI showed a positive association with changes in annual range and seasonal patterns of temperature. In contrast, an increase in the presence of forest land corresponded with a decrease in FOI (Fig. 3B).

For MACV, the changes in FOI were associated with changes in precipitation and temperature features. Considering the top three features for the SSP 2-4.5 scenario, the increase in FOI corresponded to a decrease in isothermality and annual mean temperature, and a slight increase in the minimum temperature of the coldest month (Fig. 3A). Under the SSP5-8.5 scenario, changes in FOI were positively associated with precipitation of the wettest quarter. A decrease in FOI corresponded with a decrease in the mean temperature of the wettest quarter.

For JUNV, the change in FOI corresponded to changes in temperature, precipitation, and presence of urban or crop lands for both scenarios (Fig. 3A). Under the SSP2-4.5 scenario, FOI was negatively associated with temperature seasonality and precipitation in the warmest quarter and positively associated with urban land cover (Fig. 3B). Under the SSP5-8.5 scenario, FOI was slightly negatively associated with precipitation of the wettest quarter and negatively associated with mean temperature of the coldest quarter, while urban land showed a positive association (Fig. 3B).

The models for investigating the changes in FOI versus features performed moderately well. The models under SSP 2-4.5 scenario for GTOV, JUNV and MACV explained 77.4%, 77.8%, 90.2% of variance, respectively (Pseudo R-squared estimate). Similarly, models under SSP 5-8.5 explained 75.2%, 72.4% and 87.5% of the variance in the changes in FOI, respectively.

### Predicted shifts in species distribution patterns of NWA rodent reservoirs

**Fig. 4:**
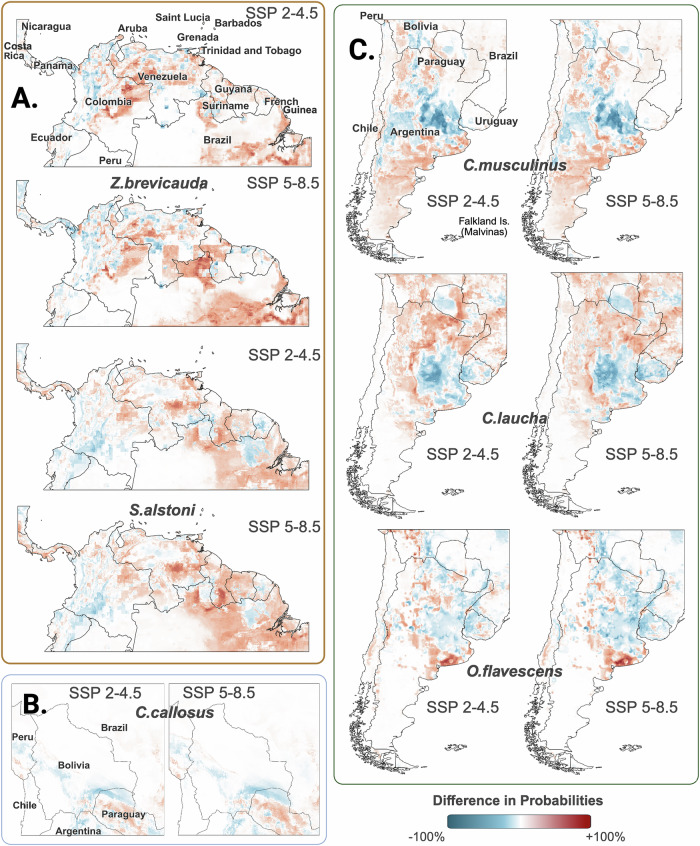
Change in the species distribution probabilities for six NWA rodent reservoir species between the current climate and the projected SSP 2-4.5 and the SSP 5-8.5 scenarios. **A** Change in species distribution probabilities for *Zygodontomys brevicauda* and *Sigmodon alstoni*, the reservoir species for Guanarito virus (GTOV), which causes Venezuelan Hemorrhagic fever (VHF). **B** Change in SDM probabilities for *Calomys callosus*, the reservoir species of Machupo virus (MACV), which causes Bolivian Hemorrhagic Fever (BHF). **C** Change in SDM probabilities for *Calomys musculinus*, *Calomys laucha* and *Oligoryzomys flavescens*, the reservoir species of Junin virus (JUNV), which causes Argentine Hemorrhagic Fever (AHF).

We predicted the species distribution patterns for the six rodent reservoirs of the three NWAs for (i) the current, and the future in years 2041–2060 represented by (ii) SSP 2-4.5 scenario and (iii) SSP 5-8.5 scenario, using a Species Distribution Modeling (SDM) framework. In general, the projected future species distributions showed different spatial patterns and magnitudes for presence probabilities for all rodent reservoir species compared to the current distributions, predicting a radical change in the habitats of these rodent reservoirs in the future. However, the differences in the probabilities of presence between the SSP 2–4.5 and SSP 5-8.5 scenarios, were more subtle (Fig. 4). We also predicted more widespread range of distribution for the NWA rodent reservoirs compared to their historically reported occurrences.

We projected the distribution of *Z. brevicauda* away from the Caribbean coast and towards forest and rural areas (Fig. 4A), with lower probabilities in existing metropolitan and highly populated areas. We also projected a westward shift of *S. alstoni* distribution for both SSP 2-4.5 and 5-8.5 scenarios. Our models did not predict similar shift patterns for the species distribution of *C. callosus*. We projected minor changes for *C. callosus*, with increased probability in northern parts of Paraguay and western Brazil, and lower probabilities in central Bolivia and northern Argentina (Fig. 4B). We predicted a decrease in distribution probabilities in the central region of Argentina for *C. musculinus* (Fig. 4C). Conversely, we predicted increased probabilities for *C. laucha* and *O. flavescens* in the same region, thereby showing changes in the distribution range of rodent species in this region (Fig. 4C). We projected the distribution of *O. flavescens* to shift northward, whereas that of *C. musculinus* to shift southward. The presence of *C. laucha* was projected to shift inland into the central region of Argentina. Detailed visual observations on maps of the geographical changes for each species distribution are shown in Supplementary Table 2.3.

The distribution of *Z. brevicauda* was mostly influenced by crop land and temperature seasonality. For *S. alstoni* the most important features (prediction drivers) were annual precipitation and temperature seasonality. For *C. callosus*, annual and diurnal ranges of temperature were the most important features. For *C. musculinus*, the most important features were presence of urban land, and annual precipitation. For *C. laucha*, crop land, maximum temperature in the warmest month and warmest quarter were the most important features. For *O. flavescens*, crop and urban land were equally important features followed by annual precipitation.

The fitted SDMs performed moderately well with a cross-validation (CV) accuracy between 77% and 87% and a test accuracy of 75% to 98% depending on the reservoir species (for details on precision, recall, F1 scores and confusion matrices, see Supplementary Information Section B 2.3).

## Discussion

Our models predicted that NWAs could expand into currently non-endemic areas as reservoir distributions shift under climate change, potentially increasing the risk of human spillover. Using an epidemiological perspective, we linked NWA rodent reservoir habitat patterns to potential human spillover risk. In our study, a higher FOI (proxy metric for the spillover risk) indicated a greater likelihood of reservoir presence and a greater likelihood of epidemiological contact between infectious reservoirs and humans. This approach differed from prior studies, which have used species distribution models for determining habitat suitability of disease vectors and reservoirs^22^.

In this study, the FOI was calculated under a density‑dependent framework for rodent–human transmission. We used a transmission rate parameter informed by Lassa fever (OWA) data, as a pragmatic proxy in the absence of NWA‑specific estimates. Although we lacked direct data on reservoir population densities, approximating rodent abundance from SDM‑derived occurrence probabilities offers a consistent, model‑based way to scale FOI across species and regions, while identifying population density as a key target for future field validation^23^. Thus, disease risk might be overestimated. Salazar et al. found that the geographic extent of rodent reservoir habitat was substantially larger than the areas where NWA outbreaks had been recorded^24^. However, it is highly probable that NWAs could spill over into non-endemic regions due to changes in human movement patterns, as well as changes and fragmentation of rodent habitats that may increase proximity to human settlements. This is in line with IPCC AR6 findings of increasing human-animal contact leading to the emergence of zoonotic spillovers, such as those of NWAs^25^.

Our model framework aimed to reduce cumulative uncertainty in future projections that systematically occurred due to modeling choices and assumptions. By fitting ensembles of SDMs and FOI models across iterative data resampling and parameterization, we summarized the spread of resulting risk instead of relying on a singular model outcome of changing spillover risks. This approach is consistent with recommendations for spatial uncertainty propagation and ensemble SDM modeling^26^. The uncertainty in FOI parameters and model structure was further reduced by the use of ensemble tree-based models with repeated cross-validation and recursive feature elimination, so that variation in fitted relationships between changes in the predictors and that of FOI was reflected in the distribution of out-of-sample predictions rather than a single deterministic surface (for details see “Methodological Discussion”).

Our outcomes conform with the findings of previous studies, which have shown that ecological traits of rodent reservoir species in the New World are sensitive to changes in temperature range and/or seasonality, as well as precipitation patterns and rainy season^27–29^. Secondary effects of climate change, such as alterations in anthropogenic land use and subsequent effects on disease dynamics have also been reported previously^30^. Considering that all six of the NWA reservoirs are highly adaptable species, habitat changes can be expected without significant loss in population size^31^. GTOV reservoirs (*Z. brevicauda*, *S. alstoni*) can be found in shrub and grassland areas with wet conditions. Hence, their sensitivity to seasonality in temperature and precipitation was expected^31^. In the case of MACV, we theorize that the apparent sensitivity to changes in temperature and precipitation might be ascribed to the habitat preferences of *C. callosus*, along with the expected increase in human migration towards non-urban areas, since *C. callosus* is known to inhabit areas with dry, semi-arid climate conditions with open vegetation^32,33^. Moreover, we predicted overlapping areas with high FOI of BHF and AHF, caused by MACV and JUNV, respectively. The persistence of potential hotspots in endemic regions is consistent with findings from Portuguesa State, Venezuela, where Silva-Ramos et al. documented increased and persistent hotspot zones for VHF outbreaks^34^.

Our findings are in line with prior studies showing that shifts in the habitat of rodent reservoirs of JUNV intersect with areas of human activity^6,35^. Crucially, we predicted widespread habitat distribution across all six rodent species that may signal a possible fragmentation of rodent habitats in the future. Particularly for MACV and JUNV, habitat fragmentation might be expected in the future in all climate change scenarios for Andean foothill biomes as well as grasslands in northern Argentina, Bolivia, and regions in neighboring Paraguay and Chile^36^. By predicting these fragmented habitats accurately, high-risk “bridge” areas between current endemic habitats could be identified to integrate into additional surveillance efforts.

Given that the current global climate trajectory aligns with the moderate climate change scenario of SSP 2-4.5^36,37^, our predictions may be particularly salient for monitoring potential outbreak hotspots and optimizing the use of scarce public health resources. Furthermore, our methodological framework for these diseases can be adapted to other neglected diseases, as well as other climate scenarios. The outcomes can serve as a template for global health agencies to prioritize at-risk communities and countries facing disproportionate health burdens due to outbreak risk, enabling proactive rather than reactive responses.

## Methods

### Rodent reservoirs for the New World Arenaviruses (NWA)

We selected six rodent species that are known to act as reservoirs for NWA and contribute to zoonotic spillover events (Table 1). The criterion for selection was that the reservoir species had to be seropositive for the virus during the time of the outbreak, with at least two studies confirming their reservoir status during the time of the outbreak^24,28,32,37–41^. The six rodent species, namely, *Zygodontomys brevicauda* and *Sigmodon alstoni* for GTOV, *Calomys callosus* for MACV, *Calomys musculinus, Calomys laucha*, and *Oligoryzomys flavescens* for JUNV, have been commonly associated with outbreaks of their respective NWA in human populations (Table 1)^42–46^.

**Table 1:** New World Arenaviruses (NWA) that have caused zoonotic outbreaks in humans and their reported rodent reservoirs

Common name of virus	Common name of the disease	Reported rodent reservoir	Time of outbreaks/active cases reported
Junin virus (JUNV)	Argentine Hemorrhagic fever	*Calomys musculinus* (Dryland Vesper mouse)	1950s, 1990–2018^42,43^
		*Calomys laucha* (Small Vesper mouse)	
		*Oligoryzomys flavescens* (Yellow pygmy rice rat)	
Machupo virus (MACV)	Bolivian Hemorrhagic fever	*Calomys callosus* (Large Vesper mouse)	1950s, 1970s^44^
Guanarito virus (GTOV)	Venezuelan Hemorrhagic fever	*Zygodontomys brevicauda* (Short-tailed Cane mouse)	1989–2010 ^45,46^
		*Sigmodon alstoni* (Alston’s Cotton rat)	

### Species Distribution Model (SDM)

**Fig. 5:**
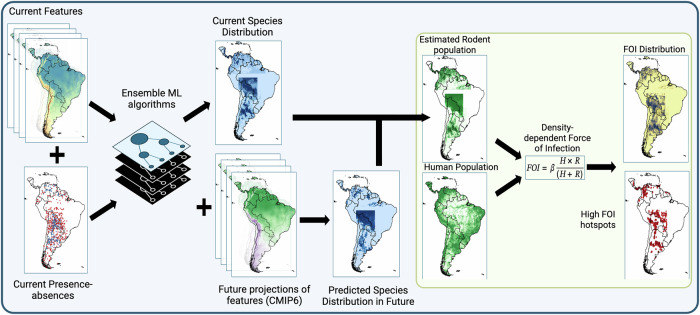
Schematic illustration of the framework for deriving the impact of climate change on the spillover risk for the three New World Arenaviruses (NWAs) in South America. ML Machine Learning, CMIP6 Coupled Model Intercomparison Project phase 6, FOI Force-of-Infection, H Human Population/unit area, R Rodent Population/unit area, beta transmission rate parameter.

Modeling frameworks such as species distribution models (SDMs), which can incorporate bioclimatic and environmental factors to estimate the distribution of rodent reservoirs, can be effectively employed in predicting transmission risk for associated diseases^47^. Since the modeled association between climate change and species distribution was not expected to be monotonic^48^ but rather complex and subtle, modifying the SDM framework to employ ensemble tree-based techniques such as random forest (rf), extra trees (et), extreme gradient boost (xgb), and light gradient boosting machine (lgbm) is warranted^49^. We followed a similar protocol to the one we previously used for modeling the species distribution of Calomys musculinus in Argentina^6^. The protocol was adapted to each of the rodent species selected for this study (Table 1). Here is a summary of the SDM protocol (Fig. 5) used to model the rodent reservoirs of NWA following the guidelines established for reporting Ecological Niche Models or SDMs to maximize reproducibility as closely as possible pertaining to our study^50^.

Occurrence data on all six rodent species were sourced from Global Biodiversity Information Facility (GBIF) database (presence-only)^51^ using R-package *dismo*^52^ (Derived datasets^53^). These data were cleaned to remove duplicates and restricted to years 1990 onward to match the period of climate/land use features. All occurrences in the South American continent were retained regardless of the country. Presence-only data were preprocessed to be converted to 1:1 presence-absence data using habitat suitability analysis with R-package *USE::paSampling()* utilizing a uniform approach^54^. This was done to avoid sample location bias that commonly occurs with random sampling of a geographical extent without considering the environmental conditions, as discussed by Da Re et al. (see Supplementary Section C3.4 for details)^55^. Environmental and ecological data were sourced from open-source datasets, such as 19 Bioclimatic variables from World Clim (Bioclim), Normalized Differential Vegetation Index (NDVI) and Digital Elevation Model (DEM) from Moderate-Resolution Imaging Spectroradiometer (MODIS) satellite database and five Land Use datasets from Land-Cover and Land-Use Change (LCLUC) program maintained jointly by NASA and University of Maryland^56–60^. These datasets were resampled to the highest common resolution and clipped to the extent of species occurrence data mentioned above for each individual rodent reservoir. In total, three sets of 24–26 rasters and six occurrence datasets were used for the SDMs (Supplementary Table 2.1).

Similarly, for predicting the species distribution in the future, analogous raster data for Bioclimactic variables^61^, NDVI (data from 2020 for NDVI^57^), DEM^58^, and land use^62^ were downloaded for Shared Socioeconomic Pathways 6 (SSP) scenarios of Moderate (SSP 2-4.5) and Extreme (SSP 5–8.5) predictions for climate change in the future as established by Coupled Model Intercomparison Project v6 (CMIP6) for years 2041 to 2060^21^. These datasets were also preprocessed and resampled to the highest common resolution and averaged over the 20-year time period.

SDMs were developed by fitting four ensemble tree-based classifier algorithms, namely, random forest (rf), extra trees (et), extreme gradient boost (xgb) and light gradient boosting machine (lgbm). The occurrence datasets for the six rodent species were considerably smaller compared to the standard size of presence-absence datasets in similar studies, limiting our capacity to perform spatial block cross-validation which is a standard practice for ecological models^63^. Instead, data was split randomly into train-test ratio of 4:1 (80% train; 20% test). The missing values of each predictor (raster features) from both the training and test sets were imputed using SimpleImputer() from scikit-learn python library^64^ with the mean of the existing values of each predictor. This was necessary for fitting “rf” and “et” algorithms to the training data. Each algorithm was trained with fivefold cross-validation and the predictions for training and the test set were generated to extract cross-validation accuracy, precision, recall and the F1 score based on (i) fivefold cross-validation and (ii) test set confusion matrices. This process was iterated 100 times to improve the robustness of the models using 100 different presence-absence resamples from the occurrence data. To retain the appropriate number of features necessary to predict the distribution probabilities, we used a recursive feature elimination (RFE) algorithm with stratified cross-validation using the RFECV algorithm^64^. Final model predictions were derived by averaging results across all iterations and the four classifier algorithms.

Since hyper-tuning did not yield significant improvements to any of the four classifier algorithms (details in Supplementary Section B: Hypertuning Results and Parameter Selection), the default hyperparameters were used. We expected a high level of collinearity between the rasters based on spatial correlation analysis (results not shown), given the nature of geospatial data. To counter this, RFE was performed at each iteration to improve the accuracy of the model without having potential issues with multicollinearity. In each iteration, 10 features were selected per classifier algorithm based on stratified cross validation of RFE performed before the iterative model training using *RFECV()* from *scikit-learn*^64^. Final fitted models were taken as the average of all the iterations and of the four classifier algorithms.

Based on the fitted models, the current distribution probabilities of each rodent species were imputed to the geographical extent of their occurrence data using pyimpute library in python^65^. Similarly, the future distributions of the rodents in response to SSP 2-4.5 and SSP 5-8.5 scenarios of CMIP6 climate change were imputed based on the fitted models. Changes in the distribution probabilities were mapped onto the raster for each of the scenarios by subtracting current probabilities from the future probabilities of species distribution. The process is illustrated in Fig. 5.

### Zoonotic risk for spillover and force of infection

Based on the distribution probabilities of the SDM for each rodent reservoir, a risk profile for human outbreak, i.e., zoonotic spillover of NWA, was modeled using infection dynamics simulation. The current and CMIP6 scenario-based projections of human population density were overlaid on the SDM probabilities to generate a risk profile based on the force of infection for NWA spillover. Force-of-infection (FOI) was calculated based on the contact rate between humans and the rodent reservoirs and the possibility of the rodent testing positive as reservoir for the NWA.

The mechanistic model used for estimating FOI was a density dependent contact rate model with binomial sampling as detailed in Eq. 1.

1 $${FOI}=\beta \frac{H\times R}{(H+R)}$$

Where, ${FOI}$ is the force of infection defined as the contact rate between susceptible humans and infectious rodents resulting in the successful transmission of infection, $\beta$ is the transmission rate parameter derived from review of analogous viral transmission dynamics studies (see Appendix: Force of Infection), $H$ is the population of susceptible humans set at 0.95 times that of total human population in the same geospatial coordinates of SDMs (author’s expertise and from study performed on Lassa Fever in Nigeria^66^) and $R$ is the infectious proportion of the rodent population based on the binomial sampling between 1 and 15 rodents per grid cell, adjusted with the probability of presence of rodents in the given geospatial coordinates based of the SDMs. Given the lack of data on rodent populations, we estimated the number of rodents present in each grid cell based on the estimation of rodent family size found in one location by Han et al.^67^ and binomial sampling. The denominator $\left(H+R\right)$ represents the total density of the interacting populations of human hosts and rodent reservoirs. Equation (1) was adapted from a generalized formula used in similar transmission studies of vector and rodent-borne infections with and without the effect of climate change^68–70^.

A similar methodology was followed using the projected SDM probabilities and the projected human population under the SSP 2-4.5 and SSP 5-8.5 scenarios of CMIP6^71^. For generating these maps, the FOI of each reservoir species of the same virus were combined ${FO}{I}_{{virus}}^{t}=\mathop{\sum }\nolimits_{r=1}^{n}{FO}{I}_{r}^{t}$ where r was the rodent reservoir and $t\epsilon \{{current},{SSP}2-4.5,{SSP}5-8.5\}$.

A similar process was done for ${FO}{I}^{{SSP}2-4.5}{andFO}{I}^{{SSP}5-8.5}$.

The differences ($\delta$) between the future climate change scenarios (SSP 2-4.5 and SSP 5-8.5) and the current scenario of FOI (${\delta }_{{foi}}^{{ssp}2-4.5}{and}{\delta }_{{foi}}^{{ssp}5.85}$) were calculated and scaled to be from −100% to + 100%.

The map depicting FOI for each of the three viruses under study was further reclassified in 10% quantiles and the geospatial zones that had FOI values in the top 10th percentile (≥90% of the FOI estimated range across the geographical bounds of the raster) were depicted as potential hotspot zones for outbreak of zoonotic arenaviruses.

### Association of changing risk with climate change

We modeled the associations between the changes in FOI and the changes in 24 bioclimatic and land-use features that were used to predict the species distribution patterns of the reservoir species. NDVI and DEM features were dropped from these analyses due to a high number of missing values. The difference (${\boldsymbol{\Delta }}$) between future climate change scenarios (SSP 2-4.5 and SSP 5-8.5) and the current scenario of FOI estimates (Eq. 2a), along with the corresponding features (Eq. 2b), were used as inputs in a random forest regressor model for each of the three viruses. The ${{\boldsymbol{\Delta }}}_{{\boldsymbol{f}}}^{{\boldsymbol{ssp}}}$ and ${{\boldsymbol{\Delta }}}_{{FOI}}^{{\boldsymbol{ssp}}}$ values were scaled and a combined tabular dataset was generated. A random forest regressor model was fitted (*RandomForestRegressor(), scikit-learn*^64^). For each random forest model, feature importance based on mean impurity reduction was extracted. The partial dependence plots of the top three most important features were developed from average grid values and predicted outcomes using functions from *scikit-learn, matplotlib* and *seaborn* packages in python^64,72,73^.

2a $${{\boldsymbol{\Delta }}}_{{FOI}}^{{\boldsymbol{ssp}}}\epsilon \{{\delta }_{{foi}}^{{ssp}\,2-4.5}{and}{\delta }_{{foi}}^{{ssp}\,5-8.5}\}$$

2b $${{{\boldsymbol{\Delta }}}_{{\boldsymbol{f}}}^{{\boldsymbol{ssp}}{\boldsymbol{2}}-{\boldsymbol{4}}{\boldsymbol{.}}{\boldsymbol{5}}}\epsilon \{\delta }_{{temp}\,{range}}^{{ssp}\,2-4.5},\,{\delta }_{{isothermality}}^{{ssp}\,2-4.5}\ldots {\delta }_{{crop}\,{land}}^{{ssp}\,2-4.5}\}\forall f$$

2c $${{\boldsymbol{\Delta }}}_{{\boldsymbol{f}}}^{{\boldsymbol{ssp}}{\boldsymbol{5}}-{\boldsymbol{8}}{\boldsymbol{.}}{\boldsymbol{5}}}\epsilon \{{\delta }_{{temp}\,{range}}^{{ssp}\,5-8.5},{\delta }_{{isothermality}}^{{ssp}\,5-8.5}\ldots {\delta }_{{crop}\,{land}}^{{ssp}\,5-8.5}\}\forall f$$

Where ${\boldsymbol{f}}$ are individual features in the dataset (for example, temperature range, annual precipitation, etc.)

### Methodology discussion

In our study, we restricted the focus to three NWAs—GTOV, MACV and JUNV—which have been associated with outbreaks of substantial magnitude^43^. This restriction excludes nine NWAs out of 19 identified mammarenaviruses^30^. Several of these NWAs have been shown to be emergent, such as Sabia virus in Brazil, Chapare virus in Bolivia, Pirital virus and Tacaribe virus in Venezuela and Guyana. However, only a few cases of these lesser-known NWAs have been reported with confirmed diagnoses or acute deaths investigated^43^. Of note, rodent species that are reservoirs for one virus may also be reservoirs for other similarly transmitted viruses and are thus non-specific reservoirs. For example, reservoirs of VHF caused by Guanarito virus have been shown to carry viruses from the Bunyaviridae family, as well as other hantaviruses^74^. The issue of putative rodent reservoirs is further complicated by the contentious classification of rodent species in the New World. The continuously expanding species diversity and evolving classification of these rodents (especially around the Andes foothills^75^), combined with different nomenclature and local names, complicates the study and targeting of NWAs reservoir species. We thus applied a species distribution model for rodent reservoirs that was not limited to the extent of historically endemic zones of NWAs, but rather by the geographical extent of reported occurrences of the rodents. A macro-ecological outlook that is reservoir-centric rather than disease outbreak-centric could be more helpful, given the lack of clarity on reservoir species diversity and their classification^67^. Whether multiple reservoirs exist for the same virus, a single reservoir carries multiple viruses, or multiple reservoirs carry multiple viruses, the risk NWA zoonotic spillover heavily depends on the presence, density and habitat patterns of reservoir hosts^67,76^.

To investigate the association between changes in climate and changes in the spillover risk represented by FOI, we fitted a random forest (RF) model. The changes in bioclimatic and land use variables (${{\boldsymbol{\Delta }}}_{{\boldsymbol{f}}}^{{\boldsymbol{ssp}}}$) were multi-collinear given the nature of the SSP scenario estimation of bioclimatic (see ref. ^61^ for details) and land use variables. Tree-based ensemble models such as RF are more robust to multicollinearity than classical statistical models, which are normally applied in association studies when iterated multiple times and combined to give a single estimate of permutation feature importance^77^. These ensembles naturally capture non-monotonic effects and higher‑order interactions among climate variables, which are expected in ecological and epidemiological responses to climate change^78^. While our outcomes cannot demonstrate statistically significant associations between changes in features and changes in FOI, the RF model identified feature importances based on permutation importances over 100 model fits each (3 viruses × 2 SSP scenarios × 100 iterations of models = 600 model fits). These feature importance results indicated associated trends in change between climate and FOI.

Similar to the random forest models used to investigate associations between FOI and climate change, we developed and implemented SDMs using tree‑based ensemble algorithms. This method is more robust to multicollinearity among predictors and can accommodate nonlinear responses and interactions without relying on unstable coefficient estimates, compared to classical statistical methods^79^. To address the multicollinearity of bioclimatic and land‑use covariates (details in Supplementary Section C), we combined repeated model refitting on resampled presence–absence data with recursive feature elimination embedded in cross‑validation, thereby selecting a subset of predictors based on out‑of‑sample predictive performance rather than manually defined arbitrary correlation thresholds^80,81^. In line with the concerns raised by Dormann et al. that collinearity is pervasive in ecological data and that no single technique fully resolves its effects^82^. Permutation importance per feature was then calculated repeatedly on the RFE‑selected ensembles to quantify each predictor’s contribution under the observed collinearity structure (including standard errors), providing an interpretable ranking of climate and land‑use drivers of projected changes in disease risk (Supplementary Fig. 2.3). By combining these machine-driven techniques, we let out‑of‑sample performance determine which correlated climate predictors were retained, rather than relying on fixed correlation coefficient cut‑offs.

## Supplementary information

Supplementary Information

## Data Availability

Modified historical occurrences from the GBIF database on all six rodent species are available at 10.15468/DD.6U8HUK. Spatial datasets utilized in this study are all sourced from open source web platforms such as World Clim (Bioclim), Normalized Differential Vegetation Index (NDVI) and Digital Elevation Model (DEM) from Moderate-Resolution Imaging Spectroradiometer (MODIS) satellite database and from Land-Cover and Land-Use Change (LCLUC) program maintained jointly by NASA and University of Maryland and cited accordingly in the manuscript.

## Code availability

All generated and modified code pertaining to this manuscript is available on 10.5281/zenodo.17137588 and https://github.com/EpiPandit/Kulkarni_et_al_Arenaviruses.

## References

[1] Rupasinghe, R., Chomel, B. B. & Martínez-López, B. Climate change and zoonoses: a review of the current status, knowledge gaps, and future trends. *Acta Trop.* **226**, 106225 (2022). 34758355 10.1016/j.actatropica.2021.106225

[2] El-Sayed, A. & Kamel, M. Climatic changes and their role in emergence and re-emergence of diseases. *Environ. Sci. Pollut. Res.* **27**, 22336–22352 (2020). 10.1007/s11356-020-08896-w PMC7187803 32347486

[3] Dahmana, H. et al. Rodents as hosts of pathogens and related zoonotic disease risk. *Pathogens* **9**, 202 (2020). 32164206 10.3390/pathogens9030202 PMC7157691

[4] Carlson, C. J. et al. Climate change increases cross-species viral transmission risk. *Nature* **607**, 555–562 (2022). 35483403 10.1038/s41586-022-04788-w

[5] Clegg, J. C. Influence of climate change on the incidence and impact of arenavirus diseases: a speculative assessment. *Clin. Microbiol. Infect.* **15**, 504–509 (2009). 19604274 10.1111/j.1469-0691.2009.02847.x

[6] Flores-Pérez, N., Kulkarni, P., Uhart, M. & Pandit, P. S. Climate change impact on human-rodent interfaces: modeling junin virus reservoir shifts. *EcoHealth*. 10.1007/s10393-025-01723-z (2025). 10.1007/s10393-025-01723-z PMC12476445 40576885

[7] WHO EMRO. Zoonotic disease: emerging public health threats in the region. World Health Organization - Regional Office for the Eastern Mediterranean. http://www.emro.who.int/fr/about-who/rc61/zoonotic-diseases.html.

[8] Redding, D. W. et al. Geographical drivers and climate-linked dynamics of Lassa fever in Nigeria. *Nat. Commun.* **12**, 5759 (2021). 34599162 10.1038/s41467-021-25910-y PMC8486829

[9] Guterres, A. & de Lemos, E. R. S. Hantaviruses and a neglected environmental determinant. *One Health* **5**, 27–33 (2018). 29911161 10.1016/j.onehlt.2017.12.002 PMC6000911

[10] CDC. About Viral Hemorrhagic Fevers. Viral Hemorrhagic Fevers (VHFs) https://www.cdc.gov/viral-hemorrhagic-fevers/about/index.html (2024).

[11] Silva-Ramos, C. R., Montoya-Ruíz, C., Faccini-Martínez, ÁA. & Rodas, J. D. An updated review and current challenges of Guanarito virus infection, Venezuelan hemorrhagic fever. *Arch. Virol.* **167**, 1727–1738 (2022). 35579715 10.1007/s00705-022-05453-3 PMC9110938

[12] Sarute, N. & Ross, S. R. New World Arenavirus biology. *Annu. Rev. Virol.* **4**, 141–158 (2017). 28645238 10.1146/annurev-virology-101416-042001 PMC7478856

[13] Gómez, R. M. et al. Junín virus. A XXI century update. *Microbes Infect* **13**, 303–311 (2011). 21238601 10.1016/j.micinf.2010.12.006

[14] NIAID. NIAID Biodefense Pathogens | NIAID: National Institute of Allergy and Infectious Diseases. https://www.niaid.nih.gov/research/niaid-biodefense-pathogens (2024).

[15] WHO EMRO. Prioritizing diseases for research and development in emergency contexts. https://www.who.int/activities/prioritizing-diseases-for-research-and-development-in-emergency-contexts.

[16] Doohan, P. et al. Lassa fever outbreaks, mathematical models, and disease parameters: a systematic review and meta-analysis. *Lancet Glob. Health* **12**, e1962–e1972 (2024). 39577970 10.1016/S2214-109X(24)00379-6

[17] Basinski, A. J. et al. Bridging the gap: Using reservoir ecology and human serosurveys to estimate Lassa virus spillover in West Africa. *PLOS Comput. Biol.* **17**, e1008811 (2021). 33657095 10.1371/journal.pcbi.1008811 PMC7959400

[18] Ogundele, G. O., Jolayemi, K. O. & Bello, S. Lassa fever in West Africa: a systematic review and meta-analysis of attack rates, case fatality rates and risk factors. *BMC Public Health* **25**, 2948 (2025). 40866826 10.1186/s12889-025-24377-6 PMC12382145

[19] Birch, E. L. A Review of “Climate Change 2014: Impacts, Adaptation, and Vulnerability” and “Climate Change 2014: Mitigation of Climate Change”: Intergovernmental Panel on Climate Change. (2014). (Contribution of Working Group II to the Fifth Assessment Report of the Intergovernmental Panel on Climate Change). New York, NY: Cambridge University Press. 2621 pages. Available online at http://ipcc-wg2.gov/AR5/report/final-drafts/; Intergovernmental Panel on Climate Change. (2014). (Contribution of Working Group III to the Fifth Assessment Report of the Intergovernmental Panel on Climate Change). New York, NY: Cambridge University Press. 1,967 pages. Available online at https://www.ipcc.ch/report/ar5/wg3/. *J. Am. Plann. Assoc.* **80**, 184–185 (2014).

[20] Hagen, I. et al. Climate change-related risks and adaptation potential in Central and South America during the 21st century. *Environ. Res. Lett.* **17**, 033002 (2022).

[21] Masson-Delmotte, V. et al. Summary for policymakers. In: Climate Change 2021: The Physical Science Basis. Contribution of Working Group I to the Sixth Assessment Report of the Intergovernmental Panel on Climate Change. https://www.ipcc.ch/report/ar6/wg1/downloads/report/IPCC_AR6_WGI_SPM_final.pdf (2021).

[22] Kopsco, H. L., Smith, R. L. & Halsey, S. J. A scoping review of species distribution modeling methods for tick vectors. *Front. Ecol. Evol.* **10**, 893016 (2022).

[23] Oliver, T. H. et al. Population density but not stability can be predicted from species distribution models. *J. Appl. Ecol.* **49**, 581–590 (2012).

[24] Salazar-Bravo, J. et al. Natural nidality in Bolivian hemorrhagic fever and the systematics of the reservoir species. *Infect. Genet. Evol.* **1**, 191–199 (2002). 12798015 10.1016/s1567-1348(02)00026-6

[25] Intergovernmental Panel On Climate Change (IPCC). Climate Change 2022 – Impacts, Adaptation and Vulnerability: Working Group II Contribution to the Sixth Assessment Report of the Intergovernmental Panel on Climate Change. 10.1017/9781009325844 (Cambridge University Press, 2023)

[26] Thuiller, W., Guéguen, M., Renaud, J., Karger, D. N. & Zimmermann, N. E. Uncertainty in ensembles of global biodiversity scenarios. *Nat. Commun.* **10**, 1446 (2019). 30926936 10.1038/s41467-019-09519-w PMC6441032

[27] Mills, J. N. & Childs, J. E. Ecologic studies of rodent reservoirs: their relevance for human health. *Emerg. Infect. Dis. J.* **4**, 10.3201/eid0404.980403 (1998). 10.3201/eid0404.980403 PMC2640244 9866729

[28] Mills, J. N. et al. A longitudinal study of Junin virus activity in the rodent reservoir of Agrentine hemorrhagic fever. *Am. J. Trop. Med. Hyg.* **47**, 749–763 (1992). 1335214 10.4269/ajtmh.1992.47.749

[29] Polop, F. et al. On the relationship between the environmental history and the epidemiological situation of Argentine hemorrhagic fever. *Ecol. Res.* **23**, 217–225 (2008).

[30] Tapia-Ramírez, G. et al. A review of mammarenaviruses and rodent reservoirs in the Americas. *EcoHealth* **19**, 22–39 (2022). 35247117 10.1007/s10393-022-01580-0 PMC9090702

[31] Lord, R. D. *Mammals of South America* (JHU Press, 2007).

[32] Salazar-Bravo, J., Ruedas, L. A. & Yates, T. L. Mammalian reservoirs of Arenaviruses. In (ed. Oldstone, M.B.A.) *Arenaviruses I: The Epidemiology, Molecular and Cell Biology of Arenaviruses* 25–63. 10.1007/978-3-642-56029-3_2 (Springer, 2002). 10.1007/978-3-642-56029-3_2 11987807

[33] Salazar-Bravo, Jorge, Salazar-Bravo, J. & University, T. T. *Revised Checklist of Bolivian Mammals*. 10.5962/bhl.title.156816 (Museum of Texas Tech University, 2003).

[34] Silva-Ramos, C. R. et al. Endemic Arenaviruses in Latin America. In *Emerging Viruses in Latin America: Contemporary Virology* (eds Pujol, F. H. & Paniz-Mondolfi, A. E.) 85–137. 10.1007/978-3-031-68419-7_4 (Springer Nature Switzerland, 2024).

[35] Porcasi, X. et al. Predictive distribution maps of rodent reservoir species of zoonoses in Southern America. *Mastozool. Neotropical* **12**, 199–216 (2005).

[36] Delgado, R. C., De Santana, R. O., Gelsleichter, Y. A. & Pereira, M. G. Degradation of South American biomes: what to expect for the future? *Environ. Impact Assess. Rev.* **96**, 106815 (2022).

[37] Milazzo, M. L. et al. Transmission of Guanarito and pirital viruses among wild rodents, Venezuela. *Emerg Infect Dis J*. **17**10.3201/eid1712.110393 (2011). 10.3201/eid1712.110393 PMC3311192 22172205

[38] Tesh, R. B. et al. Field studies on the epidemiology of Venezuelan hemorrhagic fever: implication of the cotton rat Sigmodon alstoni as the probable rodent reservoir. *Am. J. Trop. Med. Hyg.* **49**, 227–235 (1993). 8395143 10.4269/ajtmh.1993.49.227

[39] Weaver, S. C. et al. Guanarito virus (Arenaviridae) isolates from endemic and outlying localities in Venezuela: sequence comparisons among and within strains isolated from Venezuelan hemorrhagic fever patients and rodents. *Virology* **266**, 189–195 (2000). 10612673 10.1006/viro.1999.0067

[40] Morales, M. A. et al. Evaluation of an enzyme-linked immunosorbent assay for detection of antibodies to Junin virus in rodents. *J. Virol. Methods* **103**, 57–66 (2002). 11906733 10.1016/s0166-0934(01)00452-9

[41] González-Ittig, R. E. et al. Molecular systematics and biogeographic insights of the Calomys callosus complex (Rodentia, Cricetidae). *Zool. Scr.* **51**, 498–521 (2022).

[42] Piacenza, M. F. et al. Spatial difference in the incidence of Argentine Hemorrhagic Fever and composition and abundance of rodents in the assemblage. *Rev. Chil. Infectol.* **35**, 386–394 (2018). 10.4067/s0716-10182018000400386 30534925

[43] Lendino, A., Castellanos, A. A., Pigott, D. M. & Han, B. A. A review of emerging health threats from zoonotic New World mammarenaviruses. *BMC Microbiol.* **24**, 115 (2024). 38575867 10.1186/s12866-024-03257-w PMC10993514

[44] Patterson, M., Grant, A. & Paessler, S. Epidemiology and pathogenesis of Bolivian hemorrhagic fever. *Curr. Opin. Virol.* **5**, 82–90 (2014). 24636947 10.1016/j.coviro.2014.02.007 PMC4028408

[45] Manzione, N. et al. Venezuelan hemorrhagic fever: clinical and epidemiological studies of 165 cases. *Clin. Infect. Dis.* **26**, 308–313 (1998). 9502447 10.1086/516299

[46] Salas, R. et al. Venezuelan hemorrhagic fever. *Lancet* **338**, 1033–1036 (1991). 1681354 10.1016/0140-6736(91)91899-6

[47] Elith, J. & Leathwick, J. R. Species distribution models: ecological explanation and prediction across space and time. *Annu. Rev. Ecol. Evol. Syst.* **40**, 677–697 (2009).

[48] Ebi, K. L., Ziska, L. H. & Yohe, G. W. The shape of impacts to come: lessons and opportunities for adaptation from uneven increases in global and regional temperatures. *Clim. Change* **139**, 341–349 (2016).

[49] Valavi, R. et al. Predictive performance of presence-only species distribution models: a benchmark study with reproducible code. *Ecol. Monogr*. **92**, e01486. https://esajournals.onlinelibrary.wiley.com/doi/full/10.1002/ecm.1486 (2022).

[50] Feng, X. et al. A checklist for maximizing reproducibility of ecological niche models. *Nat. Ecol. Evol.* **3**, 1382–1395 (2019). 31548646 10.1038/s41559-019-0972-5

[51] GBIF. What is GBIF? https://www.gbif.org/what-is-gbi.

[52] Hijmans, R. J., Phillips, S., Leathwick, J. & Elith, J. *dismo: Species Distribution Modeling*. 1.3-16 10.32614/CRAN.package.dismo (2010).

[53] Pranav S.Kulkarni (uid: vetpsk). Derived dataset for Kulkarni et al. The Global Biodiversity Information Facility. 10.15468/DD.6U8HUK (2025).

[54] Re, D.D. et al. Use it: Uniformly sampling pseudo-absences within the environmental space for applications in habitat suitability models. *Methods Ecol. Evol.* **14**, 2873–2887. https://besjournals.onlinelibrary.wiley.com/doi/full/10.1111/2041-210X.14209 (2023).

[55] Da Re, D. et al. USE it: Uniformly sampling pseudo-absences within the environmental space for applications in habitat suitability models. *Methods Ecol. Evol.* **14**, 2873–2887 (2023).

[56] Hijmans, R. J., Cameron, S. E., Parra, J. L., Jones, P. G. & Jarvis, A. Very high resolution interpolated climate surfaces for global land areas. *Int. J. Climatol. J. R. Meteorol. Soc.* **25**, 1965–1978 (2005).

[57] Didan, K. MODIS/Terra Vegetation Indices 16-Day L3 Global 500m SIN Grid V061. NASA EOSDIS Land Processes Distributed Active Archive Center. 10.5067/MODIS/MOD13A1.061 (2021).

[58] Earth Resources Observation And Science (EROS) Center. Shuttle Radar Topography Mission (SRTM) 1 Arc-Second Global. 10.5066/F7PR7TFT (U.S. Geological Survey, 2017).

[59] Potapov, P. et al. The global 2000-2020 land cover and land use change dataset derived from the Landsat archive: first results. *Front. Remote Sens.* **3**, 856903 (2022).

[60] Booth, T. H., Nix, H. A., Busby, J. R. & Hutchinson, M. F. bioclim: the first species distribution modelling package, its early applications and relevance to most current MaxEnt studies. *Divers. Distrib.* **20**, 1–9 (2014).

[61] Boucher, O. et al. Presentation and evaluation of the IPSL-CM6A-LR climate model. *J. Adv. Model. Earth Syst.* **12**, e2019MS002010. https://agupubs.onlinelibrary.wiley.com/doi/full/10.1029/2019MS002010 (2020).

[62] Hurtt, G. et al. Harmonization of global land use change and management for the period 2015–2300. Earth System Grid Federation 10.22033/ESGF/INPUT4MIPS.10468 (2019).

[63] Roberts, D. R. et al. Cross-validation strategies for data with temporal, spatial, hierarchical, or phylogenetic structure. *Ecography* **40**, 913–929 (2017).

[64] Pedregosa, F. et al. Scikit-learn: Machine Learning in Python. Mach. Learn. PYTHON.

[65] Matthew Perry. pyimpute: Utilities for applying scikit-learn to spatial datasets (2022).

[66] Barua, S., Dénes, A. & Ibrahim, M. A. A seasonal model to assess intervention strategies for preventing periodic recurrence of Lassa fever. *Heliyon* **7**, e07760 (2021). 10.1016/j.heliyon.2021.e07760 PMC8367792 34430743

[67] Han, B. A., O’Regan, S. M., Paul Schmidt, J. & Drake, J. M. Integrating data mining and transmission theory in the ecology of infectious diseases. *Ecol. Lett.* **23**, 1178–1188 (2020). 32441459 10.1111/ele.13520 PMC7384120

[68] Begon, M. et al. A clarification of transmission terms in host-microparasite models: numbers, densities and areas. *Epidemiol. Infect.* **129**, 147–153 (2002). 12211582 10.1017/s0950268802007148 PMC2869860

[69] Medone, P., Ceccarelli, S., Parham, P. E., Figuera, A. & Rabinovich, J. E. The impact of climate change on the geographical distribution of two vectors of Chagas disease: implications for the force of infection. *Philos. Trans. R. Soc. B Biol. Sci.* **370**, 20130560 (2015). 10.1098/rstb.2013.0560 PMC4342964 25688019

[70] Ferrari, M. J., Perkins, S. E., Pomeroy, L. W. & Bjørnstad, O. N. Pathogens, social networks, and the paradox of transmission scaling. *Interdiscip. Perspect. Infect. Dis.* **2011**, 267049 (2011). 21436998 10.1155/2011/267049 PMC3062980

[71] Jones, B. & O’Neill, B. C. Spatially explicit global population scenarios consistent with the Shared Socioeconomic Pathways. *Environ. Res. Lett.* **11**, 084003 (2016).

[72] Waskom, M. seaborn: statistical data visualization. *J. Open Source Softw.* **6**, 3021 (2021).

[73] Hunter, J. D. Matplotlib: a 2D graphics environment. *Comput. Sci. Eng.* **9**, 90–95 (2007).

[74] Rodriguez-Morales, A. J., Castañeda-Hernández, D. M., Escalera-Antezana, J. P., Alvarado-Arnez, L. E. Organisms of concern but not foodborne or confirmed foodborne: Bolivian Hemorrhagic Fever Virus (Machupo Virus)☆. *Reference Module in Food Science*. 10.1016/B978-0-08-100596-5.22639-5 (Elsevier, 2019).

[75] Vallejos-Garrido, P. et al. The importance of the Andes in the evolutionary radiation of Sigmodontinae (Rodentia, Cricetidae), the most diverse group of mammals in the Neotropics. *Sci. Rep.* **13**, 2207 (2023). 36750620 10.1038/s41598-023-28497-0 PMC9905555

[76] Dobson, A. Population dynamics of pathogens with multiple host species. *Am. Nat.* **164**, S64–S78 (2004). 15540143 10.1086/424681

[77] Grebovic, M., Filipovic, L., Katnic, I., Vukotic, M. & Popovic, T. Machine learning models for statistical analysis. *Int. Arab J. Inf. Technol.* **20**, (2023).

[78] Swaminathan, R., Quaife, T. & Allan, R. A machine learning framework to evaluate vegetation modeling in Earth system models. *J. Adv. Model. Earth Syst.* **16**, e2023MS004097 (2024).

[79] Hanberry, B. B. Practical guide for retaining correlated climate variables and unthinned samples in species distribution modeling, using random forests. *Ecol. Inform.* **79**, 102406 (2024).

[80] Recursive Feature Elimination — Yellowbrick v1.5 documentation. https://www.scikit-yb.org/en/latest/api/model_selection/rfecv.html.

[81] RFECV. scikit-learn Machine Learning in Python. https://www.scikit-learn/stable/modules/generated/sklearn.feature_selection.RFECV.html.

[82] Dormann, C. F. et al. Collinearity: a review of methods to deal with it and a simulation study evaluating their performance. *Ecography* **36**, 27–46 (2013).

