# Supplementary material for: Climate-driven changes in zoonotic risk of arenaviral hemorrhagic fevers in South America
Source: Npj Viruses. 2026 Apr 15;4:23. doi: 10.1038/s44298-026-00189-2 (PMC13083987; doi:10.1038/s44298-026-00189-2)
Supplement: Supplementary file 1 — Supplementary Information [file 44298_2026_189_MOESM1_ESM.pdf]

Supplementary Information

Climate-driven changes in zoonotic risk of arenaviral hemorrhagic fevers in South America

Pranav S. Kulkarni [1], Nuri Y. Flores-Perez [1], Marcela Uhart [2,3], Brian Bird [2], Christine K. Johnson [2], Pranav S. Pandit [1].

[1] Department of Population Health and Reproduction, School of Veterinary Medicine, University of California, Davis

[2] One Health Institute, School of Veterinary Medicine, University of California, Davis

[3] Karen C. Drayer Wildlife Center, School of Veterinary Medicine, University of California, Davis

PS. Kulkarni, NY. Flores-Perez, M. Uhart, BH. Bird, CK. Johnson, PS. Pandit.

Section A. Force of infection for New World Arenaviruses (NWA) in South America

1.1 Deriving the model for Force of infection

The impact of climate change on the transmission risks for humans was studied by deriving the estimated rate of successful contact between susceptible humans and infectious rodents that resulted in transmission. This was termed as density dependent Force Of Infection (FOI). The exact mechanistic model for deriving the FOI for each of the geospatial coordinates and its connection with Species Distribution Models (SDMs) of rodent reservoirs of NWA is given by Equation 1:

$$FOI = \beta \frac{H \times R}{(H+R)} \qquad \text{Eq. 1.1}$$

Where, FOI is the force of infection defined as the contact between susceptible humans and infectious rodents resulting in successful transmission of infection, H is the population of susceptible humans set at 0.95 times that of total human population in the same geospatial coordinates of SDMs (author's expertise and from study performed on Lassa Fever in Nigeria [1]) and R is the proportion of infectious rodent population based on the binomial sampling between 1 and 15 rodents per grid cell, adjusted with the probability of presence of rodents in the given geospatial coordinates based of the SDMs. The denominator $(H + R)$ represents the total density of the interacting populations of human hosts and rodent reservoirs. β is the transmission rate parameter derived from review of analogous viral transmission dynamics studies as seen in Table 1.1. Two extreme average value of β or contact rate per week were tested, namely $\beta = 0.00286$ and $\beta = 0.0227$. Of these two, since the FOI scaled linearly due to density dependence ($FOI \propto \beta$) with change in β, the highest value of 2% ($\beta = 0.0227$) was selected for deriving the hotspots for potential outbreaks. R was the proportion of infectious rodents which was taken to be 0.645, adapted from study on Argentine Hemorrhagic Fever (AHF) caused by Junin virus from reservoir *Calomys musculinus* which estimated a prevalence rate of seropositive rodents between 0.2% to 10.9% [2]. Same infectious rate of rodents was applied to Guanarito virus and Machupo virus and their respective rodent reservoirs as well as to the other two reservoirs of Junin virus since no studies were found estimating the prevalence to date. Given these assumed inputs, the number of susceptible humans and infectious rodents in each geospatial coordinate grid cell was given by Equations 1.2 and 1.3:

$$H = h \times pS = 0.95h \qquad \text{Eq. 1.2}$$

$$R = pI \times r = 0.645r, \; r \sim Binom(15, N, p = pSDM) \;\; pI = 0.645 \qquad \text{Eq. 1.3}$$

Where, h is the human population in each geospatial grid cell of the SDM map, pS is the proportion of susceptible humans from h, r is the estimated number of rodents in N grid cell sampled from binomial distribution with average number of rodents assumed to be 15 modulated by the probability of presence of rodents ($pSDM$) and pI was the proportion of rodents that were assumed to be infectious.

Supplementary Material: Climate-driven changes in zoonotic risk of arenaviral hemorrhagic fevers in South America

PS. Kulkarni, NY. Flores-Perez, M. Uhart, BH. Bird, CK. Johnson, PS. Pandit.

Table 1.1 (A) Source literature used to determine the transmission rate parameter for Force Of Infection (FOI) estimation

Value [95% CI]	Subcategory	Transmission Route (unit)	Virus	Region	Estimated/ Derived	Year	Value Description
0.0024 [3]	Peridomestic areas	Deer mouse (*Peromyscus maniculatus*) to deer mouse (per month)	Hantavirus	Montana, United States	Derived	2009	Direct transmission rate from an infected mouse to a susceptible one. Derived from the direct transmission rates that produced a prevalence of 15% based on the study's model. Rates were different between peridomestic and sylvan areas studied.
0.0052 [3]	Sylvan areas						
0.43 [.38-.48] [4]	--	Rat (*Mastomys natalensis*) to rat (per month)	Lassa virus	Guinea and Mali	Derived	2014	Infection rate. LASV RNA and antibody prevalences among captured rodents were combined to estimate the overall infection rate.
0.8 [5]	--	Rat (*Mastomys natalensis)* to human (per day)	Lassa virus	--	Estimated	2015	Infection rate of humans from contact with rat excreta.
0.6 [6]	--	Rat (*Mastomys natalensis*) to human (no unit described)	Lassa virus	--	Estimated	2017	Effective transmission rate in susceptible humans by infected rodents.
0.43 [4,7]	--	Rat (*Mastomys natalensis*) to human (no unit described)	Lassa virus	--	Estimated<br>Estimated	2018	Transmission rate from infected rodents to susceptible humans.
0.0024 [3,8]	--	Deer mouse (*Peromyscus maniculatus*) to deer mouse (per month)	Hantavirus	--	Estimated<br>Estimated	2019	Direct transmission rate from an infected mouse to a susceptible one.
1.0 [0.1-2.6] [9]	January to April (low-density, no-breeding period)	Rat (*Mastomys natalensis*) to rat (per day)	Mongoro virus	Tanzania	Derived	2019	Transmission coefficent. Derived from the best fit model assuming no horizontal transmission with 1% chronic
4.7 [2.8-7.2] [9]	April to July (low-density,						

2.7 [1.4-3.9] [9]	breeding period) August to December (high-density, no-breeding period)						carriers. Seasonality influenced the value.
0.024 to 0.048 [10,11]	--	Rat (*Mastomys natalensis*) to human (no unit described)	Lassa virus	--	Estimated<br>Estimated	2020	Transmission rate from an infected rat to a susceptible human. Value taken from a study looking at the probability of transmission of *P. falciparum* from recovered humans to susceptible mosquitoes in areas of low and high transmission.
0.075 [12]<br>0.03 to 0.2 [12]	Baseline<br>Range	Rat (*Mastomys natalensis*) to human (per day)	Lassa virus	Nigeria	Estimated	2020	Transmission rate from rats to humans.
0.00001 [6,13]	--	Rat (*Mastomys natalensis*) to human (per day)	Lassa virus	Nigeria	Estimated (Obabiyi and Onifade, 2017)	2020	Contact rate between susceptible humans and infected rats.
0.0182 [14,15]	--	Rat (*Mastomys natalensis*) to human (per day)	Lassa virus	Nigeria	Estimated<br>Estimated	2020	Probability of transmission per contact by an infectious rat. Value taken from a study looking at Typhoid fever transmission dynamics among humans.
1.9 to 2.9 [16]	--	Rat (*Mastomys natalensis*) to human (per day per susceptible human)	Lassa virus	West Africa	Derived	2021	Rate of new infections. Describes the force-of-infection multiplied by the number of susceptible people. The range of rates covers the individual country rates derived in the study.
0.0296 [17]<br>0.1 to 0.8 [17]<br>0.216 [12,17]	Baseline<br>Range<br>Rate associated with disease extinction	Rat (*Mastomys natalensis*) to human (per day)	Lassa virus	Nigeria	Derived<br>Estimated	2021	Transmission rate from rodent to human. Derived from model-fitting to confirmed cases of Lassa fever in

0.373 [12,17]	Rate associated with disease persistence						Nigeria from 2017 to 2020 .
0.0179 [18]	2018	Rat (*Mastomys natalensis*) to human or rat (per week)	Lassa virus	Nigeria	Derived	2021	Transmission probability from rodents to humans and rodents. Derived from model-fitting to weekly reported cases of Lassa fever in Nigeria from 2018 to 2020.
0.0627 [18]	2019						
0.0553 [18]	2020						
0.0372 [18]	Estimated mean						
0.43 [7,19]	--	Rat (*Mastomys natalensis*) to human (per day)	Lassa virus	Nigeria	Estimated	2021	Contact rate of infectious rats to humans.
0.5 [20]	--	Human to human (per day)	Lassa virus	--	Estimated	2021	Transmission rate resulting from interaction between a susceptible human and an active virus reservoir.
0.0372 [18,21]	--	Rat (*Mastomys natalensis*) to human (per day)	Lassa virus	--	Estimated	2022	Rat-to-human effective transmission rate.
1.765x10^-11 [21]	Female	Rat (*Mastomys natalensis*) to human (per week)	Lassa virus	Nigeria	Derived	2024	Rate of infection in female or male humans via rodents. Derived by fitting a model to data on female and male Lassa fever cases collected from the Nigeria Centre for Disease Control and Prevention database beginning in January 2020 and spanning 65 weeks.
2x10^-11 [21]	Male						

1.2 Hotspots for potential outbreak

Potential hotspots for spillover risk were defined as 90th percentile (top 10%) zones of FOI estimates. The marked increase in number of potential hotspots corresponded to changing species distribution patterns of rodent reservoirs combined with changes in human population density in each hotspot area.

Supplementary Material: Climate-driven changes in zoonotic risk of arenaviral hemorrhagic fevers in South America

PS. Kulkarni, NY. Flores-Perez, M. Uhart, BH. Bird, CK. Johnson, PS. Pandit.

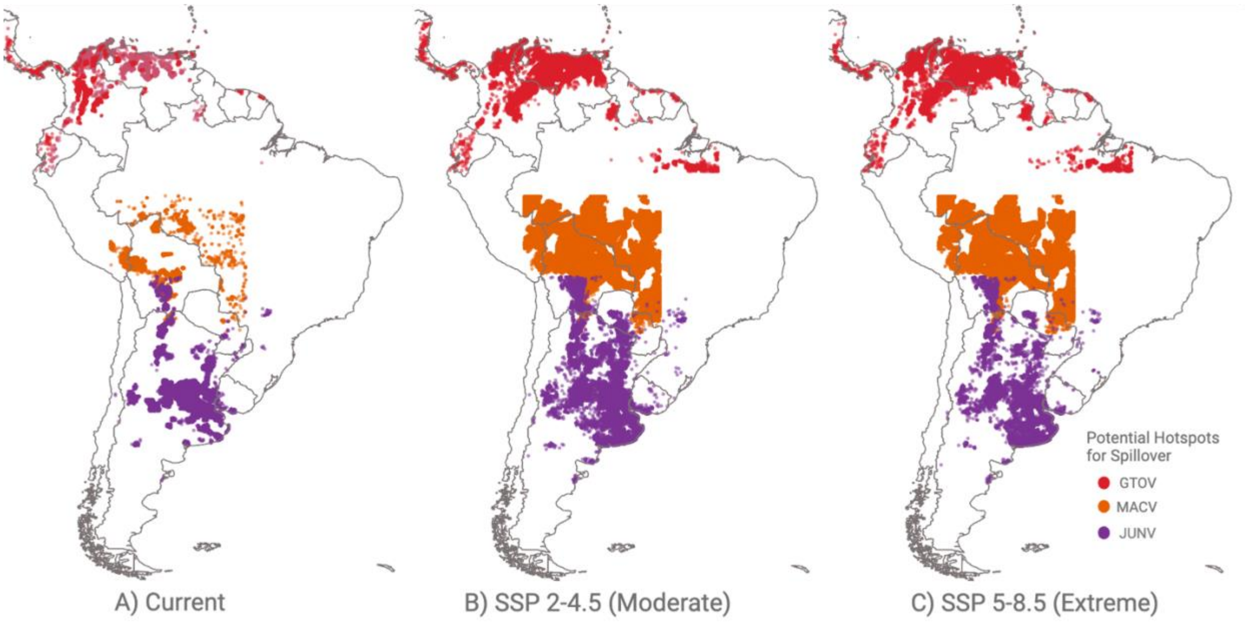

Fig 1.1 Each hotspot represented the top 10% percentile of FOI for successful transmission of NWAs from rodent reservoirs to humans. For each of the NWAs, the hotspots generated were combined for the respective rodent species such that the potential hotspots for GTOV, MACV and JUNV represented the combined risk of spillover into humans from three, one and two rodent reservoirs respectively (see methodology for details). Each species distribution pattern gave rise to different hotspot zones that overall created the potential hotspots for GTOV spillover

In case of GTOV, the number of potential hotspots increased by 1.94X (+ 50,460 hotspots) and 1.96X (+ 51,270 hotspots) for SSP 2-4.5 and 5-8.5 scenarios compared to the current situation (53,290 hotspots). Compared to the current hotspots, for both SSP 2-4.5 and SSP 5-8.5 scenarios of years 2041-2060, more hotspot zones were seen inland for Venezuela, Columbia, Ecuador, Panama, Costa Rica, Guyana, Suriname and Northern part of Brazil, and some of the Caribbean Island nations. The number of hotspots in the coastal regions of these countries also were predicted to increase. Between SSP 2-4.5 and SSP 5-8.5, the predicted number of potential hotspots for SSP 5-8.5 were less but the pattern remained the same.

We predicted very steep rise in the number of potential hotspots of MACV in SSP 2-4.5 (12.8 X; + 54,990 hotspots) and SSP 5-8.5 (11.7X; +50,070 hotspots) compared to the current predicted hotspots (4,664 hotspots). The new as well as persistent hotspots which were concentrated mostly in central Bolivia, southern Peru, northern-most part of Argentina and some parts of western Brazil and Paraguay. Unlike GTOV, the SSP 2-4.5 and SSP 5-8.5 scenarios for years 2041-2060 saw a marked increase and more widespread predicted hotspots in the historically recorded habitat zone of the rodent reservoir in this area. Most of the country of Bolivia and a large portion of bordering western part of Brazil were projected to have numerous potential hotspots in SSP 2-4.5 and SSP 5-8.5. Relatively less marked increase was predicted for eastern Peru, northern Argentina and Paraguay in both the scenarios. In Southern Bolivia, northern Argentina, and Paraguay, we predicted an overlap of potential hotspots for MACV and JUNV (which causes Argentine Hemorrhagic fever in humans) for SSP 5-8.5 scenario. Less overlap was predicted for SSP 2-4.5.

The pattern of hotspots for both scenarios of climate change in the future was inverse compared to the other two viruses for JUNV. For SSP 2-4.5 and SSP 5-8.5, we estimated a reduction in

number of hotspots by 0.95X (-6,820 hotspots) and 0.7X (-42,150 hotspots) for SSP 2-4.5 and SSP 5-8.5 respectively compared to current situation (143,080 hotspots). However, the area covered by the potential hotspots was more spread out. Many parts of Argentina, Uruguay, Paraguay and Bolivia were predicted to have new potential hotspots in the future while most of the current hotspots were projected to remain persistent.

Potential hotspots for current, SSP 2-4.5 and SSP 5-8.5 scenarios for each of the six rodent reservoirs individually can be viewed in the following figure (Fig. 1.2).

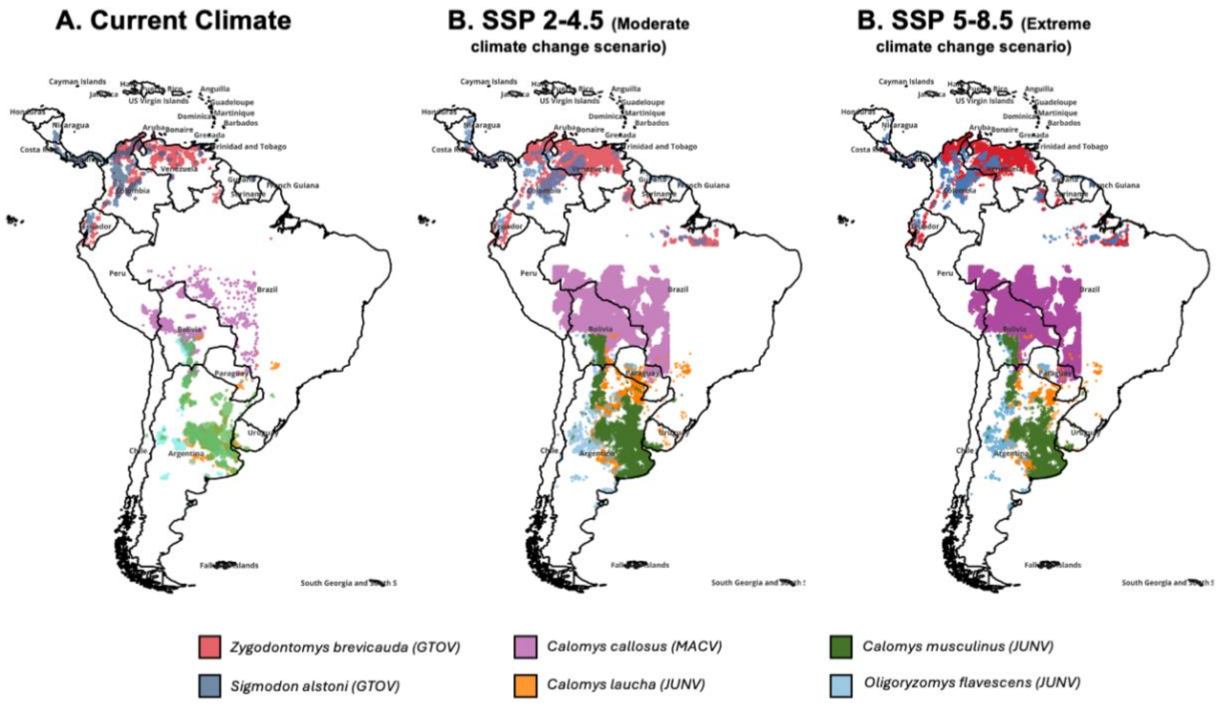

Figure 1.2 Potential hotspots for zoonotic spillover of New World Arenaviruses (NWAs) in –

(A) Current, and two future climate scenarios represented by (B) SSP 2-4.5 and (C) SSP 5-8.5 of the Shared Socio-economic Pathways (SSP) scenarios in Coupled Model Intercomparison Project Phase 6 (CMIP6) climate change model. The three NWAs under study were Guanarito virus (GTOV) which causes Venezuelan Hemorrhagic Fever (VHF), Machupo virus (MACV) which causes Bolivian Hemorrhagic Fever (BHF) and Junin virus (JUNV) which causes Argentine Hemorrhagic fever (AHF). Depicted hotspots are estimated based on top 10% percentile of Force of Infection (FOI) estimates for six rodent reservoirs, namely, *Zygodontomys brevicauda* (short-tailed cane mouse), *Sigmodon alstoni* (Alston's cotton rat) which are reservoirs of GTOV, *Calomys callosus* (large vesper mouse) which is the reservoir of MACV, *Calomys musculinus* (dryland vesper mouse), *Calomys laucha* (small vesper mouse) and *Oligoryzomys flavescens* (yellow Pygmy rice rat) which are the reservoirs of JUNV.

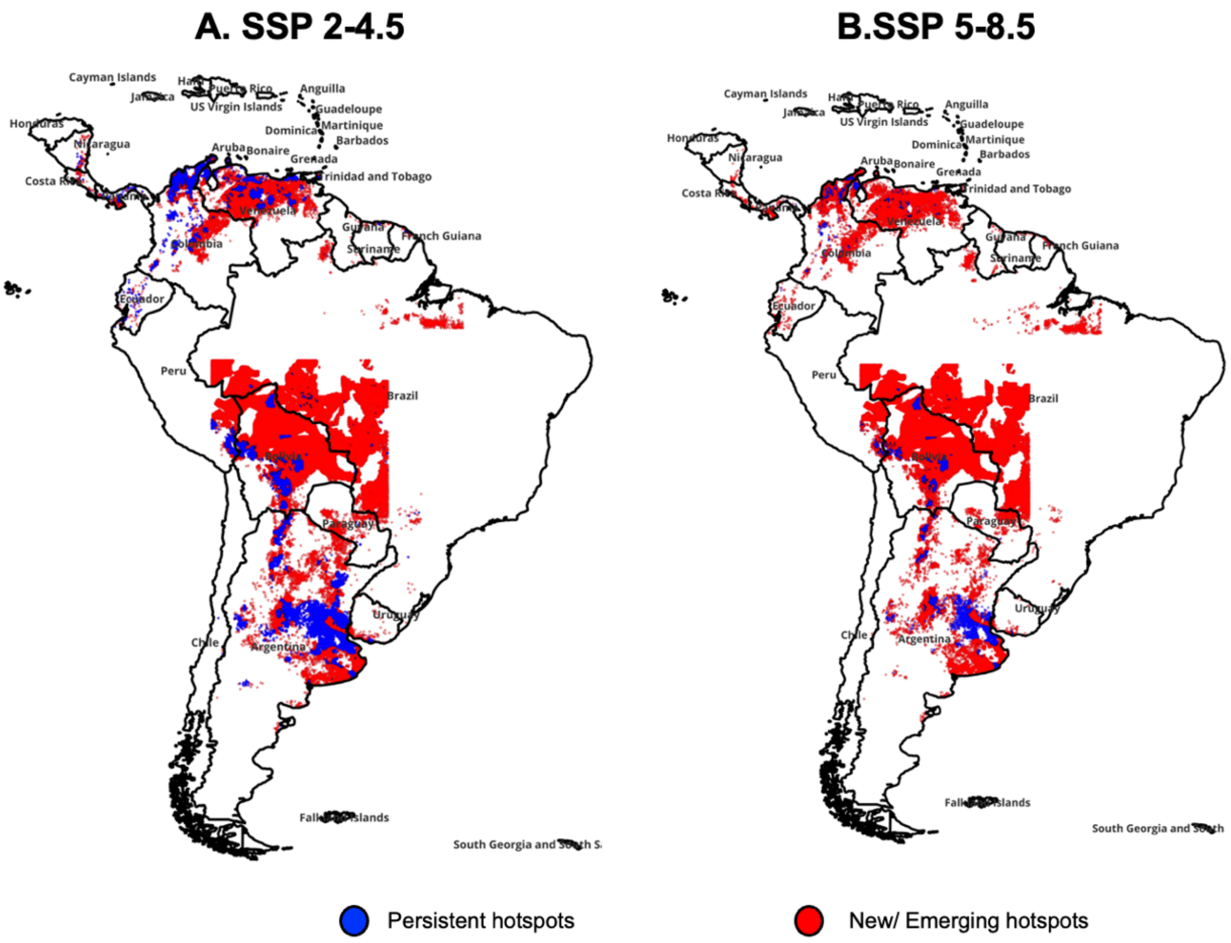

Figure 1.3. Map of predicted new (emerging) and persistent potential hotspots for zoonotic spillover of NWAs based on comparison of current climate to future climate change scenarios,

(A) SSP 2-4.5 and (B) SSP 5-8.5. In (A) figure shows in general more potential hotspots for moderate climate change scenario (SSP 2-4.5) compared to (B) Extreme climate change scenario (SSP 5-8.5). Also, (A) has more hotspots that remain persistent from current climate timeframe compared to (B) which shows newer hotspots that do not correspond to the current climate.

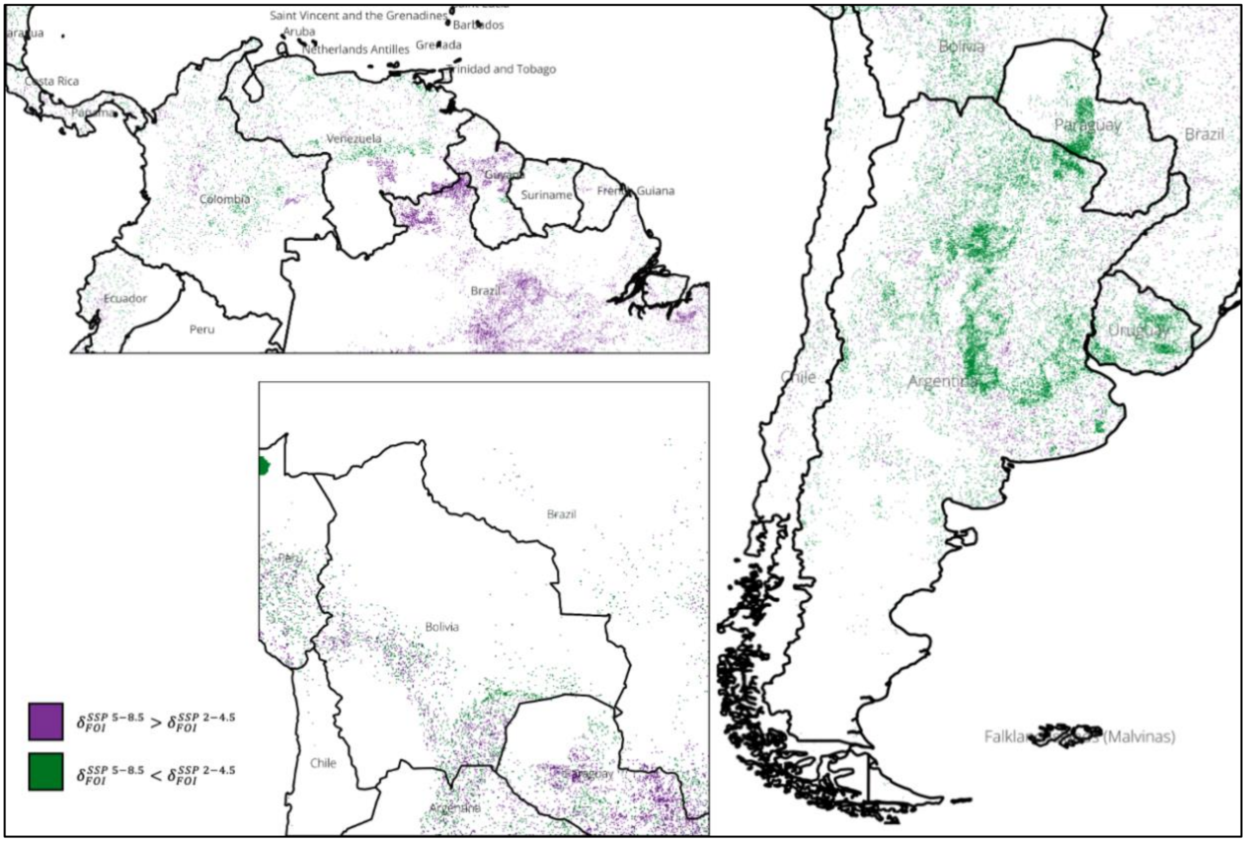

Figure 1.4. Maps showing the difference of difference in Force of Infection ($\delta_{FOI}^{SSP\ 2-4.5} - \delta_{FOI}^{SSP\ 5-8.5}$) between SSP 2-4.5 and SSP 5-8.5 climate change scenarios. Grid cells with no difference (rounded to 3 digits after decimal point) are not shown on the map.

Top-left: Guanarito virus (GTOV); Bottom-left: Machupo virus (MACV); Right: Junin virus (JUNV).

In terms of potential hotspots for spillover (defined as highest 10%ile FOI estimates), we predicted that the risk of zoonotic transmission for all modeled NWAs increased 5.7 times (~ 700,000 hotspots) in moderate (SSP 2-4.5) and 5.3 times (~ 650,000 hotspots) in extreme (SSP 5-8.5) climate change scenarios, when compared with the current distribution of risk (~ 120,000 hotspots, Supplementary Figure 1.1 and 1.2). The increase in the number of potential hotspots were 7% higher (1.1X) for SSP 2-4.5 compared to SSP 5-8.5.

Section B: Species Distribution Models (SDMs) for rodent reservoirs of zoonotic New World Arenaviruses (NWA)

Table 2.1. Datasets and raster variables used in Species Distribution Models (SDMs)

Variable/ Raster name	Year range	Notes
**Occurrence data – GBIF** [22]		
Z. brevicauda	1891-2023	6142 reported (688 presence-only - unique)
S.alstoni	1911-2017	326 reported (62 presence-only - unique)
C.musculinus	1955-2021	2287 reported (194 presence-only - unique)
C.laucha	1900-2024	2898 reported (137 presence-only - unique)
O.flavescens	1900-2024	1136 reported (171 presence-only - unique)
C.callosus	1914-2004	2746 reported (165 presence-only - unique)
**Bioclimatic Variables - World Clim Version 2** [23,24]		
Annual Mean Temperature	1970-2000	Bclim 1
Mean Diurnal Range	1970-2000	Bclim 2
Isothermality	1970-2000	Bclim 3
Temperature Seasonality	1970-2000	Bclim 4
Max Temperature of Warmest Month	1970-2000	Bclim 5
Min Temperature of Coldest Month	1970-2000	Bclim 6
Temperature Annual Range	1970-2000	Bclim 7
Mean Temperature of Wettest Quarter	1970-2000	Bclim 8
Mean Temperature of Driest Quarter	1970-2000	Bclim 9
Mean Temperature of Warmest Quarter	1970-2000	Bclim 10
Mean Temperature of Coldest Quarter	1970-2000	Bclim 11
Annual Precipitation	1970-2000	Bclim 12
Precipitation of Wettest Month	1970-2000	Bclim 13
Precipitation of Driest Month	1970-2000	Bclim 14
Precipitation Seaonality	1970-2000	Bclim 15
Precipitation of Wettest Quarter	1970-2000	Bclim 16
Precipitation of Driest Quarter	1970-2000	Bclim 17
Precipitation of Warmest Quarter	1970-2000	Bclim 18
Precipitation of Coldest Quarter	1970-2000	Bclim 19

**Elevation Data - SRTM v4 (NASA, CGIAR)**		
Digital Elevation Model (DEM)	2000	Elevation in meters
NDVI (MODIS (NASA - USGS)	2000	Normalized Difference Vegetation Index for minimum cloud cover
Primary Land (LCLUC (UMA, NASA))	1990-2005	lu_othr
Crop Land (LCLUC (UMA, NASA))	1990-2005	lu_crop
Urban Land (LCLUC (UMA, NASA))	1990-2005	lu_urbn
Secondary Land (LCLUC (UMA, NASA))	1990-2005	lu_secd
Pasture Land (LCLUC (UMA, NASA))	1990-2005	lu_past
Bioclimatic Variables set 1 (World Clim CMIP6 SSP 2-4.5)	2041-2060	
Bioclimatic Variables set 2 (World Clim CMIP6 SSP 5-8.5)	2041-2060	
Land Use 2.45 (LCLUC CMIP6 SSP245)	2041-2060	
Land Use 5.85 (LCLUC CMIP6 SSP585)	2041-2060	
NDVI (MODIS (NASA - USGS))	2020-present	
Digital Elevation Model (DEM) (SRTM v4 (NASA, CGIAR))	2000	Same as current data

Hyper tuning results

Across SDMs, optimal ensembles were generally moderate to large: for example, GTOV reservoir models converged to (RF 100–1,000 trees, ET 10–1,000, LGBM 100–200) with shallow-to-moderate tree depth (around 4–10) and small leaf nodes, often with unconstrained or flexible feature sampling (NULL/LOG2/SQRT). MACV models converged to sizeable ensembles (RF 500–750, ET 100–200, LGBM 500) with low-to-moderate depth (3–6), tight leaf constraints, and XGB configurations that used shallow trees with regularization (nonzero gamma and low min_child_depth). Similarly, JUNV models converged to sizeable ensembles (RF 500, 500 and 750, ET 100, 1000 and 200, LGBM 500 for all three reservoirs) with low-to-moderate depth (3–6 for all three), tight leaf constraints (small nodes), and XGB configurations that used shallow trees with regularization (nonzero gamma and low *min_child_depth*). Considering all the convergences were close to the default settings for *scikit-learn()* ensemble algorithms, we used default hyper parameters for the final models developed for GTOV, MACV and JUNV respectively.

2.1 Current species distribution maps

Guanarito virus

Guanarito virus which causes Venezuelan Hemorrhagic fever in humans has two identified rodent reservoirs, namely, *Zygodontomys brevicauda* (short-tailed cane mouse) and *Sigmodon alstoni* (Alston's cotton rat). Short-tailed cane mouse has a natural habitat in North-eastern part of the continent of South America (countries of Venezuela, Columbia, Guyana, Suriname, Northern part of Brazil and French Guiana) [25]. It can inhabit a varied set of environments ranging from forested and dense grass areas to open agricultural lands but prefers sites close to water bodies. Adults weigh up to 60-80 grams with no sexual dimorphism and a litter size of 5-

11 pups. These mice can survive from 1-5 years although detailed information on this is unavailable. Alston's cotton rat is found naturally in the North-eastern part of the continent of South America (countries of Venezuela, Columbia, Suriname, Guyana, Brazil and French Guiana) [25]. Its natural habitat is like other sigmodontid rodents that includes flatlands with shrubs, grass and open crop fields on low elevation lands. Most activity of the rats can be observed during daytime (diurnal). Unconfirmed reports of litter size include 3-7 pups.

Using historical occurrence data from GBIF for both rodent reservoirs of Guanarito virus, we converted the presence-only data to presence-absence data that was combined with the environmental and ecological raster data from various sources (see main text) to create species distribution maps. The SDM maps for current distribution can be seen in Figure 2.1 (a) and Figure 2.1 (b).

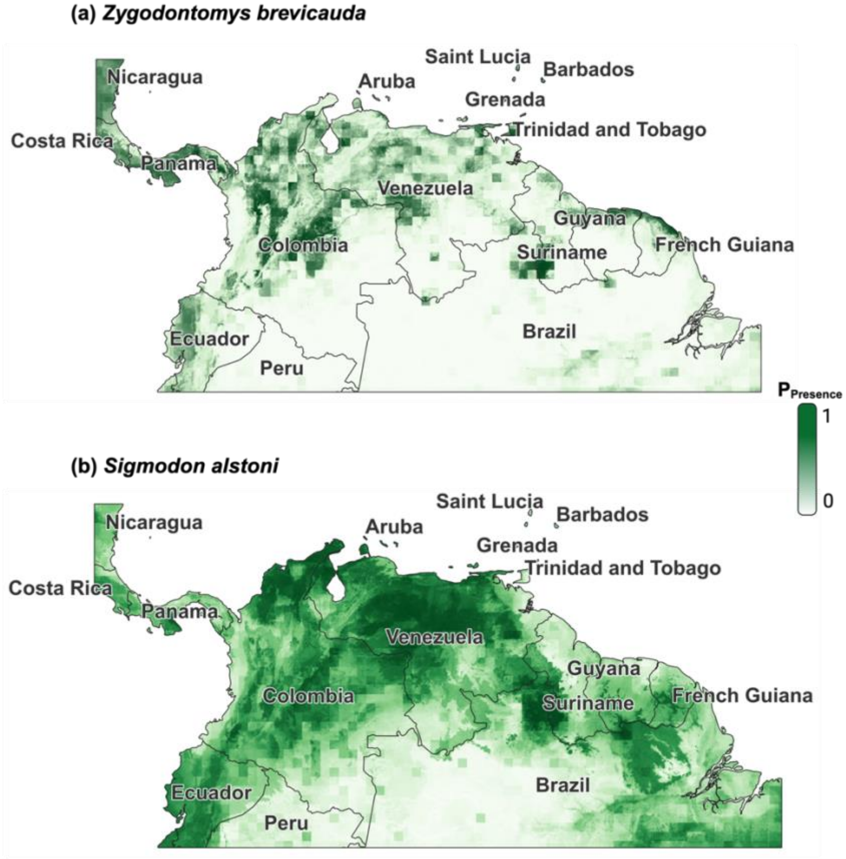

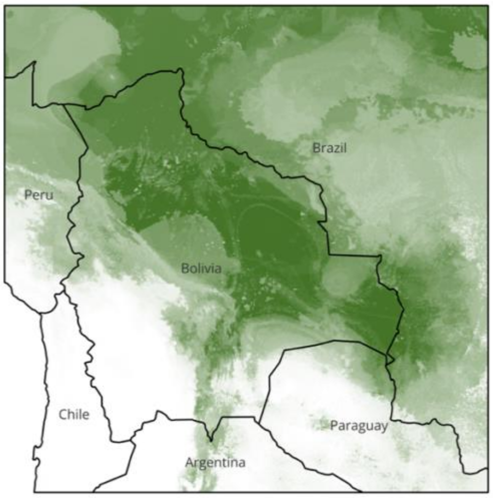

Figure 2.1. Current species distribution maps for the rodent reservoirs of the two rodent reservoirs of Guanarito virus and Machupo virus showing probabilities of presence (from 0 to 1); combined outcomes from all four ensemble algorithms.

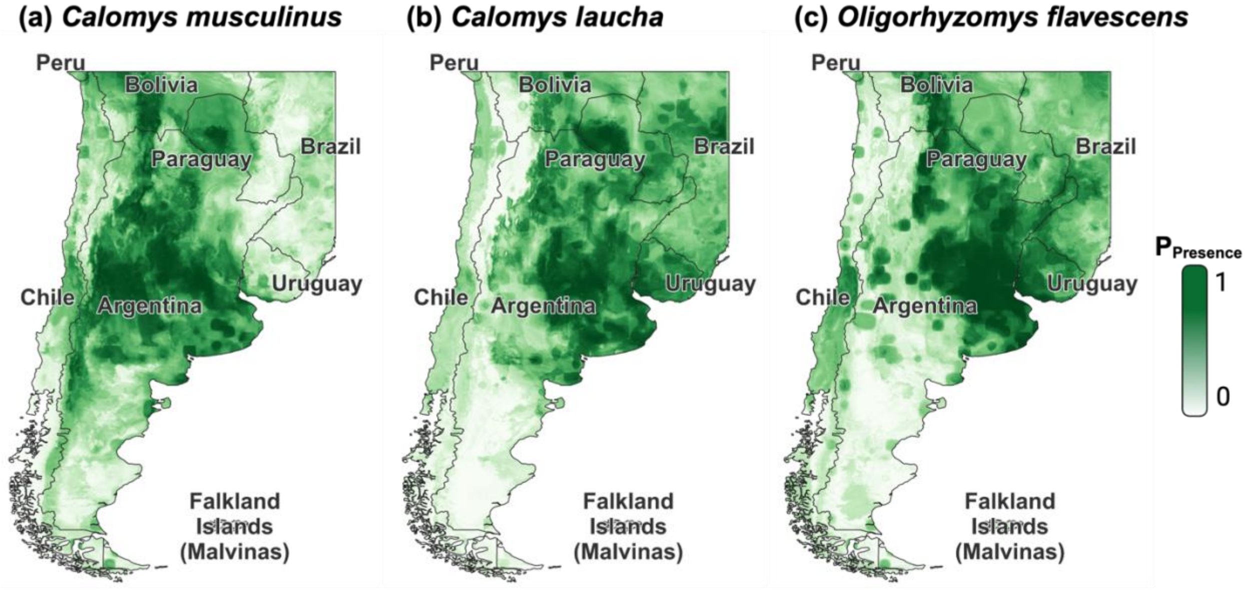

Figure 2.2 (a), (b) and (c). Current species distribution maps for the three rodent reservoirs of Junín virus showing probabilities of presence (from 0 to 1); combined outcomes from all four ensemble algorithms.

Z.brevicauda

Higher probabilities of presence were seen around the national parks in Columbia, Venezuela, Panama, Ecuador, Guyana and Suriname (Fig 2.1 a). The probabilities of presence were lower than threshold ($p < 50\%$) near big metropolitan cities like Bogota, Medellin, Caracas, Georgetown, Quito in the corresponding countries. Only in Paramaribo and Panama City, the largest cities in Suriname and Panama respectively, higher probabilities of presence were estimated. This showed that the mouse species is generally present in forested and bordering

grassland as well as cropped rural/ semi-urban areas. In general, the distribution patterns were most sensitive to seasonality and fluctuations in temperature, and cropland as well as other land use variables (Table 2.1).

S.alstoni

The higher probabilities of presence for this species also somewhat coincided with the other rodent, *Z.brevicauda* near the central areas of Venezuela, center-east of Columbia, Boa vista border between Venezuela and northern Brazil, near all the national forests (Figure 2.1 b). However, the key difference was that higher probabilities were estimated for zones in and around the cities of Cartagena, Bogota in Columbia, Caracas, Maracaibo, Valencia in Venezuela which are some of the largest metropolitan areas. Whereas Georgetown in Guyana, Paramaribo in Suriname, Panama City in Panama displayed less than threshold presence probabilities. In general, the SDMs showed that the species presence is significantly higher in populated peri-urban and urban areas especially those with flatlands and presence of cropped or grassed areas surrounding cities. Like, *Z.brevicauda*, *S.alstoni* is also expected to be present near dense forested areas in the national or state park areas. In general, the species distribution was most sensitive to precipitation and temperature seasonality, especially during the dry season (Table 2.1).

Machupo Virus

Machupo virus which causes Bolivian hemorrhagic fever in humans when spillover occurs, has one identified rodent reservoir species, namely, *Calomys callosus* (large vesper mouse). Large vesper mouse is a nocturnal mice species that is generalist in its habitat and has a wide distribution. Found primarily in Bolivia, it has also been reported in Argentina, Paraguay, and Brazil [25]. Due to their generalist habitation patterns, they can inhabit a variety of ecological niches. Litter sizes range between 3-5 pups and the adults who weigh between 30-50 grams, do not show sexual dimorphism. The SDM maps for current distribution of large vesper mouse can be seen in Figure 2.1 c.

Calomys callosus

Current SDMs of large vesper mouse show high probabilities of presence in the plains of Bolivia bordering the countries of Brazil, Argentina and Paraguay (Figure 2.1 c). Except for the Bolivian Andes regions, all regions show higher probabilities of presence than the threshold of 50%. The mouse species can be estimated to inhabit large diverse areas irrespective of rural or urban human settlements. The species presence, in general, was sensitive to features such as ranges of annual temperature and duration of day as well as total annual precipitation (Table 2.1).

Junin Virus

Junin virus which causes Argentinian hemorrhagic fever in humans has three identified rodent reservoir species, namely, Calomys *musculinus* (Dryland Vesper Mouse), *Calomys laucha* (Small Vesper mouse) and *Oligoryzomys flavescens* (Yellow Pygmy Rice Rat).

Dryland Vesper mouse is historically found in Argentina, Bolivia, and Paraguay where it occupies a wide variety of habitats, like natural grasslands, shrub steppes, crop field borders, and human-disturbed environments such as wastelands, railroads, and urban garbage dumps [26]. As the common name suggests, its abundance changes seasonally, driven by natural climatic variations and land-use practices that affect resource availability.

Calomys laucha or Small Vesper mouse is historically found in the Chaco plains of Argentina, Bolivia, Paraguay, southeastern Brazil, small parts of Uruguay and Chile. Adults weigh approximately 13 grams. The mice prefer dryer, flat plains with crops or grass covered areas and where soil is sandy that helps them create burrows. Litter size can range from 5 to 10 pups per litter, and they follow a monogamous mating and nesting system. Key ecological characteristics include seasonal changes to habitat selection where high vegetation areas are preferred in summer and winters are marked by more peri-domestic and open habitats.

Yellow pygmy rice rat is commonly found in Southern Brazil, Uruguay, Northern Argentina. These rats can inhabit arboreal; terrestrial be semi-aquatic areas and are typically spotted in pasture or croplands [25]. These rats are nocturnal in nature and do not move outside of natural home range. Very little is known about their reproductive behavior and unconfirmed reports include a litter size of 3-7 pups and seasonality in reproductive behavior. Adults can weigh around 40 grams.

The SDM maps for current distribution of the three rodent reservoirs for Junin virus can be seen in Figure 2.2.

Calomys musculinus

As seen in Figure 2.2 (a), higher probabilities of presence were estimated in the regions such as Pampas, Espinal, Monte de Llanuras y Mesetas, Chaco seco, Monte de Sierras y Bolsones, and Yungas which are situated in the basins of central Argentina. For details, see our other work [26]. The species distribution was mostly influenced by presence of urban land and total annual precipitation among other eco-environmental features (Table 2.2).

Calomys laucha

The species distribution patterns were estimated much like those of C.musculinus (Figure 2.2 (a, b)). The key differences were that lower probabilities than threshold of 50% were estimated in the Andean mountainous region bordering Chile and Argentina. Also, much higher probabilities were observed around metropolitan cities on coast of Argentina including Buenos Aires. The species distribution was most sensitive to features such as cropland and maximum recorded temperature in the warmest month; in other words the intensity of summer was a key feature for species distribution in general (Table 2.2).

Oligoryzomys flavescens

Compared to the other two reservoirs, the pygmy rat distribution patterns were slightly different in estimation. Highest probabilities were estimated in Cordoba, Entre Rios and Buenos Aires provinces in Argentina and coastal areas of Uruguay which surround the Rio de la Plata region at the confluence of Uruguay river and Parana river in the Pampa ecoregion (Figure 2.2 (c)).The species distribution was sensitive to features such as presence of crop or urban lands, total annual precipitation and precipitation during the warm period in general (Table 2.2).

2.2 Important features for current species distribution maps

The variability in the species distribution patterns of all the rodent reservoirs of the three NWAs under study were marked by variability in the importances of eco-environmental features that were used to predict the species distribution maps. Table 2.2 shows the top 5 most important features per the algorithm for all six of the rodent reservoirs of the three NWAs.

Table 2.2. Topmost important features for rodent reservoir species distribution models.

Species	Algorithm	Top 5 selected (number of times selected in 100 simulations with Recursive Feature Elimination)	Top 5 feature importances (feature importance in mean reduction of impurity – 2 decimal points)
Z.brevicauda	RF	• DEM (92)<br>• LUCrop (100)<br>• LUOthr (100)<br>• LUpast (100)<br>• LUurbn (100)	• LUothr (0.13)<br>• LUurbn (0.12)<br>• LUcrop (0.12)<br>• LUpast (0.09)<br>• LUsecd (0.06)
	ET	• Mean Diurnal Range (99)<br>• LUother (100)<br>• LUcrop (100)<br>• LUpast (100)<br>• LUsecd (100)	• LUothr (0.16)<br>• LUcrop (0.11)<br>• LUpast (0.1)<br>• Precipitation Seasonality (0.06)
	XGB	• Isothermality (94)<br>• LUSecd (95)<br>• LUurbn (95)<br>• LUcrop (100)<br>• LUothr (100)	• LUothr (0.18)<br>• LUcrop (0.17)<br>• LUurbn (0.07)<br>• LUpast (0.06)<br>• Isothermality (0.06)
	LGBM	• Temperature Seasonality (93)<br>• Isothermality (94)<br>• NDVI (96)<br>• LUcrop (97)<br>• Precipitation Seasonality (98)	• Isothermality (0.08)<br>• Temperature Seasonality (0.08)<br>• NDVI (0.07)<br>• LUcrop (0.07)<br>• Mean Diurnal Range (0.07)
S.alstoni	RF	• Precipitation Seasonality (91)<br>• Precipitation of Driest Month (93)<br>• LUpast (97)<br>• Temperature Seasonality (98)<br>• Precipitation of Driest Quarter (100)	• Precipitation of Driest Quarter (0.1)<br>• Temperature Seasonality (0.09)<br>• Precipitation of Driest Month (0.08)<br>• LUpast (0.08)<br>• Precipitation Seasonality (0.08)
	ET	• Temperature Seasonality (97)<br>• LUpast (97)<br>• Precipitation Seasonality (97)<br>• Precipitation of Driest Month (98)<br>• Precipitation of Driest Quarter (100)	• Precipitation Seasonality (0.08)<br>• Precipitation of Driest Quarter (0.08)<br>• LUpast (0.08)<br>• Precipitation of Driest Month (0.08)<br>• Temperature Seasonality (0.08)
	XGB	• LUpast (78)<br>• Isothermality (79)<br>• LUurbn (86)<br>• Precipitation of Driest Quarter (93)<br>• Temperature Seasonality (97)	• Precipitation of Driest Quarter (0.16)<br>• Temperature Seasonality (0.08)<br>• Precipitation of Warmest Quarter (0.07)<br>• LUpast (0.07)<br>• LUurbn (0.07)

	LGBM	• Precipitation of Wettest Month (81)<br>• Precipitation of Warmest Quarter (81)<br>• Mean Diurnal Range (82)<br>• Temperature Seasonality (88)<br>• LUurbn (96)	• NDVI (0.09)<br>• LUurbn (0.09)<br>• Temperature Seasonality (0.08)<br>• Precipitation of Wettest Month (0.08)<br>• Precipitation of Driest Quarter (0.07)
C.callosus	RF	• Annual Precipitation (96)<br>• Mean Diurnal Range (99)<br>• Precipitation of Wettest Month (100)<br>• Precipitation of Wettest Quarter (100)<br>• Temperature annual Range (100)	• Mean Diurnal Range (0.12)<br>• Precipitation of Wettest Month (0.09)<br>• Temperature Annual Range (0.09)<br>• Precipitation of Wettest Quarter (0.08)<br>• Min Temperature of Coldest Month (0.08)
	ET	• Annual Precipitation (96)<br>• Precipitation of Wettest Quarter (98)<br>• Precipitation of Wettest Month (99)<br>• Mean Diurnal Range (99)<br>• Temperature Annual Range (100)	• Temperature Annual Range (0.11)<br>• Mean Diurnal Range (0.09)<br>• Precipitation of Wettest Month (0.07)<br>• Annual Precipitation (0.07)<br>• NDVI (0.07)
	XGB	• Temperature Seasonality (81)<br>• Precipitation of Wettest Quarter (82)<br>• Mean Diurnal Range (86)<br>• Precipitation of Wettest Month (87)<br>• Temperature Annual Range (95)	• Mean Diurnal Range (0.2)<br>• Precipitation of Wettest Quarter (0.11)<br>• Precipitation of Wettest Month (0.09)<br>• Temperature Annual Range (0.08)<br>• Annual Mean Temperature (0.07)
	LGBM	• Isothermality (88)<br>• Mean Diurnal Range (89)<br>• NDVI (93)<br>• Temperature Seasonality (93)<br>• Temperature Annual Range (96)	• NDVI (0.1)<br>• Temperature Annual Range (0.08)<br>• Temperature Seasonality (0.08)<br>• Isothermality (0.08)<br>• Annual Precipitation (0.07)
C.musculinus	RF	• Mean Temperature of Wettest Quarter (92)<br>• Temperature Annual Range (95)<br>• Annual Precipitation (100)<br>• LUurbn (100)<br>• Mean Diurnal Range (100)	• LUurbn (0.11)<br>• Mean Diurnal Range (0.09)<br>• Annual Precipitation (0.08)<br>• Precipitation of Wettest Quarter (0.07)<br>• Temperature Annual Range (0.07)
	ET	• Temperature Annual Range (93)	• Mean Diurnal Range (0.07)

		• LUpast (94)<br>• Mean Diurnal Range (96)<br>• Mean Temperature of Wettest Quarter (97)<br>• Annual Precipitation (98)	• LUpast (0.07)<br>• Annual Precipitation (0.07)<br>• Mean Temperature of Wettest Quarter (0.07)<br>• Temperature Annual Range (0.07)
	XGB	• LUpast (82)<br>• Precipitation Seasonality (85)<br>• Mean Diurnal Range (96)<br>• Annual Precipitation (97)<br>• LUurbn (99)	• Annual Precipitation (0.11)<br>• LUurbn (0.1)<br>• Mean Diurnal Range (0.09)<br>• Temperature Annual Range (0.08)<br>• Precipitation Seasonality (0.07)
	LGBM	• Max Temperature of Warmest Month (87)<br>• LUpast (92)<br>• Mean Diurnal Range (96)<br>• LUSecd (96)<br>• LUurbn (100)	• LUurbn (0.11)<br>• Mean Diurnal Range (0.1)<br>• LUsecd (0.08)<br>• LUpast (0.08)<br>• Max Temperature of Warmest Month (0.07)
C.laucha	RF	• Max Temperature of Warmest Month (89)<br>• Annual Precipitation (90)<br>• Mean Temperature of Wettest Quarter (95)<br>• Temperature Annual Range (98)<br>• LUcrop (100)	• LUcrop (0.12)<br>• Temperature Annual Range (0.08)<br>• Mean Temperature of Wettest Quarter (0.07)<br>• Max Temperature of Warmest Month (0.07)<br>• Annual Precipitation (0.07)
	ET	• Max Temperature of Warmest Month (92)<br>• Annual Precipitation (93)<br>• Temperature Annual Range (94)<br>• Mean Temperature of Wettest Quarter (99)<br>• LUcrop (100)	• LUcrop (0.1)<br>• Mean Temperature of Wettest Quarter (0.08)<br>• Temperature Annual Range (0.07)<br>• Max Temperature of Warmest Month (0.07)<br>• Mean Temperature of Warmest Quarter (0.07)
	XGB	• Temperature Annual Range (82)<br>• Max Temperature of Warmest Month (82)<br>• LUurbn (89)<br>• Annual Precipitation (92)<br>• LUcrop (100)	• LUcrop (0.15)<br>• Annual Precipitation (0.08)<br>• LUothr (0.08)<br>• Precipitation of Coldest Quarter (0.07)<br>• LUpast (0.07)
	LGBM	• LUsecd (86)<br>• LUcrop (89)<br>• LUothr (90)<br>• LUpast (91)<br>• LUurbn (99)	• LUurbn (0.12)<br>• LUcrop (0.08)<br>• LUsecd (0.08)<br>• Lupast (0.08)<br>• Luothr (0.08)
O.flavescens	RF	• Mean Temperature of Wettest Quarter (88)	• LUurbn (0.16)<br>• LUcrop (0.09)

		• Precipitation of Warmest Quarter (98)<br>• Annual Precipitation (98)<br>• LUurbn (100)<br>• LUcrop (100)	• Precipitation of Warmest Quarter (0.07)<br>• Precipitation in wettest Month (0.07)<br>• Annual Precipitation (0.07)
	ET	• Precipitation of Warmest Quarter (93)<br>• Annual Precipitation (94)<br>• LUurbn (96)<br>• Mean Temperature of Wettest Quarter (98)<br>• LUcrop (100)	• LUcrop (0.09)<br>• Mean Temperature of Wettest Quarter (0.08)<br>• Annual Precipitation (0.08)<br>• Precipitation of Warmest Quarter (0.07)<br>• Mean Temperature of Coldest Quarter (0.07)
	XGB	• LUcrop (87)<br>• Max Temperature of Warmest Month (87)<br>• Annual Precipitation (92)<br>• Precipitation of Warmest Quarter (92)<br>• LUurbn (100)	• LUurbn (0.2)<br>• Annual Precipitation (0.1)<br>• Precipitation of Warmest Quarter (0.09)<br>• Precipitation of Wettest Month (0.08)<br>• Precipitation of Coldest Quarter (0.05)
	LGBM	• Annual Precipitation (88)<br>• Max Temperature of Warmest Month (90)<br>• LUsecd (91)<br>• LUcrop (94)<br>• LUurbn (100)	• LUurbn (0.13)<br>• LUcrop (0.08)<br>• Max Temperature of Warmest Month (0.08)<br>• LUsecd (0.07)<br>• Annual Precipitation (0.07)

Note: Abbreviations: RF: random forest algorithm, ET: Extra Trees algorithm, XGB: eXtra Gradient Boost, LGBM: Light Gradient Boost Model, LUcrop: Land use – cropland, LUurban: Land use – urban land, LUpast: Land use – Pasture land, LUsecd: Land use – secondary/ non-agricultural land, LUothr: Land use – Other/ Forested land use, NDVI: Normalized Difference Vegetation Index.

In contrast to Table 2.2, the Figure 2.2 shows the feature importance of all the 26 eco-environmental features for all six rodent reservoirs distributed over the 4 algorithms of 3 NWAs.

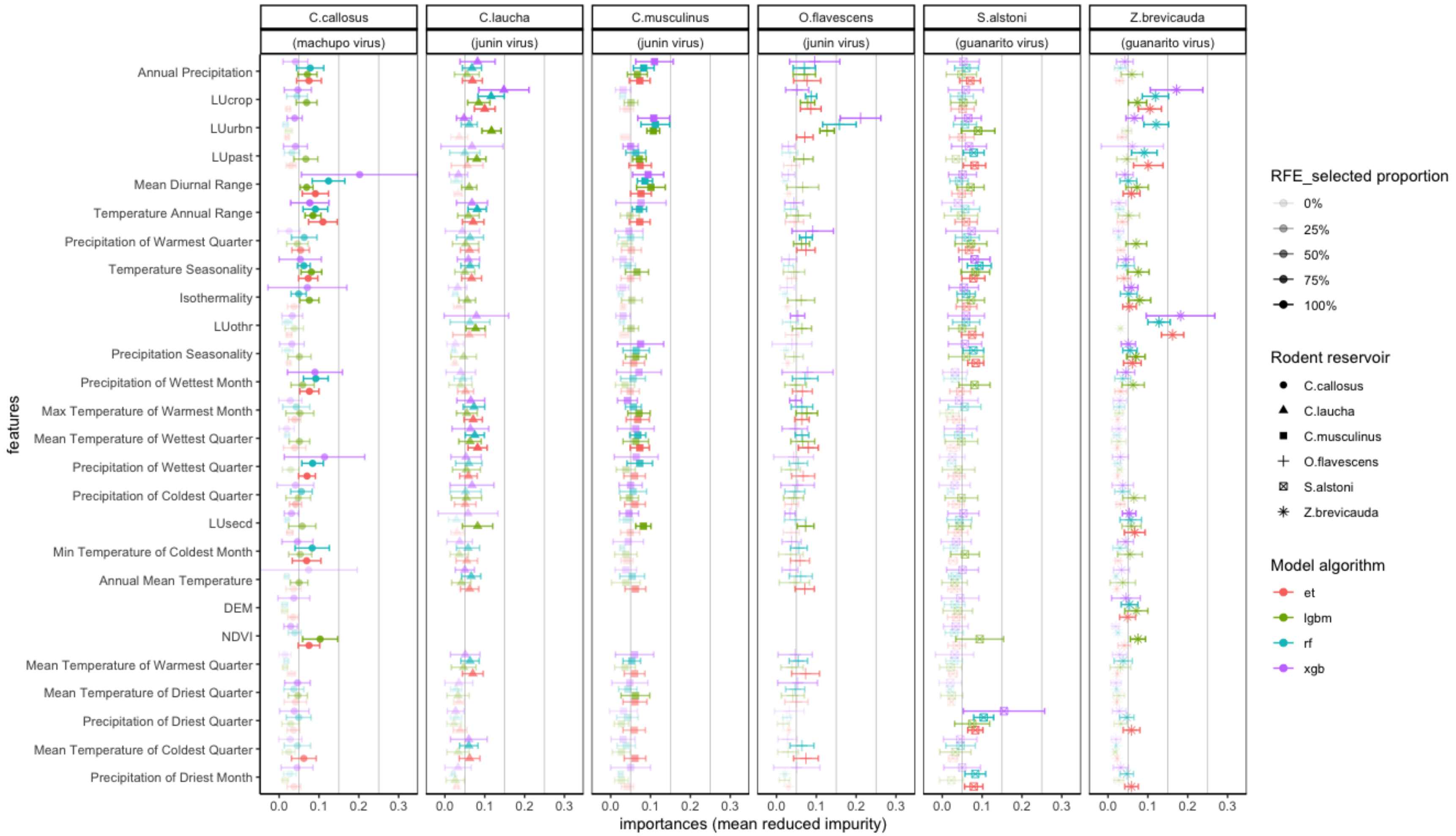

Figure 2.3. Feature importances of all 26 raster features based on permutations of mean reduced impurity (out-of-bag resampling) from the four ensemble algorithms along classified based on number of times the feature was selected in Recursive Feature Elimination (RFE) technique.

2.3 Modeling framework for changing risk of rodent-borne zoonotic diseases: Performance and Metrics

Species Distribution Models (SDMs) using four ensemble Machine Learning (ML) algorithms predicted the habitat suitability for six rodent reservoirs of NWAs under two future climate change scenarios (SSP2-4.5 and SSP5-8.5) in the next 20–40 years performed moderately well with a Cross-Validation (CV) accuracy between 77% to 87%. The recall score for species presence (recall for positives) ranged between 80% to 87% and the F1 scores were between 77% and 88% with a 12% to 20% false positive responses (Supplementary Figure 2.3A). The metrics based on the test set (25% of the overall data) were not dissimilar to the metrics on the CV set (Supplementary Figure 2.3B). In terms of predicting true presences, there were no clear differences in the confusion matrices (misclassification matrices; see Figure 2.4).

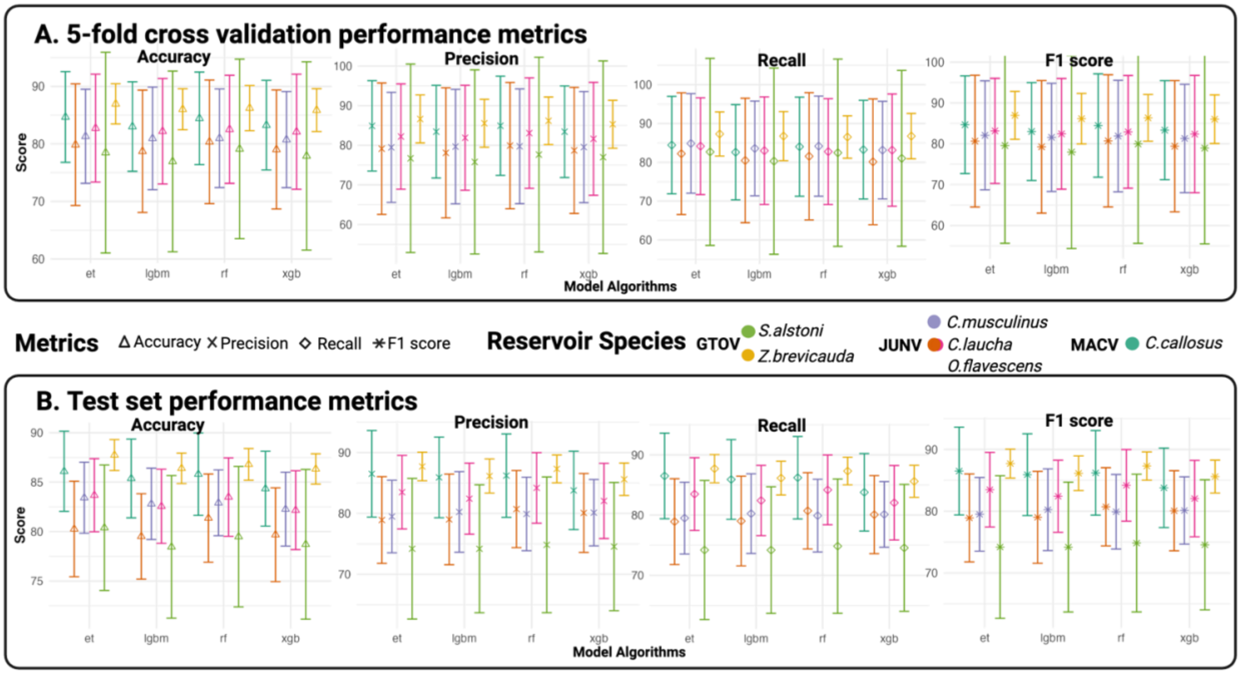

Figure 2.3. Prediction Performance metrics for the four model algorithms (rf: random forest, et: extra trees, xgb: extreme gradient boost , lgbm: light gradient boosting machine) used to develop Species Distribution Models (SDMs) for the six rodent reservoir species of the three New World Arenaviruses (NWAs), namely, Guanarito virus (GTOV), Machupo virus (MACV) and Junin virus (JUNV).

A. 5-fold cross-validation metrics. B. Test-set prediction metrics. All scores have % units.

The performance metrics magnitude was proportional to the number of historical occurrences for the rodent species in both validation and test datasets. This is in line with some of the prior studies which reported that the SDMs were sensitive to the number of positive signals in the sample data [27–30]. Further, the geospatial variation within the extent of each species' habitat also influenced the performance of the model in projecting species presence in future scenarios. This was conforming to prior literature in ensemble modeling in ecological studies [28,30–32]. The similarity between the predictive performance on both validation and test datasets suggested that the models were not biased or overfit to the training data.

Supplementary Material: Climate-driven changes in zoonotic risk of arenaviral hemorrhagic fevers in South America

PS. Kulkarni, NY. Flores-Perez, M. Uhart, BH. Bird, CK. Johnson, PS. Pandit.

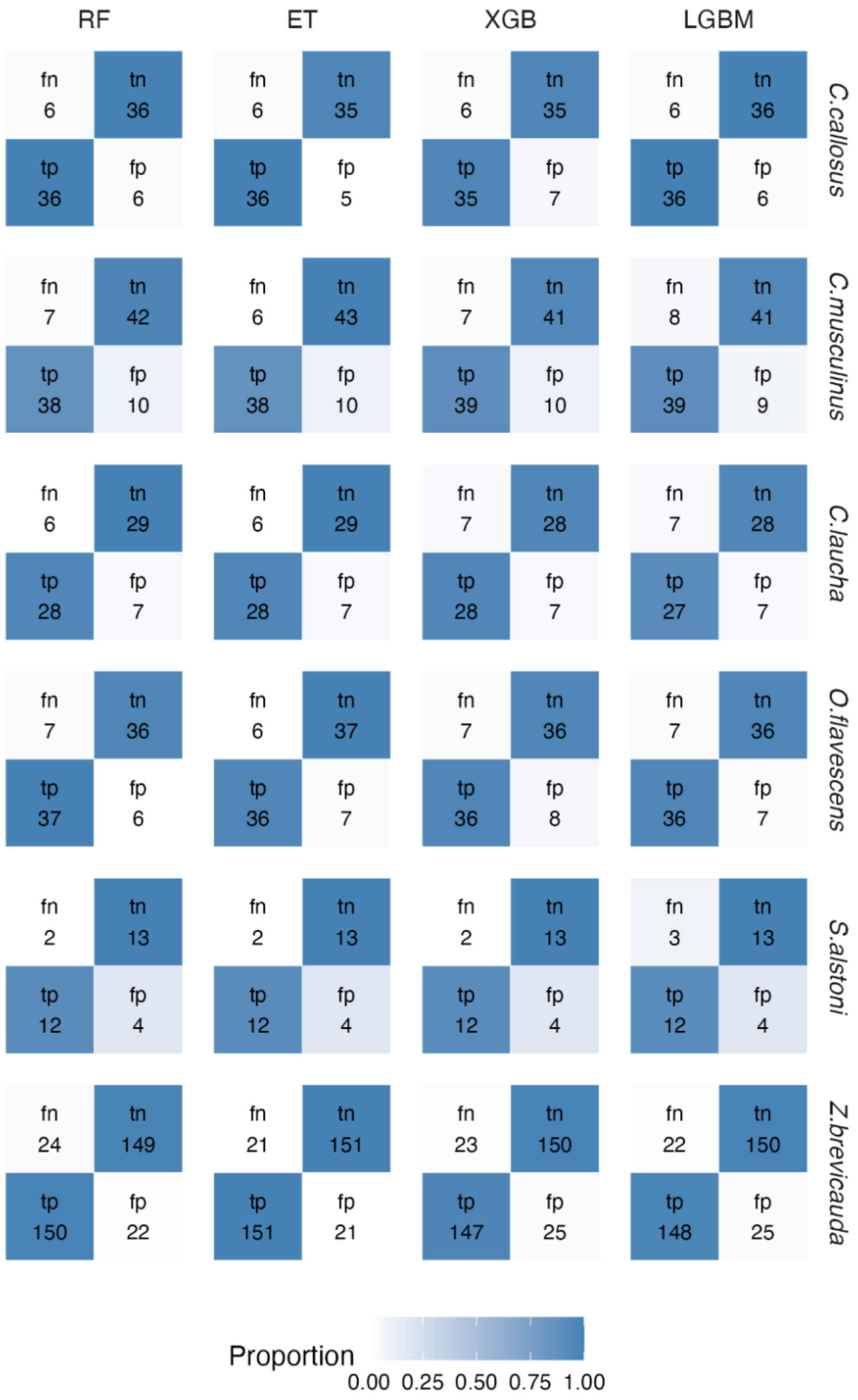

Figure 2.4. Confusion matrices for SDM model predictions over four algorithms for all six species of rodent reservoirs of NWAs (Abbrev; fn = false negatives, tp = true positives, fp = false positives, tn = true negatives)

2.4 Projected Future species distribution maps

Using CMIP6 models of climate change scenarios, we projected the distribution of the rodent reservoirs of Guanarito virus, Machupo virus and Junin virus in years 2041 to 2060. We selected two contrasting scenarios of SSP 2-4.5 or moderate climate change scenario and SSP 5-8.5 or extreme climate change scenario for this SDM projection. Using the same trained models of current SDMs, we imputed the probabilities for presence of the species in all the geographical locations in the current expanse of habitation for the rodent species as observed in the GBIF occurrence data.

In general, the projected SDMs had remarkably different probabilities of presence for the rodent species predicting a radical change in the habitat of the rodent reservoirs to be expected in the future. However, the differences in the probabilities of presence between the two CMIP6 scenarios, namely SSP 2-4.5 and SSP 5-8.5, were more subtle and hard to identify. Differences in probabilities of presence and distribution patterns of all six rodent reservoir species for NWAs is summarized in Table 2.3.

Table 2.3. Summary of delta maps (difference in probabilities of presence) for SSP 2-4.5 and SSP 5-8.5 compared to current species distribution maps (see Figure 2.3).

Virus	Species	CMIP6 Scenario	Increase in Probabilities	Decrease in Probabilities
**Guanarito Virus**	*Z.brevicauda*	SSP 2-4.5	Around rivers: Orinoco (Columbia), Branco (near Boa Vista), Amazon & tributaries (Xingu) in Brazil	Columbian Andes (north and south of Medellin)<br>River Sipapo<br>River Orinoco (Venezuela)<br>Marahuaca National Park<br>Venezuela-Brazil border (Neblina National Park)
		SSP 5-8.5	Higher intensity around rivers Branco, Amazon (Boa Vista, Manaus, Santarem, Tucurui)<br>River Meta (Columbia), River Apure (Venezuela)	National Park Cnaruco-Capanoparo<br>River Sipapo<br>South of Las Vegas (Venezuela)<br>Venezuela-Brazil border (Neblina National Park)
	S.alstoni	SSP 2-4.5	East & west of Guri Reservoir (San Pedro Forest, La Paragua)<br>River Cano Manamo (up to Gulf of Paria)<br>River Meta (Columbia-Venezuela border)<br>River Magdalena (north Columbia)<br>Northern border of Brazil (Guyana, Serra do Sol, Roraima)<br>South of Amazon Delta (Itacotiara to Mapaca)	Venezuela-Brazil border (Canaima National Park, Santa Inez)<br>River Caguan & tributaries (south-west Columbia)<br>Monkey Mountain & Karisparu (Guyana)<br>Central Suriname, French Guiana<br>Northern Brazil (Suriname & French Guiana border)

		SSP 5-8.5	Similar to SSP 2-4.5 with more intensity	Similar to SSP 2-4.5 with more intensity
**Machupo Virus**	*C.callosus*	SSP 2-4.5	West of Paraguay River (Gran Chaco basin, north of Concepcion)<br>River Pilcomayo (Bolivia-Argentina border)<br>Campo Grande (Brazil)<br>South of Salinas and Aguada Blanca (Peru)	West of Filadelfia (Paraguay)<br>Kaa Iya National Park (north Paraguay-Bolivia border)<br>West of River Bermejo (Argentina-Bolivia border)
		SSP 5-8.5	Tacna (Peru-Chile border)<br>East of River Cuiaba (Brazil)	Similar to SSP 2-4.5
**Junin Virus**	*C.musculinus*	SSP 2-4.5		<need to compare it with Nuri's work>
		SSP 5-8.5	<need to compare it with Nuri's work>	<need to compare it with Nuri's work>
	C.laucha	SSP 2-4.5	Gran Chaco plains (east of Andes in Argentina)<br>Paraguay (northern border with Brazil)<br>Rio Negro Province (Argentina)<br>Southern Bolivia-Argentina border (River Picomayo, Villa Montes, Camiri)<br>Maringa (Brazil)<br>Mato Grosso do Sul (Brazil)	Around Santa Cruz de la Sierra<br>Cordoba plains (Argentina)<br>around city of Junin<br>Mar del Plata (Atlantic coast)<br>Pampas (Argentina)<br>around Buenos Aires metropolis<br>Argentina-Uruguay border (Uruguay river)<br>Eastern Uruguay (Treinta y Tres)
		SSP 5-8.5	Similar to SSP 2-4.5	Similar to SSP 2-4.5 but more intensity around Inao National Park (Bolivia)<br>Tri-border area (Bolivia, Argentina, Paraguay)
	O.flavescens	SSP 2-4.5	Sporadic pockets in central plains (Cordoba, southern Buenos Aires)<br>Bolivian Andes (near Oruro)	Eastern Argentina (borders with Paraguay, Brazil, Uruguay)<br>East of Andes (Vallegrande to Villa Montes)
		SSP 5-8.5	More pronounced in Central Chile (Talca to Temuco)	Borders (Paraguay, Brazil, Argentina near Cuidad del Este)

Section C: Modeling choices and assumptions

3.1 CMIP6 models and SSP scenarios

The outcomes of this study assumed the validity of the climate change scenarios that were drawn from the SSP scenarios of the CMIP6 models. CMIP6 provided substantial granularity in resolution and more nuances in the prediction of climate change over CMIP5 [33]. The SSP 2-4.5 (Moderate Climate Change Scenario) and SSP 5-8.5 (Extreme Climate Change Scenario) are amongst the most well researched scenarios with counterparts in previous CMIP5 model [34]. It must be noted that neither of the scenarios meet the global climate agreement goals. Considering this, we believe that our outcomes should be viewed with the context of slow (SSP2.45) or no (SSP 5-8.5) progress towards sustainable climate goals. Based on unfolding geopolitical diplomacy surrounding climate change, these models can be extended to other scenarios featured in the CMIP6 suite.

3.2 Biodiversity of rodent reservoirs

In our study, we modeled the rodent reservoirs such as *Zygodontomys brevicauda* and *Sigmodon alstoni* for GTOV, *Calomys callosus* for MACV and *Calomys musculinus, Calomys laucha* and *Oligoryzomys flavescens* for JUNV. GTOV seroprevalence was reported and confirmed in *Z.brevicauda* and *S.alstoni* by multiple sources [35–37]. Tesh et al. also reported *Oligoryzomys fulverscens* and *Rattus rattus* as potential reservoirs for GTOV but subsequent literature did not confirm this [35,36]. Reservoir status of *C. musculinus, O. flavescens* and *C. laucha* for JUNV was repeatedly reported [38–40]. *C.callosus* was implicated as the rodent reservoir for both Machupo (MACV) and Chapare virus (CHAV) in Bolivia [38,41] and was recently confirmed as the reservoir for MACV [42].

Despite basing the selection of species to focus on based on previous accounts, there is significant ambiguity in exact number of species that were implicated as rodent reservoirs for NWAs [43,44]. Most NWAs have cospeciation and shared evolutionary history with the rodent reservoirs thereby being adaptable to the rodent species [45]. Also, presence of multiple species of NWA reservoirs in same geographical coordinates can indicate increased risk of transmission as well as bigger outbreak (controlling for human activities) [46–49]. Overlap of high human movement such as those of migrant farm labor and high diversity of rodent reservoir habitats would then constitute higher zoonotic risk [50].

3.3 Use of FOI-SDM framework

The FOI calculations used in this study to attribute risk profiles for spillover into human populations are based on assumption of density-dependent interaction and consequently, transmission of arenaviruses from the rodent populations to human population. There are several implicit assumptions in this calculation. Firstly, we assumed the transmission rate parameter of rodent populations based on the available literature pertaining to Lassa Fever Virus which is an Old-World Arenavirus (OWA). It is reasonable to expect that the NWA and OWA transmission to humans might not be comparable. However, we made this choice due to lack of transmission studies on NWA transmission to humans. Also, due to density-dependence, we assumed that the force of infection will asymptotically increase with the transmission rate parameter until a state of equilibrium is achieved. Secondly, the rodent populations were estimated based on their presence probabilities predicted from SDM outcomes. Depending on

which species is being modeled, the occurrences predicted from SDMs provide poor alternative to estimating population densities without input of validated data on species abundance while developing SDMs [51,52].

Given that the historical occurrence data from GBIF [53] was not informative in the standard population size of NWA rodent reservoirs, we estimated the rodent populations based on their predicted presence probabilities. Our estimation was approximately similar to the predicted litter size and family unit of rodent species by Han et al.[46] which is to date the only available literature on the group traits of New World rodents [54]. We theorize that this framework can be used for identifying areas for field work to generate transmission and outbreak data for neglected yet emerging diseases such as those caused by NWAs, that can validate the usefulness of these models.

3.4 Sampling bias in the data

We used presence-only occurrence data for rodent reservoir SDMs in this study that can introduce geographical sampling bias when generating naïve pseudoabsences [55,56]. Further, for machine learning approaches such as those in this study, the prediction accuracy is highly sensitive to the method in which pseudoabsences are generated [57]. To reduce this bias, we used the uniform approach of generating context-dependent pseudoabsences designed by Da Re et al. (2023) [55]. Numerous other approaches to generate ecologically consistent pseudoabsences have been used elsewhere in the literature. For example, EcoPA by Broussin et al. (2024) and three-step clustering approach by Senay et al. (2013) are viable alternatives [58,59].

Further, most NWAs outbreaks occur in rural areas within agrarian worker communities and when they occur, the patients exhibit non-specific symptoms of hemorrhagic fever that make rapid differential diagnosis difficult without molecular and genomic testing. A recent singular case of Chapare virus was reported by the International Health Regulations (IHR) National Focal Point (NFP) for the Plurinational State of Bolivia to WHO in January of 2025 [6060]. This occurred in La Paz department which is characterized by rural landscape bordering forested area. Studies like ours can serve as an important starting point for targeting high-risk hotspots for more granular surveillance that can serve as a more judicious allocation of resources rather than casting a wide net of passive monitoring.

Table 3.1. Permutation Importance summary for ∆FOI Random Forest Model across three modelled viruses and 2 SSPs scenarios.

Virus	GTOV				MACV				JUNV			
**Scenario**	**SSP 2-4.5**		**SSP 5-8.5**		**SSP 2-4.5**		**SSP 5-8.5**		**SSP 2-4.5**		**SSP 2-5.85**	
**Features**	**Mean**	**Std Error**	**Mean**	**Std Error**	**Mean**	**Std Error**	**Mean**	**Std Error**	**Mean**	**Std Error**	**Mean**	**Std Error**
Annual Mean Temperature	0.017	0.000	0.044	0.000	0.124	0.001	0.011	0.000	0.042	0.000	0.138	0.001
Mean Diurnal Range	-0.034	0.001	0.129	0.001	0.029	0.000	0.016	0.000	0.024	0.000	0.082	0.000
Isothermality	0.006	0.000	0.057	0.000	0.420	0.002	0.106	0.001	0.032	0.000	0.131	0.000
Temperature Seasonality	0.177	0.003	0.382	0.001	0.058	0.000	0.074	0.000	0.397	0.002	0.164	0.001
Max Temperature of Warmest Month	0.013	0.000	0.062	0.000	0.015	0.000	0.024	0.000	0.035	0.000	0.045	0.000
Min Temperature of Coldest Month	-0.003	0.000	0.046	0.000	0.235	0.002	0.143	0.001	0.039	0.000	0.048	0.000
Temperature Annual Range	-0.016	0.001	0.416	0.001	0.018	0.000	0.026	0.000	0.035	0.000	0.035	0.000
Mean Temperature of Wettest Quarter	-0.001	0.000	0.039	0.000	0.034	0.000	0.291	0.001	0.138	0.001	0.063	0.000
Mean Temperature of Driest Quarter	0.007	0.000	0.031	0.000	0.051	0.000	0.047	0.000	0.243	0.001	0.095	0.000
Mean Temperature of Warmest Quarter	-0.004	0.000	0.033	0.000	0.048	0.000	0.049	0.000	0.032	0.000	0.045	0.000
Mean Temperature of Coldest Quarter	0.010	0.000	0.033	0.000	0.014	0.000	0.015	0.000	0.031	0.000	0.241	0.001
Annual Precipitation	-0.063	0.001	0.101	0.000	0.193	0.001	0.114	0.001	0.114	0.001	0.067	0.000
Precipitation of Wettest Month	-0.008	0.000	0.101	0.000	0.041	0.000	0.059	0.000	0.081	0.001	0.061	0.000
Precipitation of Driest Month	0.002	0.000	0.023	0.000	0.032	0.000	0.212	0.001	0.078	0.001	0.012	0.000
Precipitation Seasonality	-0.010	0.001	0.059	0.000	0.067	0.000	0.070	0.000	0.116	0.001	0.083	0.000
Precipitation of Wettest Quarter	0.017	0.001	0.048	0.000	0.046	0.000	0.394	0.002	0.076	0.000	0.052	0.000
Precipitation of Driest Quarter	0.009	0.000	0.056	0.000	0.046	0.000	0.023	0.000	0.039	0.000	0.036	0.000
Precipitation of Warmest Quarter	-0.013	0.001	0.073	0.000	0.062	0.000	0.068	0.000	0.409	0.002	0.606	0.002
Precipitation of Coldest Quarter	-0.031	0.000	0.062	0.000	0.101	0.001	0.135	0.001	0.055	0.000	0.020	0.000
Crop land	-0.072	0.001	0.190	0.001	0.127	0.001	0.106	0.001	0.209	0.001	0.202	0.001
Other/ Forested land	-0.010	0.001	0.222	0.001	0.063	0.000	0.078	0.000	0.111	0.001	0.084	0.000
Pasture land	-0.024	0.001	0.078	0.000	0.079	0.000	0.133	0.001	0.152	0.001	0.178	0.001
Secondary land	-0.016	0.001	0.070	0.000	0.230	0.001	0.278	0.001	0.127	0.001	0.119	0.001
Urban land	0.006	0.000	0.048	0.000	0.103	0.000	0.148	0.001	0.455	0.001	0.421	0.001

3.5 Correlation between predictor features

Following are the correlation heatmaps between all the predictor variables. The list includes 18 bioclimatic variables, 5 land use variables, 1 NDVI and 1 DEM for each of the three climate scenarios, namely, current, SSP 2-4.5 and SSP 5-8.5. Each heatmap represents these three scenarios for the three viruses, namely, GTOV, JUNV and MACV. Along with that, the correlation between changes in values of rasters for current and SSP scenarios, namely SSP 2-4.5 ($\boldsymbol{\Delta_f^{ssp\ 2-4.5}}$) and SSP 2-5.85 ($\boldsymbol{\Delta_f^{ssp\ 5-8.5}}$) have also been plot for each of the three viruses.

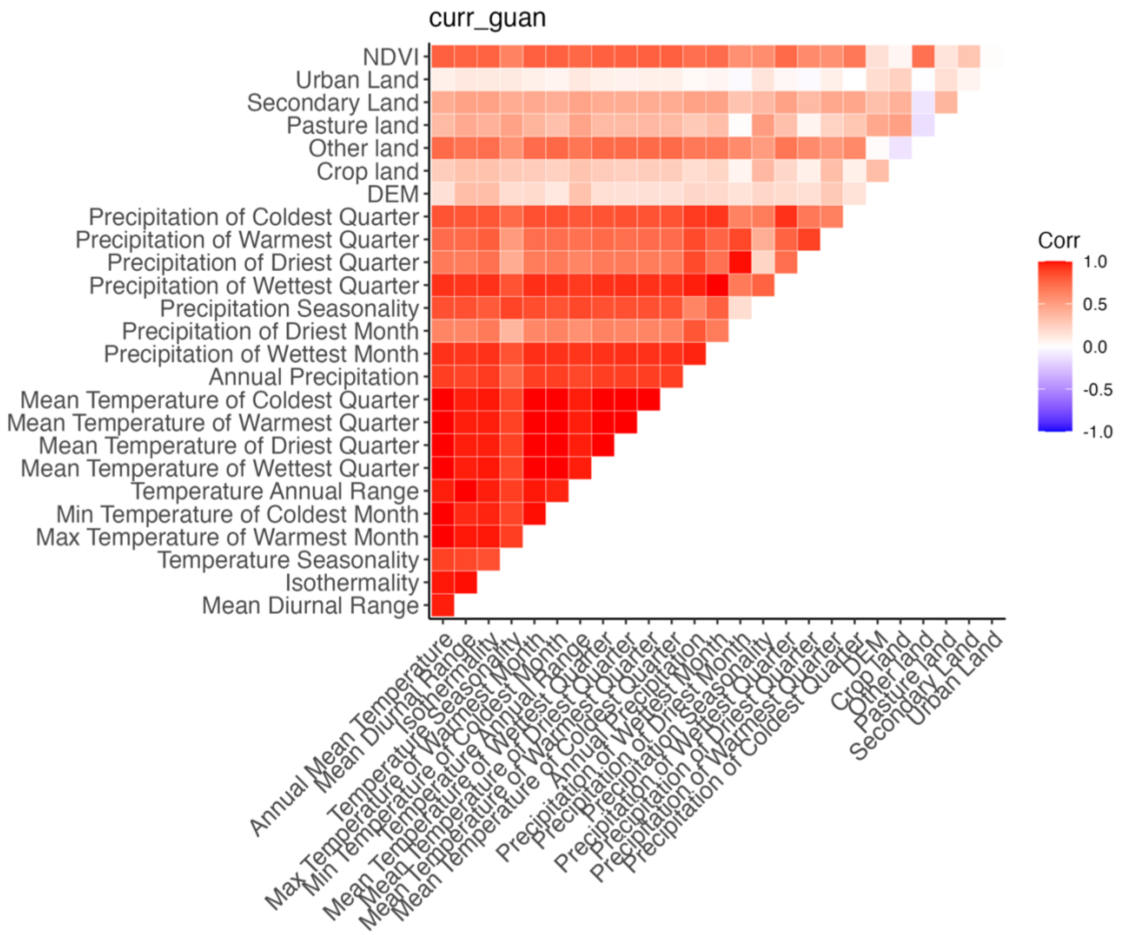

(a)

Supplementary Material: Climate-driven changes in zoonotic risk of arenaviral hemorrhagic fevers in South America

PS. Kulkarni, NY. Flores-Perez, M. Uhart, BH. Bird, CK. Johnson, PS. Pandit.

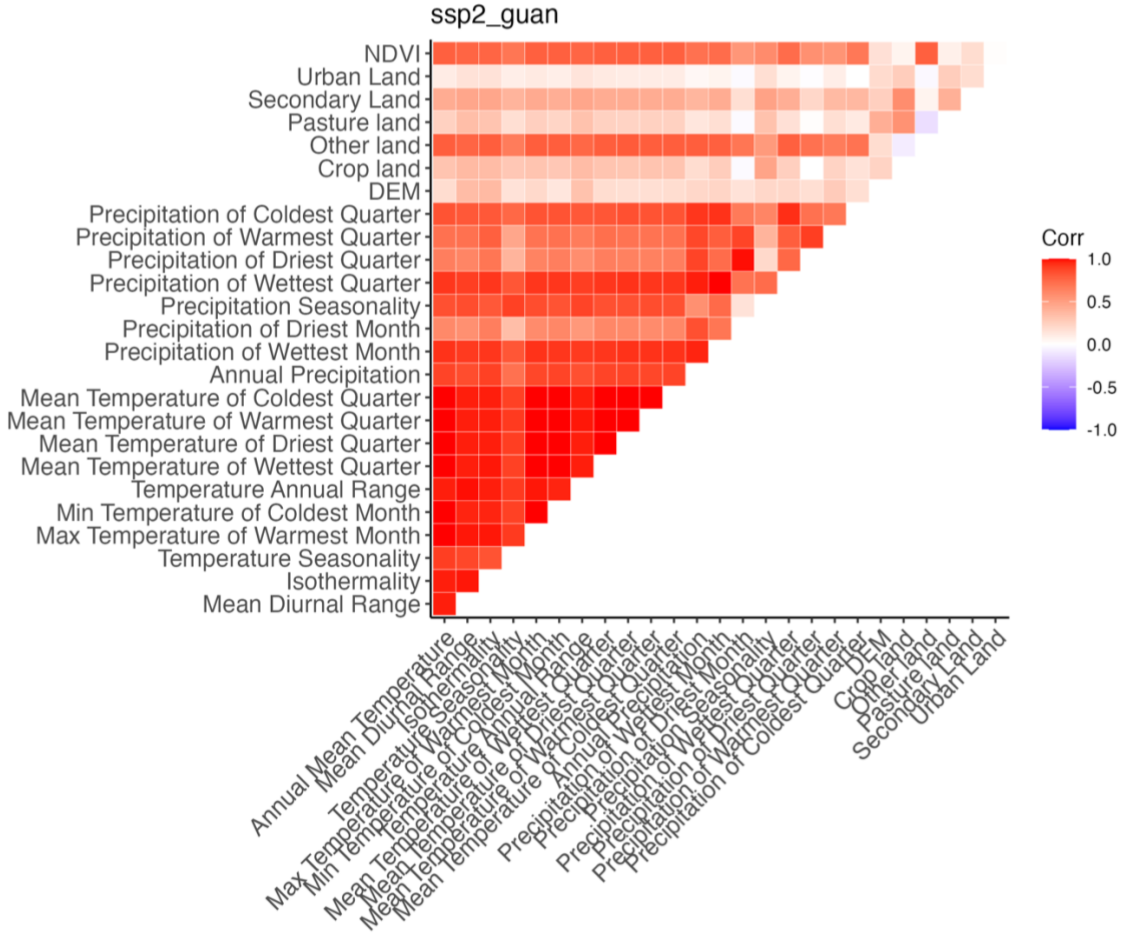

(b)

Supplementary Material: Climate-driven changes in zoonotic risk of arenaviral hemorrhagic fevers in South America

PS. Kulkarni, NY. Flores-Perez, M. Uhart, BH. Bird, CK. Johnson, PS. Pandit.

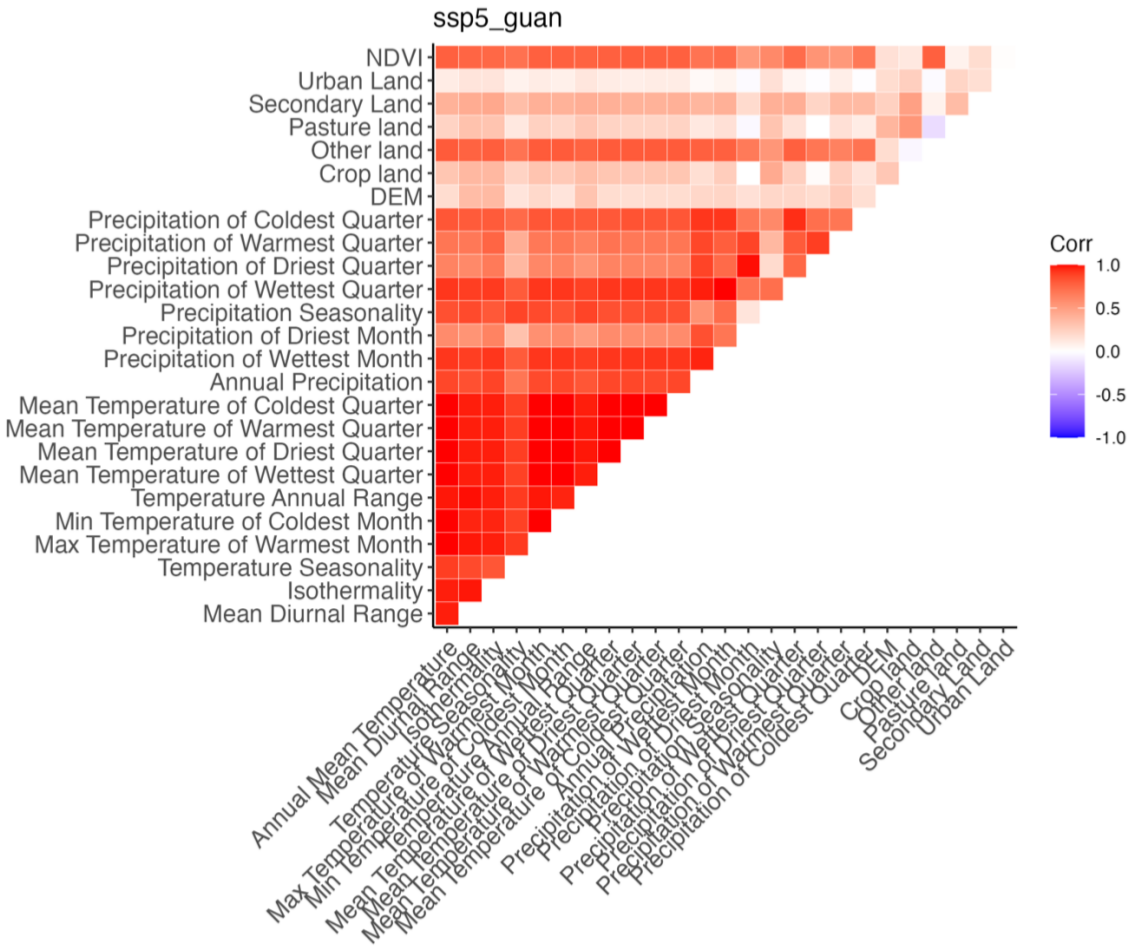

(c)

Supplementary Material: Climate-driven changes in zoonotic risk of arenaviral hemorrhagic fevers in South America

PS. Kulkarni, NY. Flores-Perez, M. Uhart, BH. Bird, CK. Johnson, PS. Pandit.

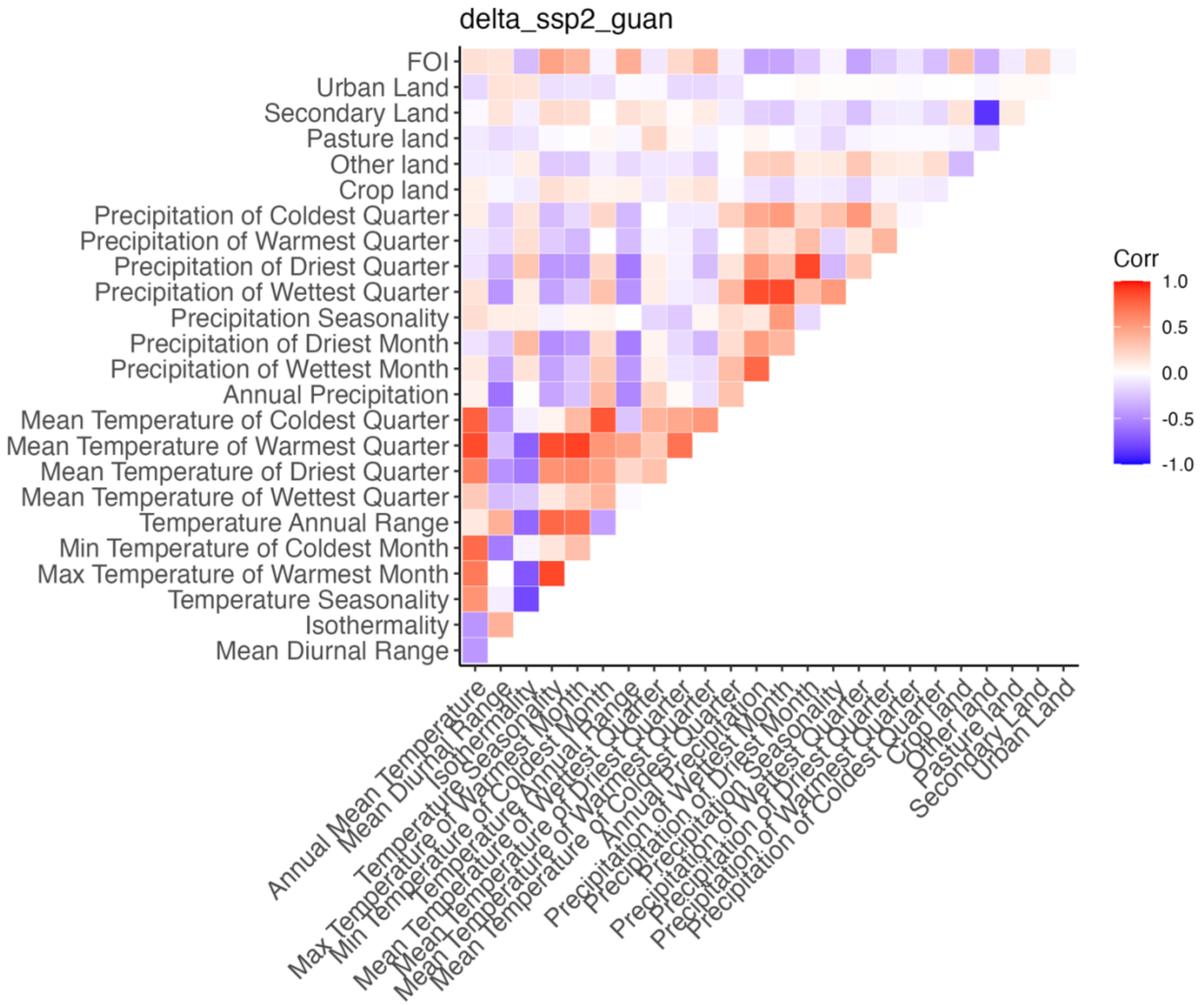

(d)

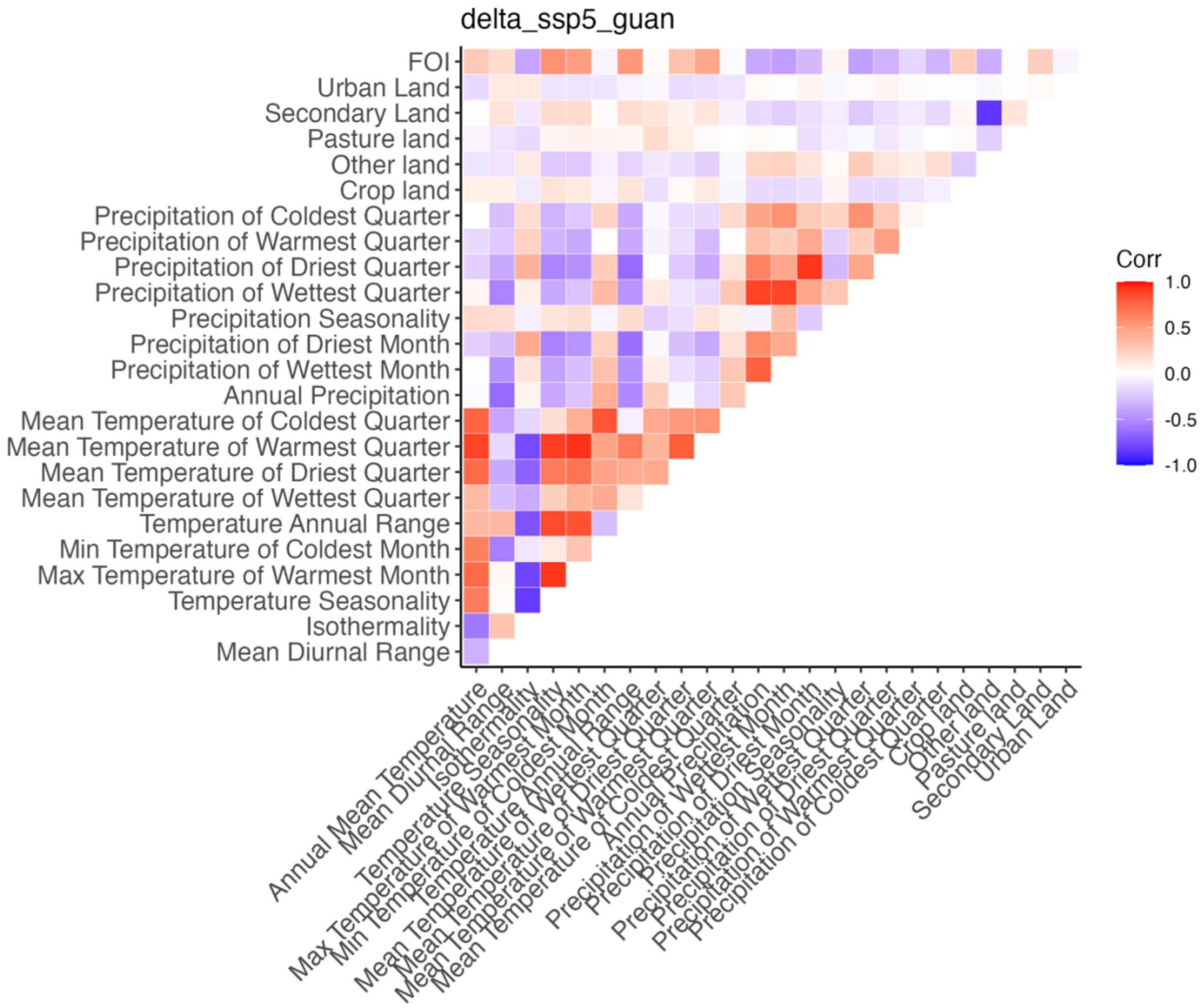

(e)

Fig 3.1 Pearson's correlation heatmap of predictive features for GTOV in –

(a) current climate scenario, (b) SSP 2-4.5 scenario, (c) SSP 5-8.5 scenario, (d) difference between current and SSP 2-4.5 values of the features ($\Delta_f^{ssp\ 2-4.5}$) and (e) difference between current and SSP 5-8.5 values of the features ($\Delta_f^{ssp\ 5-8.5}$)

Supplementary Material: Climate-driven changes in zoonotic risk of arenaviral hemorrhagic fevers in South America

PS. Kulkarni, NY. Flores-Perez, M. Uhart, BH. Bird, CK. Johnson, PS. Pandit.

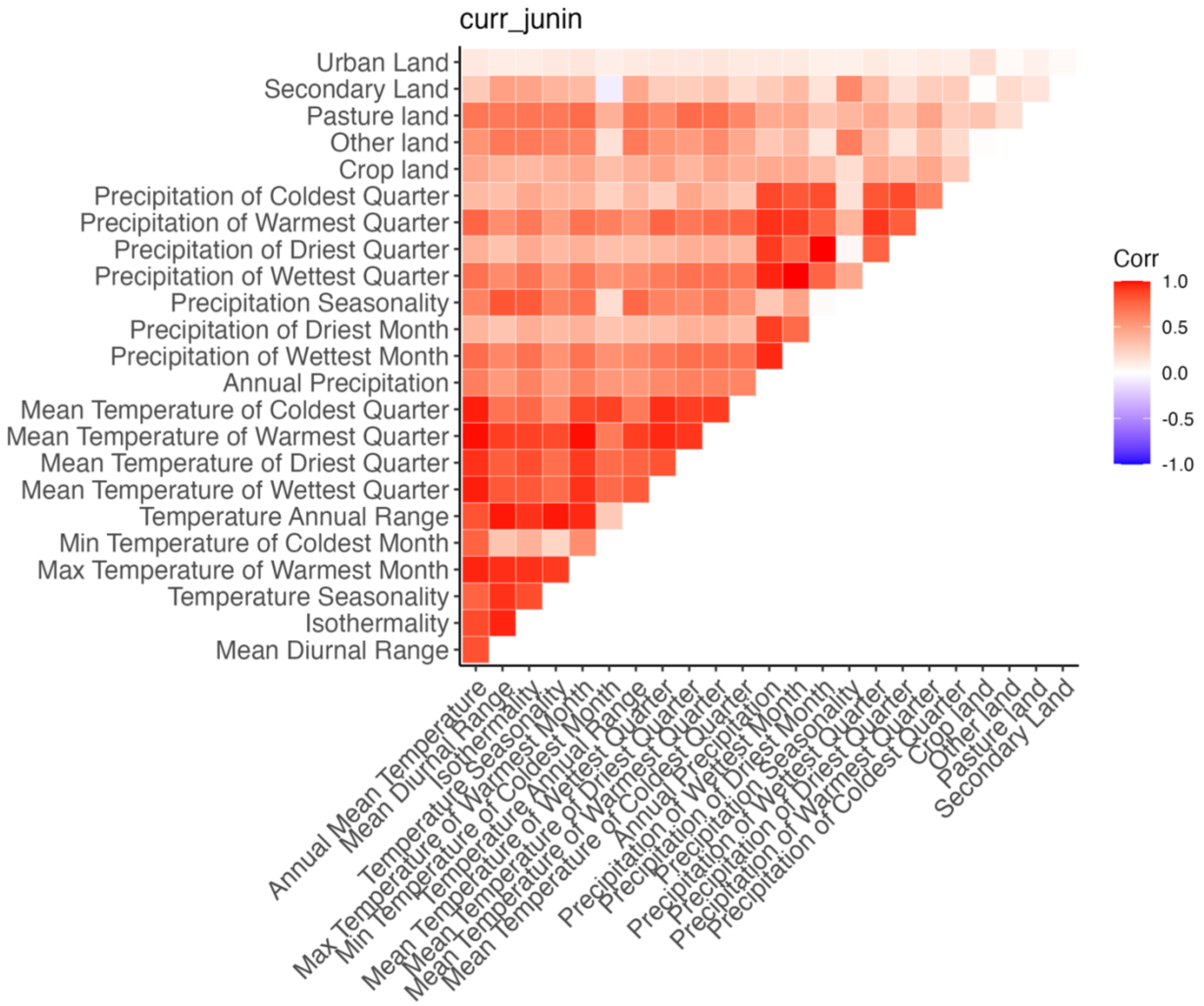

(a)

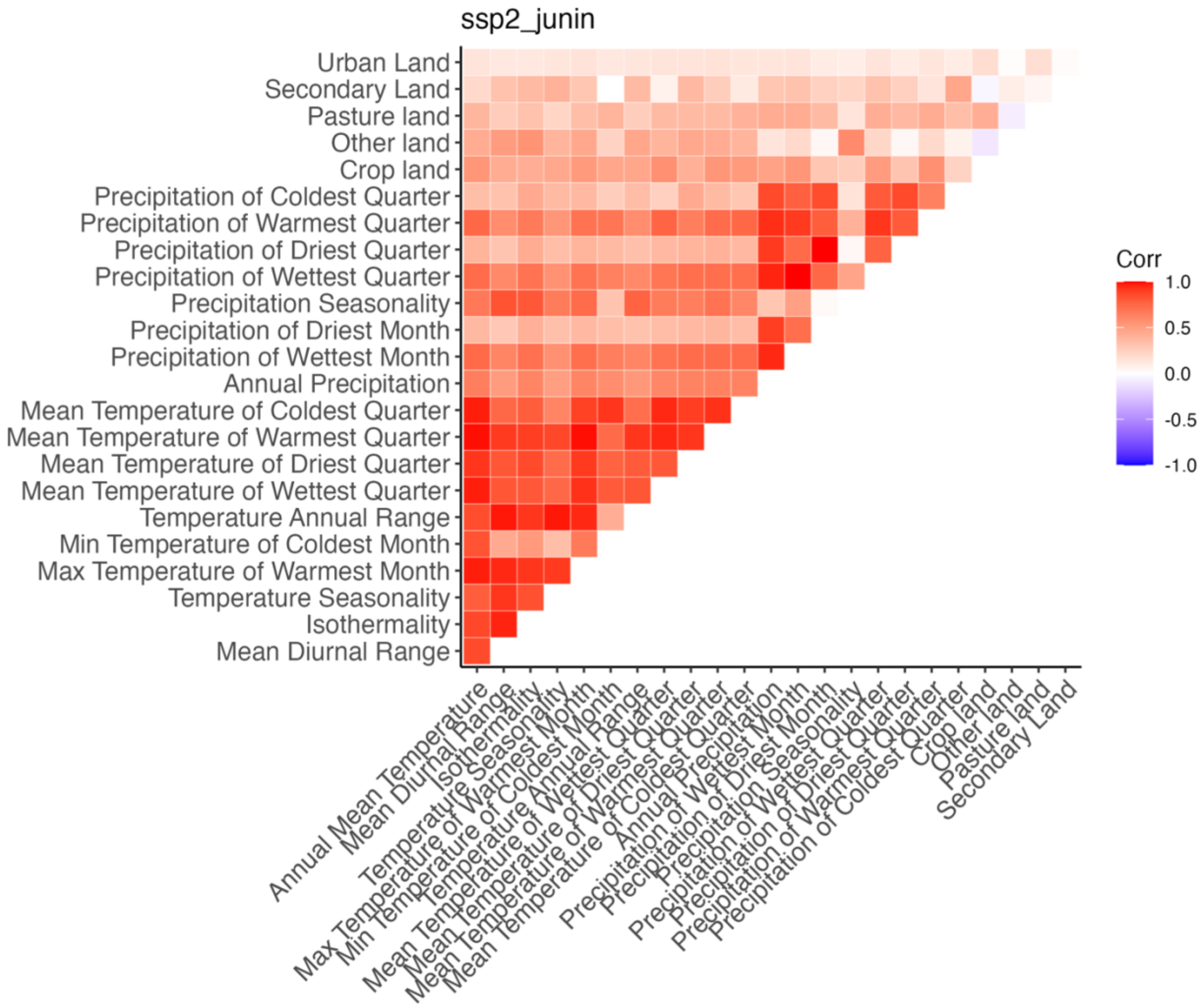
ssp2_junin
Urban Land
Secondary Land
Pasture land
Other land
Crop land
Precipitation of Coldest Quarter
Precipitation of Warmest Quarter
Precipitation of Driest Quarter
Precipitation of Wettest Quarter
Precipitation Seasonality
Precipitation of Driest Month
Precipitation of Wettest Month
Annual Precipitation
Mean Temperature of Coldest Quarter
Mean Temperature of Warmest Quarter
Mean Temperature of Driest Quarter
Mean Temperature of Wettest Quarter
Temperature Annual Range
Min Temperature of Coldest Month
Max Temperature of Warmest Month
Temperature Seasonality
Isothermality
Mean Diurnal Range
Annual Mean Temperature
Corr
1.0
0.5
0.0
-0.5
-1.0

(b)

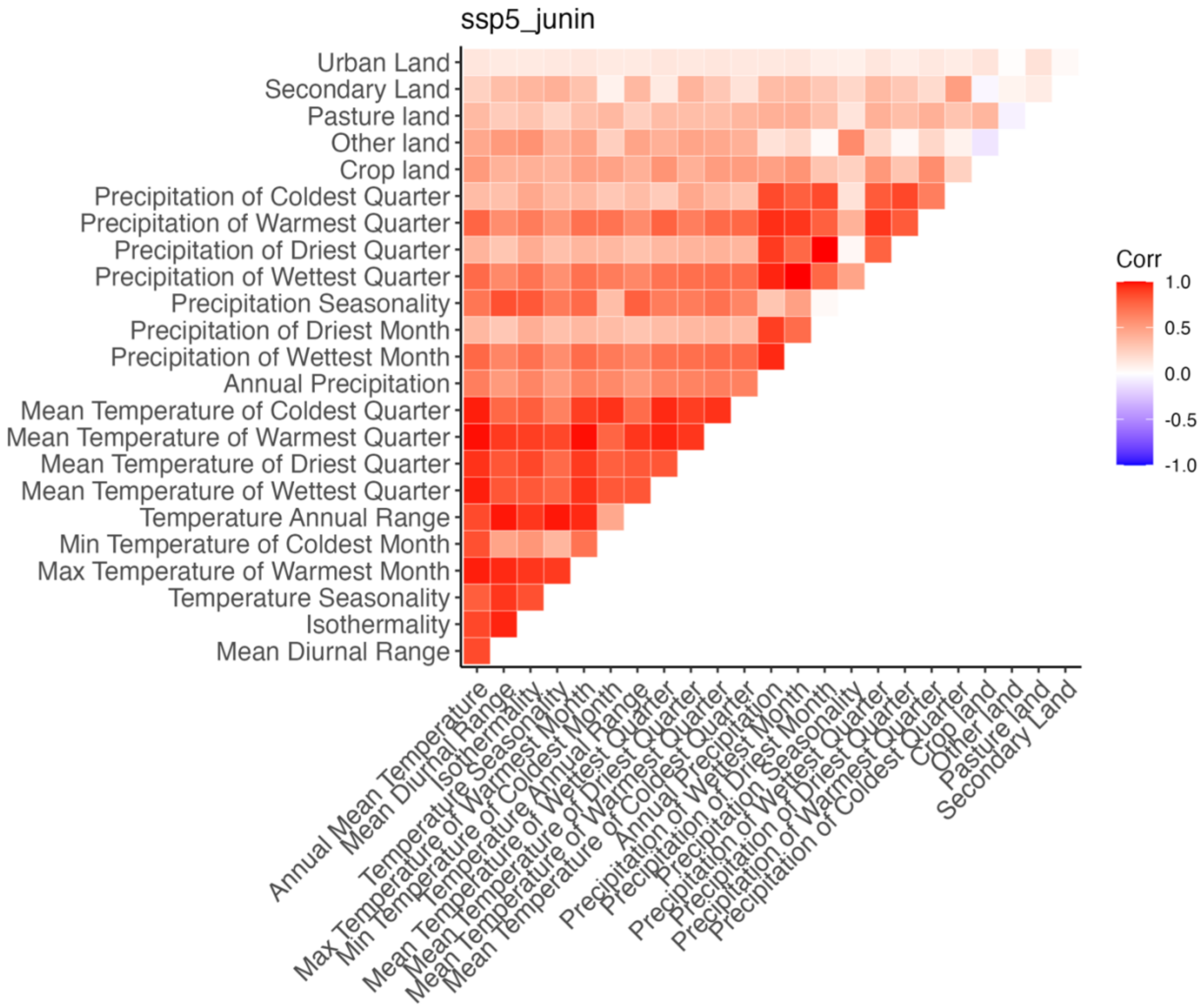
ssp5_junin
Urban Land
Secondary Land
Pasture land
Other land
Crop land
Precipitation of Coldest Quarter
Precipitation of Warmest Quarter
Precipitation of Driest Quarter
Precipitation of Wettest Quarter
Precipitation Seasonality
Precipitation of Driest Month
Precipitation of Wettest Month
Annual Precipitation
Mean Temperature of Coldest Quarter
Mean Temperature of Warmest Quarter
Mean Temperature of Driest Quarter
Mean Temperature of Wettest Quarter
Temperature Annual Range
Min Temperature of Coldest Month
Max Temperature of Warmest Month
Temperature Seasonality
Isothermality
Mean Diurnal Range
Annual Mean Temperature
Mean Diurnal Range
Isothermality
Temperature Seasonality
Max Temperature of Warmest Month
Min Temperature of Coldest Month
Temperature Annual Range
Mean Temperature of Wettest Quarter
Mean Temperature of Driest Quarter
Mean Temperature of Warmest Quarter
Mean Temperature of Coldest Quarter
Annual Precipitation
Precipitation of Wettest Month
Precipitation of Driest Month
Precipitation Seasonality
Precipitation of Wettest Quarter
Precipitation of Driest Quarter
Precipitation of Warmest Quarter
Precipitation of Coldest Quarter
Crop land
Other land
Pasture land
Secondary Land
Corr
1.0
0.5
0.0
-0.5
-1.0

(c)

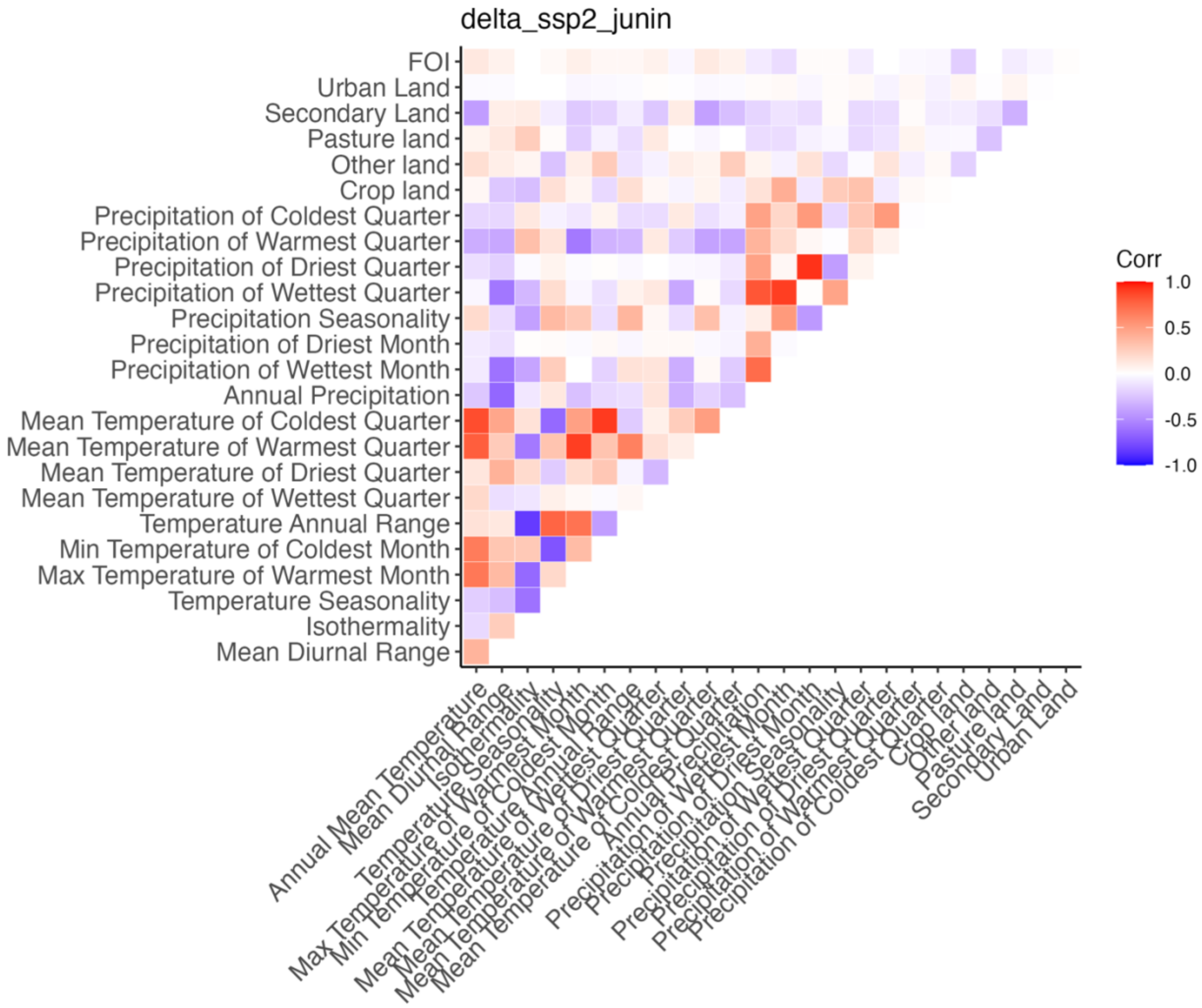

delta_ssp2_junin
FOI
Urban Land
Secondary Land
Pasture land
Other land
Crop land
Precipitation of Coldest Quarter
Precipitation of Warmest Quarter
Precipitation of Driest Quarter
Precipitation of Wettest Quarter
Precipitation Seasonality
Precipitation of Driest Month
Precipitation of Wettest Month
Annual Precipitation
Mean Temperature of Coldest Quarter
Mean Temperature of Warmest Quarter
Mean Temperature of Driest Quarter
Mean Temperature of Wettest Quarter
Temperature Annual Range
Min Temperature of Coldest Month
Max Temperature of Warmest Month
Temperature Seasonality
Isothermality
Mean Diurnal Range
Annual Mean Temperature
Corr
1.0
0.5
0.0
-0.5
-1.0

(d)

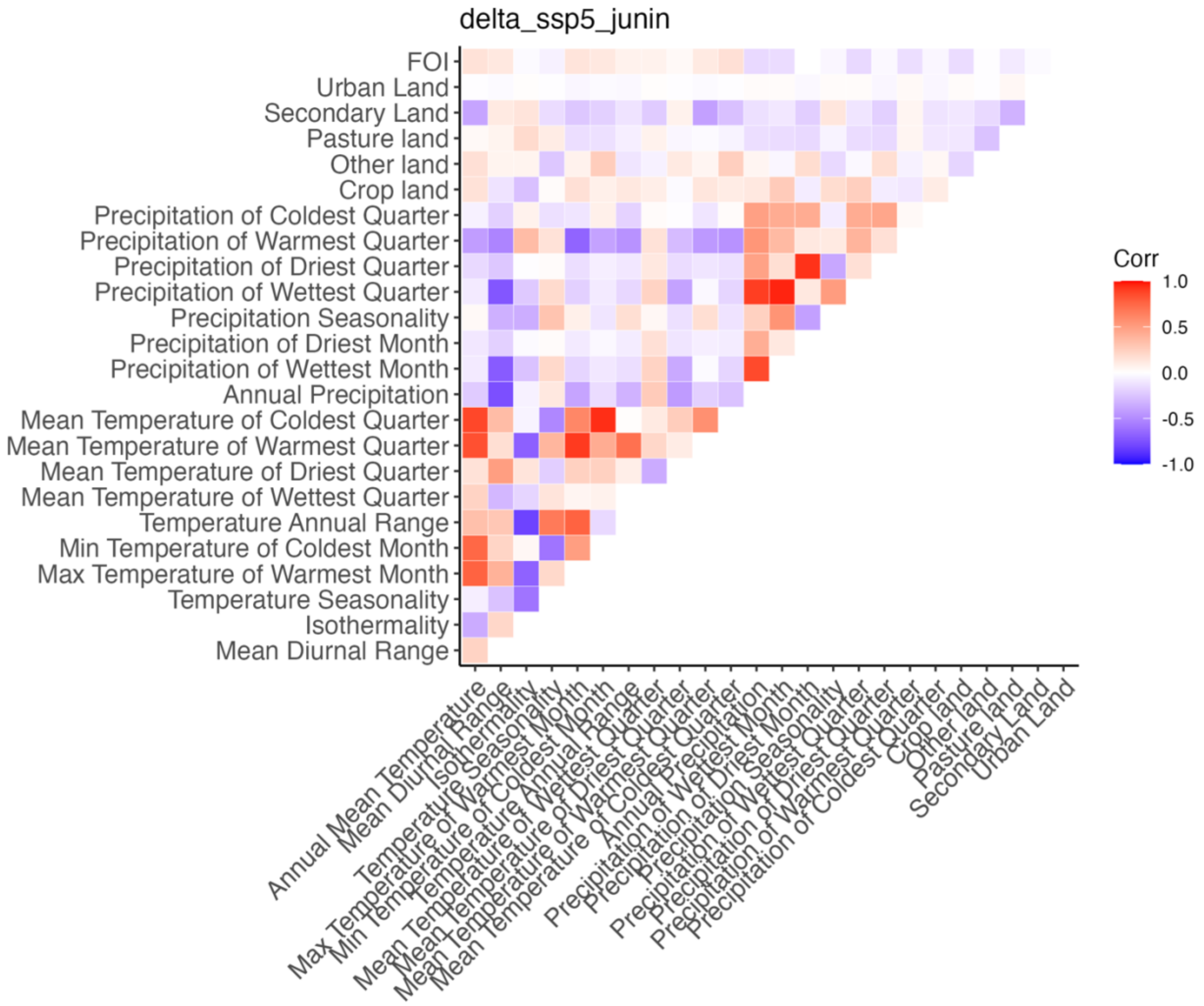

(e)

Fig 3.2 Pearson's correlation heatmap of predictive features for JUNV in –

(a) current climate scenario, (b) SSP 2-4.5 scenario, (c) SSP 5-8.5 scenario, (d) difference between current and SSP 2-4.5 values of the features ($\Delta_f^{ssp\ 2-4.5}$) and (e) difference between current and SSP 5-8.5 values of the features ($\Delta_f^{ssp\ 5-8.5}$)

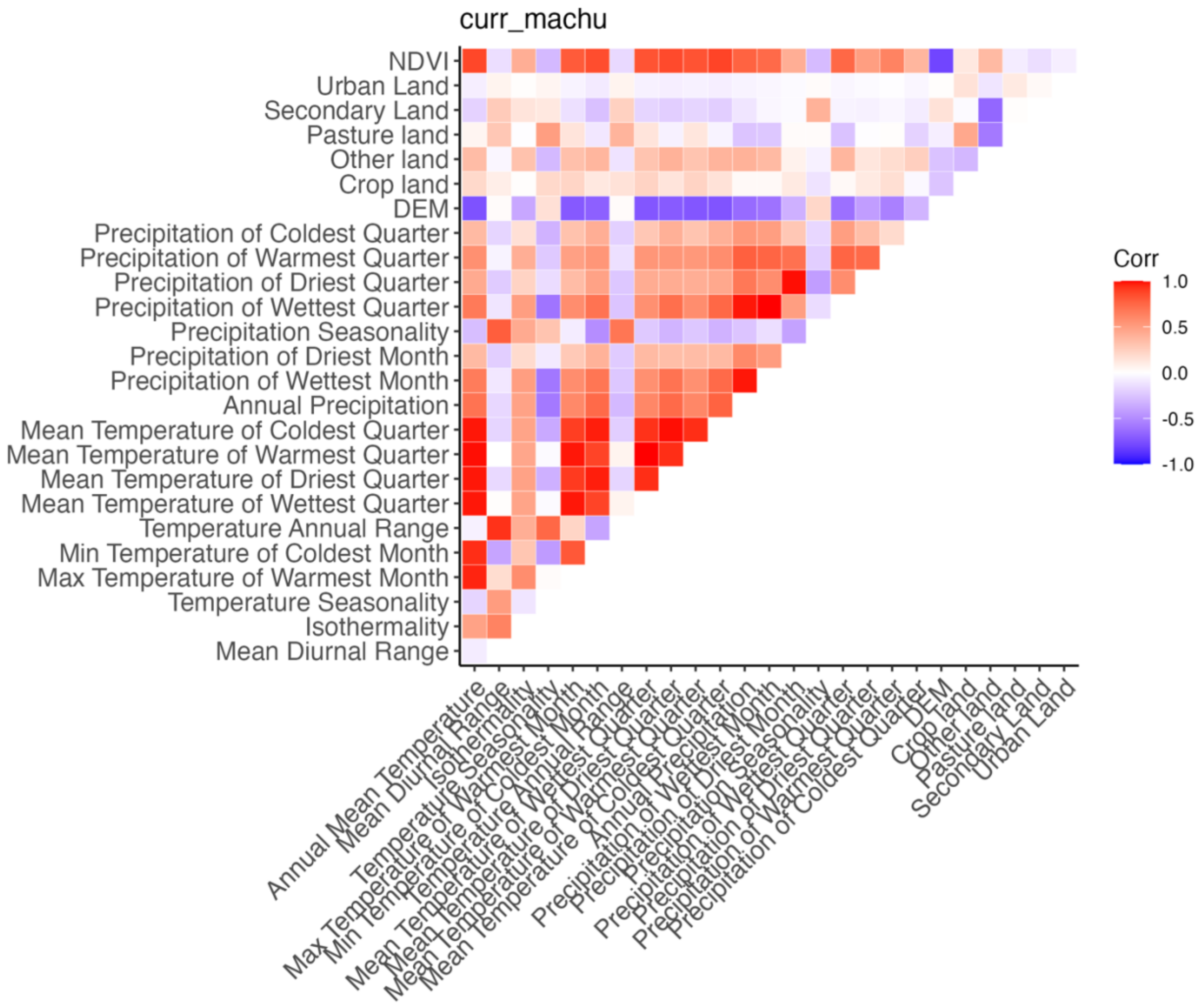
curr_machu
NDVI
Urban Land
Secondary Land
Pasture land
Other land
Crop land
DEM
Precipitation of Coldest Quarter
Precipitation of Warmest Quarter
Precipitation of Driest Quarter
Precipitation of Wettest Quarter
Precipitation Seasonality
Precipitation of Driest Month
Precipitation of Wettest Month
Annual Precipitation
Mean Temperature of Coldest Quarter
Mean Temperature of Warmest Quarter
Mean Temperature of Driest Quarter
Mean Temperature of Wettest Quarter
Temperature Annual Range
Min Temperature of Coldest Month
Max Temperature of Warmest Month
Temperature Seasonality
Isothermality
Mean Diurnal Range
Annual Mean Temperature
Corr
1.0
0.5
0.0
-0.5
-1.0

(a)

Supplementary Material: Climate-driven changes in zoonotic risk of arenaviral hemorrhagic fevers in South America

PS. Kulkarni, NY. Flores-Perez, M. Uhart, BH. Bird, CK. Johnson, PS. Pandit.

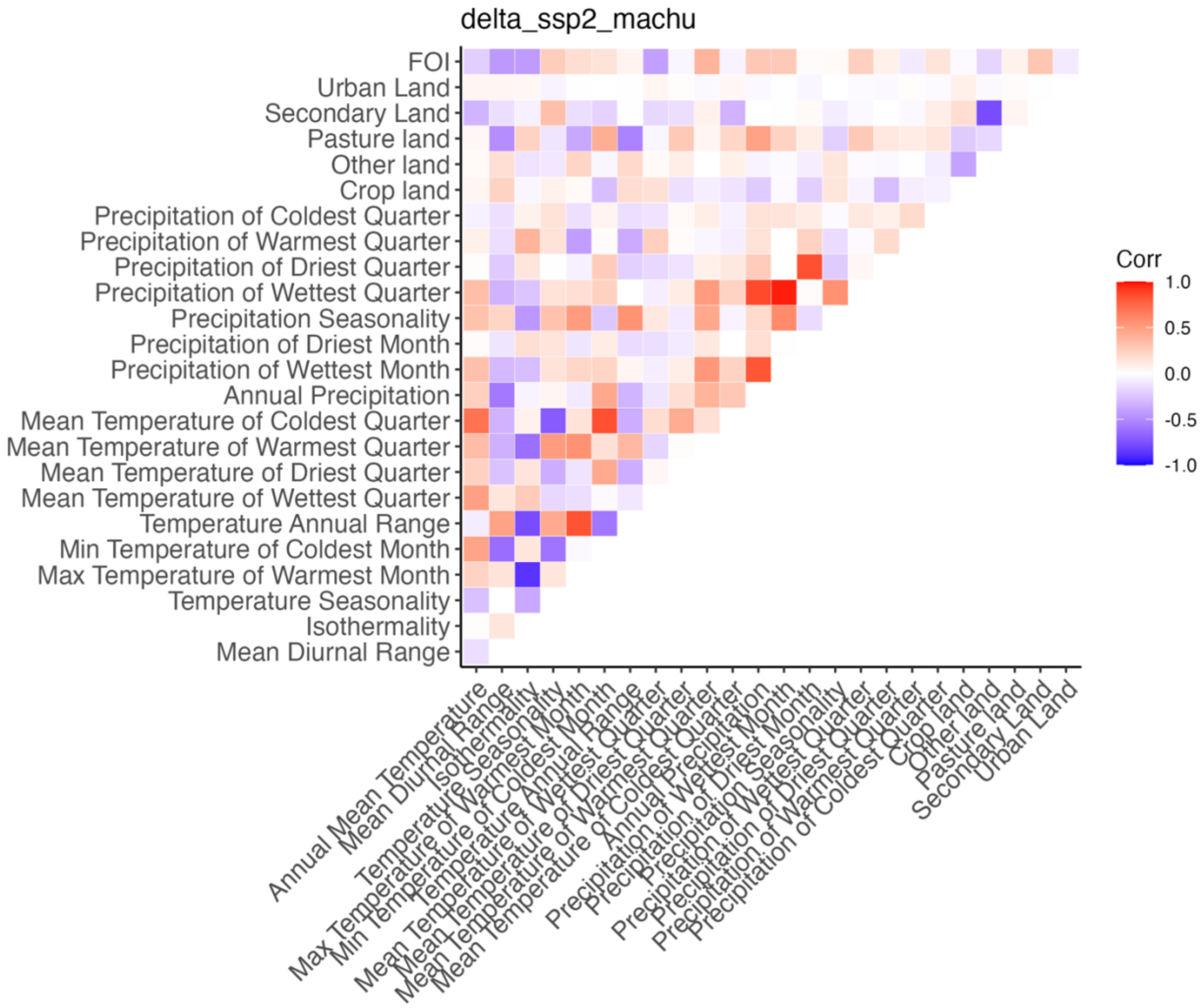

(b)

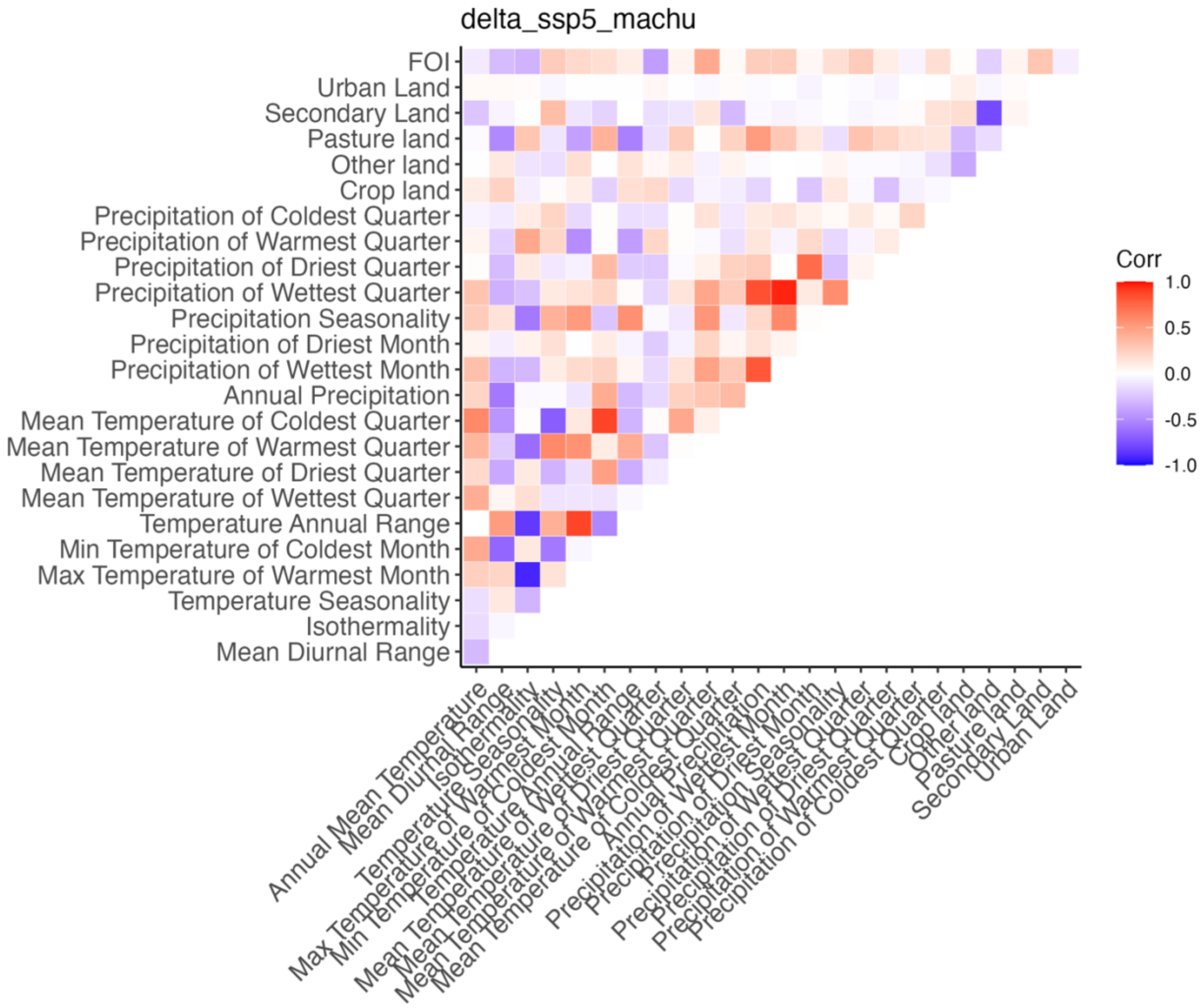
delta_ssp5_machu
FOI
Urban Land
Secondary Land
Pasture land
Other land
Crop land
Precipitation of Coldest Quarter
Precipitation of Warmest Quarter
Precipitation of Driest Quarter
Precipitation of Wettest Quarter
Precipitation Seasonality
Precipitation of Driest Month
Precipitation of Wettest Month
Annual Precipitation
Mean Temperature of Coldest Quarter
Mean Temperature of Warmest Quarter
Mean Temperature of Driest Quarter
Mean Temperature of Wettest Quarter
Temperature Annual Range
Min Temperature of Coldest Month
Max Temperature of Warmest Month
Temperature Seasonality
Isothermality
Mean Diurnal Range
Annual Mean Temperature
Corr
1.0
0.5
0.0
-0.5
-1.0

(c)

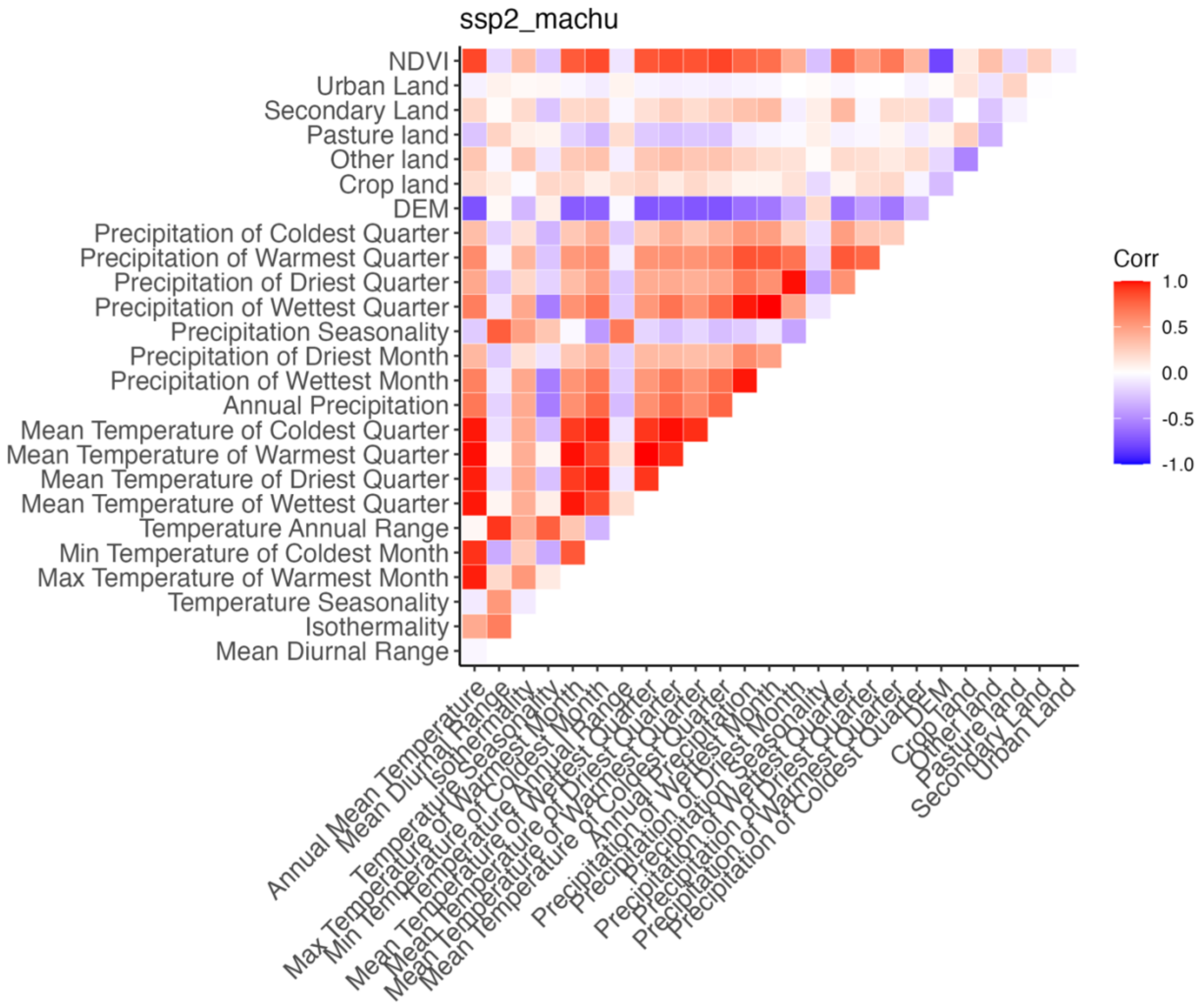
ssp2_machu
NDVI
Urban Land
Secondary Land
Pasture land
Other land
Crop land
DEM
Precipitation of Coldest Quarter
Precipitation of Warmest Quarter
Precipitation of Driest Quarter
Precipitation of Wettest Quarter
Precipitation Seasonality
Precipitation of Driest Month
Precipitation of Wettest Month
Annual Precipitation
Mean Temperature of Coldest Quarter
Mean Temperature of Warmest Quarter
Mean Temperature of Driest Quarter
Mean Temperature of Wettest Quarter
Temperature Annual Range
Min Temperature of Coldest Month
Max Temperature of Warmest Month
Temperature Seasonality
Isothermality
Mean Diurnal Range
Annual Mean Temperature
Corr
1.0
0.5
0.0
-0.5
-1.0

(d)

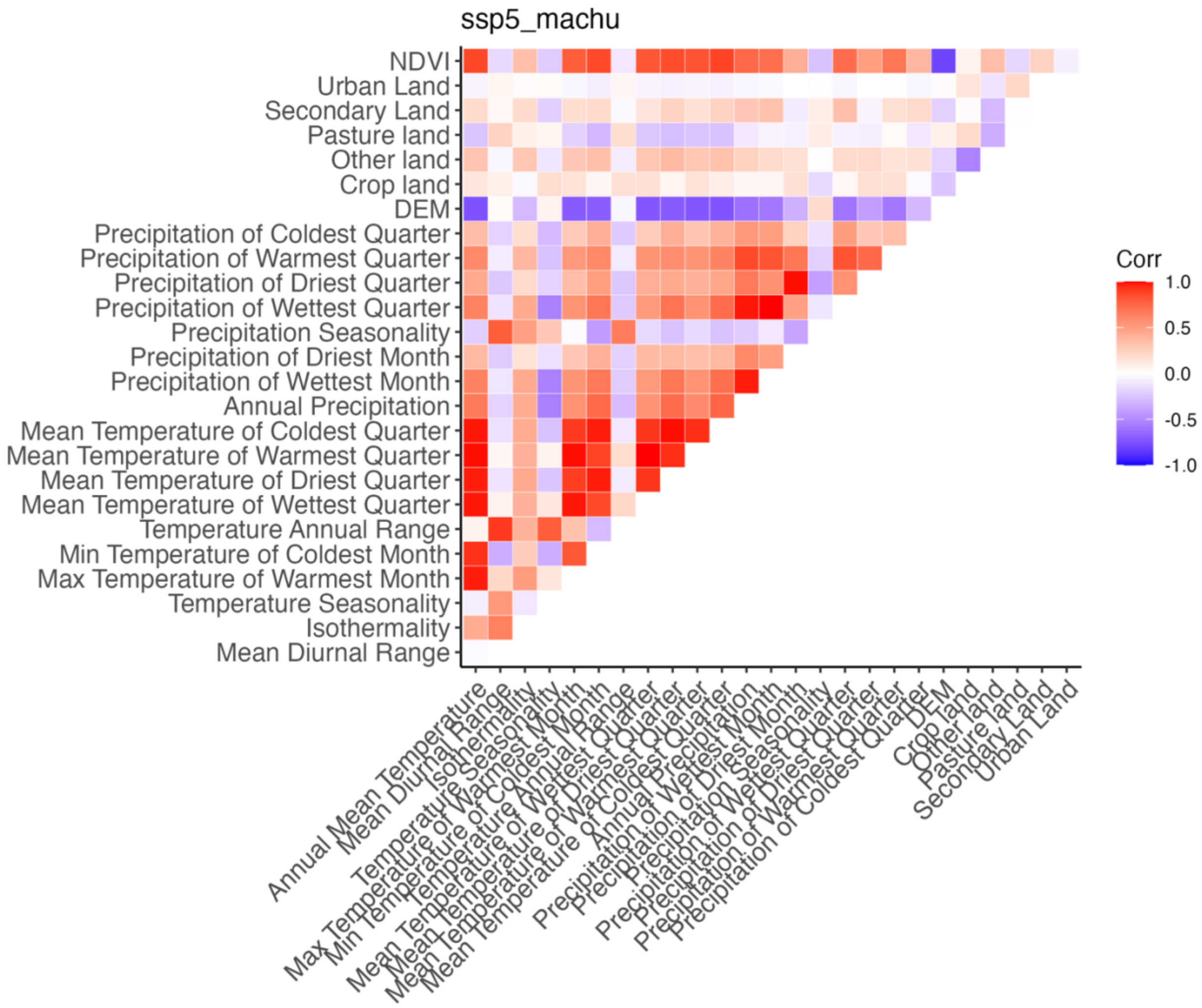

(e)

Fig 3.3 Pearson's correlation heatmap of predictive features for MACV in–

(a) current climate scenario, (b) SSP 2-4.5 scenario, (c) SSP 5-8.5 scenario, (d) difference between current and SSP 2-4.5 values of the features ($\Delta_f^{ssp\ 2-4.5}$) and (e) difference between current and SSP 5-8.5 values of the features ($\Delta_f^{ssp\ 5-8.5}$)

References

1. Barua, S., Dénes, A. & Ibrahim, M. A. A seasonal model to assess intervention strategies for preventing periodic recurrence of Lassa fever. Heliyon **7**, (2021).
2. Radoshitzky, S. R. & de la Torre, J. C. Human Pathogenic Arenaviruses (Arenaviridae). in Encyclopedia of Virology (Fourth Edition) (eds. Bamford, D. H. & Zuckerman, M.) 507–517 (Academic Press, Oxford, 2019). doi:10.1016/B978-0-12-814515-9.00014-X.
3. Gedeon, T., Bodelón, C. & Kuenzi, A. Hantavirus Transmission in Sylvan and Peridomestic Environments. Bull. Math. Biol. **72**, 541–564 (2010).
4. Fichet-Calvet, E., Becker-Ziaja, B., Koivogui, L. & Günther, S. Lassa Serology in Natural Populations of Rodents and Horizontal Transmission. Vector-Borne Zoonotic Dis. **14**, 665–674 (2014).
5. Abdullahi, M. B., Doko, U. C. & Mamuda, M. Sensitivity analysis in a Lassa fever deterministic mathematical model. AIP Conf. Proc. **1660**, 050050 (2015).
6. Obabiyi, O. s & Onifade, A. MATHEMATICAL MODEL FOR LASSA FEVER TRANSMISSION DYNAMICS WITH VARIABLE HUMAN AND RESERVOIR POPULATION. **16**, 67–91 (2017).
7. Abdulhamid, A. & Hussainia, N. Effects of quarantine on transmission dynamics of Lassa fever. Bayero J. Pure Appl. Sci. **11**, 397–407 (2018).
8. Liu, J. & School of Science, Xi'an Polytechnic University, Xi'an, Shaanxi 710048, P.R. China. Threshold dynamics of a time-delayed hantavirus infection model in periodic environments. Math. Biosci. Eng. **16**, 4758–4776 (2019).
9. Density dependence and persistence of Morogoro arenavirus transmission in a fluctuating population of its reservoir host - Mariën - 2020 - Journal of Animal Ecology - Wiley Online Library. https://besjournals.onlinelibrary.wiley.com/doi/10.1111/1365-2656.13107.
10. Mathematical Modelling and Analysis of Transmission Dynamics of Lassa Fever - Bakare - 2020 - Journal of Applied Mathematics - Wiley Online Library. https://onlinelibrary.wiley.com/doi/10.1155/2020/6131708.
11. Chitnis, N., Hyman, J. M. & Cushing, J. M. Determining Important Parameters in the Spread of Malaria Through the Sensitivity Analysis of a Mathematical Model. Bull. Math. Biol. **70**, 1272–1296 (2008).
12. Musa, S. S. et al. Mechanistic modelling of the large-scale Lassa fever epidemics in Nigeria from 2016 to 2019. J. Theor. Biol. **493**, 110209 (2020).
13. Dynamical System Analysis of a Lassa Fever Model with Varying Socioeconomic Classes - Onah - 2020 - Journal of Applied Mathematics - Wiley Online Library. https://onlinelibrary.wiley.com/doi/10.1155/2020/2601706.
14. Peter, O. J. et al. Global Stability Analysis of Typhoid Fever Model. Adv. Syst. Sci. Appl. **20**, 20–31 (2020).
15. Peter, O. J. et al. Modelling and optimal control analysis of Lassa fever disease. Inform. Med. Unlocked **20**, 100419 (2020).

16. Basinski, A. J. et al. Bridging the gap: Using reservoir ecology and human serosurveys to estimate Lassa virus spillover in West Africa. PLOS Comput. Biol. **17**, e1008811 (2021).
17. Ibrahim, M. A. & Dénes, A. A mathematical model for Lassa fever transmission dynamics in a seasonal environment with a view to the 2017–20 epidemic in Nigeria. Nonlinear Anal. Real World Appl. **60**, 103310 (2021).
18. Ojo, M. M., Gbadamosi, B., Benson, T. O., Adebimpe, O. & Georgina, A. L. Modeling the dynamics of Lassa fever in Nigeria. J. Egypt. Math. Soc. **29**, 16 (2021).
19. Okolo, P. N., Nwabufo, I. V. & Abu, O. Mathematical model for the transmission dynamics of Lassa fever with control. Sci. World J. **15**, 62–68 (2020).
20. Omoloye, M. A., Sanusi, A. O. & Sanusi, I. O. Modeling and Sensitivity Analysis of Dynamical Transmission of Lassa Fever. Int. J. Res. Rev. **8**, 531–539 (2021).
21. Hamam, H. et al. Deciphering the enigma of Lassa virus transmission dynamics and strategies for effective epidemic control through awareness campaigns and rodenticides. Sci. Rep. **14**, 18079 (2024).
22. Pranav S. Kulkarni (uid: vetpsk). Derived dataset for Kulkarni et al. The Global Biodiversity Information Facility https://doi.org/10.15468/DD.6U8HUK (2025).
23. Booth, T. H., Nix, H. A., Busby, J. R. & Hutchinson, M. F. BIOCLIM : the first species distribution modelling package, its early applications and relevance to most current MAXENT studies. Divers. Distrib. **20**, 1–9 (2014).
24. Fick, S. E. & Hijmans, R. J. WorldClim 2: new 1-km spatial resolution climate surfaces for global land areas. Int. J. Climatol. **37**, 4302–4315 (2017).
25. IUCN. The IUCN Red List of Threatened Species. IUCN Red List of Threatened Species https://www.iucnredlist.org/en (2025).
26. Flores-Pérez, N., Kulkarni, P., Uhart, M. & Pandit, P. S. Climate Change Impact on Human-Rodent Interfaces: Modeling Junin Virus Reservoir Shifts. EcoHealth (2025) doi:10.1007/s10393-025-01723-z.
27. El-Gabbas, A. & Dormann, C. F. Wrong, but useful: regional species distribution models may not be improved by range-wide data under biased sampling. Ecol. Evol. **8**, 2196–2206 (2018).
28. Elith, J. & Leathwick, J. R. Species Distribution Models: Ecological Explanation and Prediction Across Space and Time. Annu. Rev. Ecol. Evol. Syst. **40**, 677–697 (2009).
29. Guillaumot, C., Martin, A., Eléaume, M. & Saucède, T. Methods for improving species distribution models in data-poor areas: example of sub-Antarctic benthic species on the Kerguelen Plateau. Mar. Ecol. Prog. Ser. **594**, 149–164 (2018).
30. Wisz, M. S. et al. Effects of sample size on the performance of species distribution models. Divers. Distrib. **14**, 763–773 (2008).
31. Grenouillet, G., Buisson, L., Casajus, N. & Lek, S. Ensemble modelling of species distribution: the effects of geographical and environmental ranges. Ecography **34**, 9–17 (2011).
32. Guo, C. et al. Uncertainty in ensemble modelling of large-scale species distribution: Effects from species characteristics and model techniques. Ecol. Model. **306**, 67–75 (2015).

33. Chen, H., Sun, J., Lin, W. & Xu, H. Comparison of CMIP6 and CMIP5 models in simulating climate extremes. Sci. Bull. **65**, 1415–1418 (2020).

34. Riahi, K. et al. The Shared Socioeconomic Pathways and their energy, land use, and greenhouse gas emissions implications: An overview. Glob. Environ. Change **42**, 153–168 (2017).

35. Milazzo, M. L. et al. Transmission of Guanarito and Pirital Viruses among Wild Rodents, Venezuela - Volume 17, Number 12—December 2011 - Emerging Infectious Diseases journal - CDC. doi:10.3201/eid1712.110393.

36. Tesh, R. B. et al. Field studies on the epidemiology of Venezuelan hemorrhagic fever: implication of the cotton rat Sigmodon alstoni as the probable rodent reservoir. Am. J. Trop. Med. Hyg. **49**, 227–235 (1993).

37. Weaver, S. C. et al. Guanarito Virus (Arenaviridae) Isolates from Endemic and Outlying Localities in Venezuela: Sequence Comparisons among and within Strains Isolated from Venezuelan Hemorrhagic Fever Patients and Rodents. Virology **266**, 189–195 (2000).

38. Salazar-Bravo, J., Ruedas, L. A. & Yates, T. L. Mammalian Reservoirs of Arenaviruses. in Arenaviruses I: The Epidemiology, Molecular and Cell Biology of Arenaviruses (ed. Oldstone, M. B. A.) 25–63 (Springer, Berlin, Heidelberg, 2002). doi:10.1007/978-3-642-56029-3_2.

39. Mills, J. N. et al. A Longitudinal Study of Junin Virus Activity in the Rodent Reservoir of Agrentine Hemorrhagic Fever. Am. J. Trop. Med. Hyg. **47**, 749–763 (1992).

40. Morales, M. A. et al. Evaluation of an enzyme-linked immunosorbent assay for detection of antibodies to Junin virus in rodents. J. Virol. Methods **103**, 57–66 (2002).

41. Salazar-Bravo, J. et al. Natural nidality in Bolivian hemorrhagic fever and the systematics of the reservoir species. Infect. Genet. Evol. **1**, 191–199 (2002).

42. González-Ittig, R. E. et al. Molecular systematics and biogeographic insights of the Calomys callosus complex (Rodentia, Cricetidae). Zool. Scr. **51**, 498–521 (2022).

43. Tapia-Ramírez, G. et al. A Review of Mammarenaviruses and Rodent Reservoirs in the Americas. EcoHealth **19**, 22–39 (2022).

44. Lendino, A., Castellanos, A. A., Pigott, D. M. & Han, B. A. A review of emerging health threats from zoonotic New World mammarenaviruses. BMC Microbiol. **24**, 115 (2024).

45. Meerburg, B. G., Singleton, G. R. & Kijlstra, A. Rodent-borne diseases and their risks for public health. Crit. Rev. Microbiol. **35**, 221–270 (2009).

46. Han, B. A., O'Regan, S. M., Paul Schmidt, J. & Drake, J. M. Integrating data mining and transmission theory in the ecology of infectious diseases. Ecol. Lett. **23**, 1178–1188 (2020).

47. Plowright, R. K. et al. Pathways to zoonotic spillover. Nat. Rev. Microbiol. **15**, 502–510 (2017).

48. Becker, D. J. et al. Optimising predictive models to prioritise viral discovery in zoonotic reservoirs. Lancet Microbe **3**, e625–e637 (2022).

49. Huang, Z. Y. X., Langevelde, F. V., Estrada-Peña, A., Suzán, G. & Boer, W. F. D. The diversity–disease relationship: evidence for and criticisms of the dilution effect. Parasitology **143**, 1075–1086 (2016).

50. García-Peña, G. E. et al. Land-use change and rodent-borne diseases: hazards on the shared socioeconomic pathways. Philos. Trans. R. Soc. B Biol. Sci. **376**, 20200362 (2021).
51. Oliver, T. H. et al. Population density but not stability can be predicted from species distribution models. J. Appl. Ecol. **49**, 581–590 (2012).
52. A. Lee-Yaw, J., L. McCune, J., Pironon, S. & N. Sheth, S. Species distribution models rarely predict the biology of real populations. Ecography **2022**, e05877 (2022).
53. GBIF. What is GBIF? https://www.gbif.org/what-is-gbif.
54. Han, B. Supplementary data tables: Integrating data mining and transmission theory in the ecology of infectious diseases. Preprint at https://doi.org/10.25390/CARYINSTITUTE.C.4912389.V1 (2020).
55. Da Re, D. et al. USE it: Uniformly sampling pseudo-absences within the environmental space for applications in habitat suitability models. Methods Ecol. Evol. **14**, 2873–2887 (2023).
56. Moudrý, V. et al. Optimising occurrence data in species distribution models: sample size, positional uncertainty, and sampling bias matter. Ecography **2024**, e07294 (2024).
57. Barbet-Massin, M., Jiguet, F., Albert, C. H. & Thuiller, W. Selecting pseudo-absences for species distribution models: how, where and how many? Methods Ecol. Evol. **3**, 327–338 (2012).
58. Senay, S. D., Worner, S. P. & Ikeda, T. Novel Three-Step Pseudo-Absence Selection Technique for Improved Species Distribution Modelling. PLOS ONE **8**, e71218 (2013).
59. Broussin, J., Mouchet, M. & Goberville, E. Generating pseudo-absences in the ecological space improves the biological relevance of response curves in species distribution models. Ecol. Model. **498**, 110865 (2024).
60. Chapare haemorrhagic fever- the Plurinational State of Bolivia. https://www.who.int/emergencies/disease-outbreak-news/item/2025-DON553.
